# Association and Prevalence of Lower Urinary Tract Symptoms in Individuals with Sarcopenia: A Systematic Review and Meta-Analysis

**DOI:** 10.3390/medicina61071214

**Published:** 2025-07-03

**Authors:** Lek-Hong Tan, Eric Chieh-Lung Chou

**Affiliations:** Department of Urology, China Medical University Hospital, Taichung 40447, Taiwan

**Keywords:** sarcopenia, lower urinary tract symptoms (LUTSs), urinary incontinence, muscle strength, lean mass, SARC-F, AWGS, EWGSOP, aging, systematic review and meta-analysis

## Abstract

*Background and Objectives*: Sarcopenia and lower urinary tract symptoms (LUTSs) are both prevalent among older adults and may share underlying pathophysiological mechanisms. However, their association has not been systematically quantified. This systematic review and meta-analysis aimed to evaluate the association between sarcopenia and LUTSs, including the pooled estimates of prevalence and odds ratios (ORs), and to explore the influence of diagnostic definitions and study-level factors. *Materials and Methods*: A comprehensive literature search was conducted using PubMed and Embase for studies published between 1 January 2000 and 26 April 2025. This study adhered to PRISMA and MOOSE guidelines and was registered in PROSPERO (CRD420251037459). Eligible observational studies reported LUTS prevalence or ORs in individuals with sarcopenia, low muscle strength (LMS), low lean mass (LLM), low gait speed (LGS), or sarcopenia risk identified by SARC-F (score ≥4). Pooled ORs and prevalence rates were calculated using a random-effects model. Subgroup analyses were performed based on sarcopenia definitions—Asian Working Group for Sarcopenia (AWGS) and European Working Group on Sarcopenia in Older People (EWGSOP)—as well as LUTS subtypes and diagnostic components. Univariate meta-regression assessed associations with age, BMI, sex distribution, WHO region, and risk of bias. *Results*: Twenty-five studies comprising 84,484 participants were included. Sarcopenia was significantly associated with LUTSs (pooled OR = 1.78; 95% CI: 1.29–2.45; *p* < 0.001), with a pooled LUTS prevalence of 43.2% (95% CI: 26.9–61.0%). Stronger associations were observed in studies using AWGS diagnostic criteria (OR = 2.24; 95% CI: 1.41–3.56; *p* = 0.001), in those evaluating severe sarcopenia (OR = 1.66; 95% CI: 1.03–2.68; *p* = 0.038), and in institutionalized populations (OR = 3.68; 95% CI: 2.18–6.24; *p* < 0.001) compared to community-dwelling populations (OR = 1.43; 95% CI: 1.06–1.92; *p* = 0.018). Sarcopenia risk identified by SARC-F (score ≥4) showed the strongest association with LUTSs (OR = 3.20; 95% CI: 1.92–5.33; *p* < 0.001). Significant associations were also found for LLM (OR = 1.52; 95% CI: 1.19–1.95; *p* = 0.001) and LGS (OR = 1.37; 95% CI: 1.06–1.76; *p* = 0.015), but not for LMS (OR = 0.94; 95% CI: 0.47–1.89; *p* = 0.871). Exploratory analyses comparing LLM diagnostic modalities—including standardized criteria (ASMI, ASM/BMI), imaging-based methods (SMI, PMA), and surrogate measures (calf circumference)—revealed no significant differences (all *p* > 0.05). Heterogeneity was high (I^2^ > 90%). Egger’s test indicated no evidence of publication bias (*p* = 0.838), and trim-and-fill analysis did not affect the pooled estimates. *Conclusions*: Sarcopenia—particularly in its severe forms—is significantly associated with LUTSs. Additionally, individuals who screened positive for sarcopenia using the SARC-F tool demonstrated a heightened risk of LUTSs. Subgroup analyses revealed a stronger association in institutionalized populations, suggesting that care setting may modify risk. These findings underscore the importance of assessing muscle health in older adults with urinary symptoms. Standardization of diagnostic criteria and longitudinal studies are needed to clarify causality and guide targeted interventions.

## 1. Introduction

Sarcopenia, characterized by age-related loss of skeletal muscle mass and function, is a prevalent condition among older adults, with estimates suggesting it affects approximately 10% to 27% of individuals over 60 years of age [1,2]. This condition is associated with adverse outcomes such as frailty, falls, and decreased quality of life [3,4].

Lower urinary tract symptoms (LUTSs)—including urinary incontinence, urgency, nocturia, and overactive bladder (OAB)—are also highly prevalent in aging populations, with up to 70% of men aged 80 and above experiencing LUTSs [5,6,7,8,9,10,11,12]. The frequent co-occurrence of sarcopenia and LUTSs suggests possible shared risk factors, such as aging, hormonal changes, and decreased physical activity [13,14]. Notably, frailty has been strongly associated with diminished bladder function, including detrusor underactivity, impaired contractility, and high post-void residual volumes, as supported by urodynamic studies and machine learning-based clinical prediction models [15,16]. Furthermore, population-based analyses and longitudinal cohort data have shown that frailty correlates with the increased prevalence and progression of lower urinary tract symptoms (LUTSs), reinforcing the connection between musculoskeletal decline and urinary dysfunction [17,18].

Emerging evidence points to a significant association between sarcopenia and various forms of urinary incontinence. For instance, research has demonstrated that sarcopenia is independently associated with urinary incontinence in adult women under 60, highlighting the role of muscle mass and strength in maintaining continence [19]. Additionally, studies have found that low lean mass is linked to a higher risk of LUTSs in men aged 40 and above [14,20,21].

Despite emerging evidence, the relationship between sarcopenia and lower urinary tract symptoms (LUTSs) remains underexplored, and no comprehensive synthesis has systematically evaluated both the strength of their association and the broader clinical context. Clarifying this relationship is important, as it may reveal shared pathophysiological mechanisms and inform preventive or therapeutic strategies aimed at preserving function and improving quality of life in older adults.

While several individual studies have reported associations between sarcopenia (or its components, such as low lean mass or grip strength) and various urinary dysfunctions—including LUTSs and incontinence—these have not been synthesized systematically. To our knowledge, this is the first systematic review and meta-analysis to comprehensively examine the association between sarcopenia and LUTSs across diverse populations, definitions, and methodological approaches. Therefore, this systematic review and meta-analysis primarily aims to quantify the association between sarcopenia and LUTSs by pooling odds ratio estimates across studies. In addition, we examine the prevalence of LUTSs among individuals with sarcopenia and conduct secondary and exploratory analyses to evaluate the influence of diagnostic criteria, sarcopenia components (e.g., low muscle strength, low lean mass, gait speed), and methodological factors on the observed associations.

## 2. Methods

### 2.1. Search Strategy and Selection Criteria

This systematic review and meta-analysis was conducted in accordance with the Preferred Reporting Items for Systematic Reviews and Meta-Analyses (PRISMA) [22] and Meta-analysis of Observational Studies in Epidemiology (MOOSE) guidelines [23]. The primary objective was to evaluate the association between sarcopenia and lower urinary tract symptoms (LUTSs), with secondary analyses examining the individual components of sarcopenia, including low muscle strength (LMS), low lean mass (LLM), low gait speed (LGS), and sarcopenia risk as identified by the SARC-F questionnaire (defined as a score ≥4), in accordance with the recommendations from the AWGS and EWGSOP [24,25].

A comprehensive literature search was conducted using PubMed/MEDLINE and Embase to identify relevant studies published between 1 January 2000 and 26 April 2025. The search strategy focused on two primary conceptual domains: sarcopenia and lower urinary tract symptoms (LUTSs). For sarcopenia, we used both Medical Subject Headings (MeSHs) and free-text terms, including “Sarcopenia”[MeSH], “Muscular Atrophy”[MeSH], “sarcopenia*”, “muscular atroph*”, “neurogenic muscular atroph*”, “low lean mass”, “low muscle mass”, “muscle wasting”, and “age-related muscle loss”. For LUTSs, the search included MeSH terms and keywords such as “Lower Urinary Tract Symptoms”[MeSH], “Dysuria”[MeSH], “Urinary Retention”[MeSH], “Nocturia”[MeSH], “Urinary Incontinence”[MeSH], and “urinary bladder, underactive”[MeSH], as well as synonyms and variants including “lower urinary tract symptom*”, “LUTS”, “dysuria”, “urination disorder*”, “nocturia”, “nycturia”, “overactive bladder*”, “underactive bladder*”, “overactive detrusor*”, “underactive detrusor*”, “hypotonic bladder*”, “stress incontinence”, and “urge incontinence”.

To exclude non-human studies, we applied the standard PubMed animal filter: (“animals”[MeSH Terms] NOT (“humans”[MeSH Terms] AND “animals”[MeSH Terms])). The final search strategy combined the sarcopenia- and LUTS-related terms using Boolean operators, applied a publication date limit from 2000 to 2025, and excluded animal studies. No language restrictions were applied. Articles in languages other than English were translated using Google Translate and ChatGPT-4.0 where necessary. Although only two databases (PubMed and Embase) were used—due to institutional access limitations—these platforms provide the extensive coverage of biomedical literature. To mitigate the risk of missing relevant studies, we also manually reviewed the reference lists of all included articles and related reviews. The complete PubMed/MEDLINE and Embase search strategies are provided in Supplementary Tables S1 and S2, respectively.

Study eligibility was determined based on the following inclusion criteria: (1) observational study design (cross-sectional, prospective, or retrospective cohort); (2) adult participants assessed for sarcopenia, LMS, LLM, LGS, or sarcopenia risk (SARC-F ≥ 4); and (3) quantitative reporting of odds ratios (ORs) and/or prevalence data for LUTSs. Studies were excluded if they were reviews, editorials, or case reports, involved pediatric populations, or focused on perioperative cohorts in which surgical interventions might confound LUTS outcomes. When multiple studies involved overlapping populations, the most comprehensive dataset or the study with the largest sample size was selected.

The screening and selection process was conducted independently by two reviewers (L.H. Tan and C.L. Chou) using Rayyan, a web-based tool for systematic review management, to facilitate blinded screening and conflict resolution. Any discrepancies were resolved through discussion and consensus.

This review was conducted in accordance with a registered protocol on PROSPERO (CRD420251037459).

### 2.2. Definition of Sarcopenia and Its Components

Sarcopenia was defined according to the diagnostic criteria of major international consensus groups, including the Asian Working Group for Sarcopenia (AWGS 2014 and 2019) [25,26] and the European Working Group on Sarcopenia in Older People (EWGSOP1 and EWGSOP2) [24,27]. The details of the diagnostic thresholds for muscle mass, strength, and performance are summarized in Supplementary Table S9. One study in our analysis employed modified diagnostic criteria that required the simultaneous presence of all three components: ASMI < 5.5 kg/m^2^, gait speed < 1.0 m/s, and grip strength < 20.5 kg [28]. All criteria were recorded as reported by the original study authors; no reinterpretation or reclassification was performed.

### 2.3. Definition of Sarcopenia Diagnostic Components

Low muscle strength (LMS) was predominantly assessed using handgrip strength across the included studies, with threshold values ranging from <16 kg to <22 kg depending on the population characteristics and the diagnostic criteria referenced. Low lean mass (LLM) was evaluated using various assessment methods. The most commonly used indicator was the appendicular skeletal muscle mass index (ASMI), with cutoffs ranging from <5.5 to <5.7 kg/m^2^ in women and up to <7.26 kg/m^2^ in men, in accordance with the criteria established by the Asian Working Group for Sarcopenia (AWGS) and the European Working Group on Sarcopenia in Older People (EWGSOP). Several studies used appendicular skeletal muscle mass adjusted for body mass index (ASM/BMI), applying thresholds of <0.789 for men and <0.512 for women based on the Foundation for the National Institutes of Health (FNIH) Sarcopenia Project. A closely related alternative cutoff of <0.793 was also used in some studies. Two studies employed calf circumference as a surrogate marker of muscle mass, using thresholds of <34 cm for men and <33 cm for women, consistent with AWGS 2019 recommendations in resource-limited settings where imaging tools such as DXA or BIA were unavailable.

Imaging-based assessments of lean mass included the skeletal muscle index (SMI), calculated from computed tomography (CT) images. One study defined low SMI as <43 cm^2^/m^2^ for individuals with a BMI <25, or <53 cm^2^/m^2^ for those with a BMI ≥25. Another study assessed lean mass using psoas muscle area (PMA), though no specific cutoff value was reported. One additional study evaluated skeletal muscle mass (SMM) normalized by height^2^, weight, or BMI, using thresholds of <7.4 kg/m^2^, <33.6%, and <0.823, respectively. Low gait speed (LGS), an indicator of physical performance, was defined using cutoffs ranging from <0.95 m/s to <1.1 m/s.

Although LMS, LLM, and LGS are essential diagnostic components of sarcopenia in consensus guidelines (e.g., AWGS, EWGSOP), they were analyzed individually in this study to explore their independent associations with LUTSs. These components were not treated as equivalent to a formal diagnosis of sarcopenia. Instead, they were assessed in separate secondary or exploratory analyses to provide a more granular understanding of which specific muscle-related impairments might contribute most significantly to lower urinary tract dysfunction.

### 2.4. Sarcopenia Risk Screening Using SARC-F

Sarcopenia risk was assessed using the SARC-F questionnaire, a self-reported screening tool recommended by both the AWGS and EWGSOP guidelines. A score of ≥4 was used to indicate an elevated risk of sarcopenia. Although the SARC-F is valuable for identifying individuals who may require further evaluation, it is not sufficient alone to confirm a diagnosis of sarcopenia or probable sarcopenia. In our analysis, studies using SARC-F ≥4 were analyzed separately and not pooled with studies involving confirmed sarcopenia diagnosis. This distinction was maintained throughout the synthesis to avoid conflating sarcopenia risk with established diagnostic constructs.

### 2.5. Definition of Lower Urinary Tract Symptoms (LUTSs)

Lower urinary tract symptoms (LUTSs) were assessed using a combination of validated standardized questionnaires, structured non-validated tools, and clinician-based diagnoses across the included studies. Validated instruments included the International Prostate Symptom Score (IPSS), International Consultation on Incontinence Questionnaire—Short Form (ICIQ-SF), Overactive Bladder Symptom Score (OABSS), Lower Urinary Tract Dysfunction Research Network Symptom Index-10 (LURN SI-10), and modular questionnaires such as the ICIQ-FLUTS (female version), ICIQ-MLUTS (male version), and ICIQ-OAB. The IPSS was used to assess the overall severity of LUTSs, with scores of ≥8 considered indicative of moderate-to-severe symptoms and used as a proxy for the presence of LUTSs. The ICIQ-SF was commonly used to assess urinary incontinence subtypes, including stress urinary incontinence (SUI), urgency urinary incontinence (UUI), and mixed urinary incontinence (MUI). The OABSS was used to identify overactive bladder, with a score of ≥6 indicating moderate-to-severe symptom severity. The ICIQ-FLUTS and ICIQ-MLUTS provided the modular assessments of LUTS domains including filling, voiding, and incontinence symptoms, while the ICIQ-OAB specifically addressed frequency, urgency, nocturia, and urgency incontinence. The LURN SI-10 was used as a concise, validated tool to evaluate a broad spectrum of LUTSs in both men and women.

Structured non-validated tools included self-reported symptom questionnaires and sleep diaries. Although these instruments had not undergone formal psychometric validation, they were included when their design, administration procedures, and scoring methods were clearly described and judged to be methodologically sound and reproducible. These tools were typically used to assess specific LUTS domains—such as nocturia, urinary hesitancy, incomplete emptying, and incontinence—in observational or epidemiological settings where standardized tools were unavailable or impractical. Their inclusion was intended to reflect the diversity of assessment practices in the literature and to avoid excluding potentially relevant studies that met all other inclusion criteria.

In some studies, LUTSs were identified through clinician diagnosis or extracted from medical records. These classifications were based on professional clinical assessment or diagnostic coding and did not rely on patient-reported instruments.

### 2.6. Data Extraction and Risk-of-Bias Assessment

Two reviewers (L.H. Tan and C.L. Chou) independently extracted data from each included study, collecting information on study design, population characteristics, diagnostic criteria, sample size, reported odds ratios, and prevalence rates. In instances where multiple studies analyzed overlapping populations, the study with the largest sample size or the most comprehensive dataset was selected. When odds ratios or prevalence estimates were not directly reported, they were calculated from the available raw data when possible.

The methodological quality of each study was assessed using the Joanna Briggs Institute (JBI) Critical Appraisal Checklist for Analytical Cross-Sectional Studies, covering eight domains as presented in Supplementary Figure S1. Each item was rated as “Yes”, “No”, or “Unclear.” Based on the number of affirmative (“Yes”) responses, studies were categorized as having low risk of bias (7–8 “Yes” responses), moderate risk or some concerns (5–6 “Yes”), or high risk of bias (≤4 “Yes”).

Risk-of-bias visualizations were generated using the robvis package in R (version 4.5.0) and RStudio (version 2025.05.0 + 496 for Windows). Graphical outputs included traffic light plots for individual studies (Supplementary Figure S1) and summary bar plots illustrating domain-level distributions across studies (Supplementary Figure S2).

### 2.7. Statistical Analysis

All statistical analyses were conducted using Comprehensive Meta-Analysis (CMA), Version 4 (Biostat, Englewood, NJ, USA). The primary meta-analysis estimated the pooled odds ratios (ORs) for lower urinary tract symptoms (LUTSs) in individuals with sarcopenia using a random-effects model, along with the pooled prevalence of LUTSs among individuals with sarcopenia. The random-effects model was chosen to account for between-study heterogeneity and to allow generalization beyond the included studies, under the assumption that the analyzed studies represent a random sample from a broader spectrum of possible studies. This approach is consistent with the best practices in recent meta-analyses in clinical epidemiology [29]. Secondary analyses assessed pooled ORs and prevalence estimates for related exposures, specifically the individual diagnostic components of sarcopenia—low muscle strength (LMS), low lean mass (LLM), and low gait speed (LGS)—as well as sarcopenia risk identified by the SARC-F questionnaire (score ≥ 4), in line with the screening recommendations from the AWGS and EWGSOP.

Subgroup analyses were conducted based on the diagnostic criteria for sarcopenia (AWGS vs. EWGSOP) and LUTS types (i.e., urinary incontinence vs. non-urinary incontinence). A mixed-effects model was applied for all subgroup analyses, using random-effects models within each subgroup and a fixed-effect model across subgroups to assess between-group differences. Additionally, a nested subgroup analysis was performed within studies using the EWGSOP criteria by adjusting the control group to compare individuals with severe sarcopenia versus those with non-severe sarcopenia.

Meta-regression analyses were conducted using a random-effects model, with log-transformed odds ratios (ORs) as the dependent variable. Study-level covariates included mean age, body mass index (BMI), sex distribution (categorized as male-only, female-only, or mixed populations), diagnostic criteria for sarcopenia, World Health Organization (WHO) regions, and risk-of-bias classification. The Knapp–Hartung adjustment was applied to improve the accuracy of standard error estimates and to provide more reliable confidence intervals, particularly in the presence of moderate between-study heterogeneity. Additionally, a world map depicting the pooled ORs and prevalence rates of sarcopenia associated with lower urinary tract symptoms (LUTSs) by WHO regions was generated using Plotly in Python (version 3.13.3).

Sensitivity analyses were conducted to assess the robustness of the findings by comparing cohort versus cross-sectional study designs, fixed-effects versus random-effects models, and leave-one-out analyses. Additionally, studies that used calf circumference to define low lean mass in accordance with AWGS criteria were excluded in a separate sensitivity analysis, to assess the impact of including surrogate measures of muscle mass.

Heterogeneity was quantified using the I^2^ statistic, with values greater than 50% considered indicative of substantial heterogeneity. Publication bias was evaluated through the visual inspection of funnel plots and assessed formally using Egger’s test, with a significance threshold of *p* < 0.10. Where asymmetry was detected, the trim-and-fill method was applied to estimate and adjust for potentially missing studies.

### 2.8. Post Hoc Subgroup and Meta-Regression Analyses of LLM Definitions

To explore the potential impact of heterogeneity in the definitions of low lean mass (LLM) across studies, we conducted post hoc subgroup and meta-regression analyses. These analyses were not pre-specified in the study protocol and were undertaken after the initial data synthesis to examine whether variations in diagnostic criteria influenced the strength of the association between LLM and lower urinary tract symptoms (LUTSs). Given the wide variability in LLM definitions across the included studies, this step was considered necessary to evaluate the robustness and consistency of the observed associations.

In the subgroup analyses, studies were categorized based on the type of diagnostic approach used to define LLM. These included (1) standardized criteria, such as appendicular skeletal muscle index (ASMI) or ASM adjusted for body mass index (ASM/BMI); (2) imaging-based methods, such as skeletal muscle index (SMI) or psoas muscle area (PMA); and (3) surrogate anthropometric measures, primarily calf circumference. Pooled effect estimates were computed for each category, and between-group comparisons were conducted to assess potential differences in effect size.

Furthermore, a univariate meta-regression analysis was performed using the LLM definition type as a categorical moderator variable. The goal was to determine whether the choice of diagnostic method significantly accounted for heterogeneity in the association between LLM and LUTSs. These analyses were considered exploratory in nature and were conducted to generate hypotheses regarding the role of measurement variability in shaping study outcomes.

### 2.9. Certainty Assessment

We assessed the certainty (confidence) in the body of evidence for each outcome using the GRADE (Grading of Recommendations Assessment, Development and Evaluation) approach. This involved evaluating five domains: risk of bias, inconsistency, indirectness, imprecision, and publication bias. Two reviewers (L.H. Tan and C.L. Chou) independently rated the certainty of evidence for the primary and secondary outcomes. Disagreements were resolved through discussion and consensus. The final certainty ratings were categorized as high, moderate, low, or very low, and summarized in accordance with the GRADE guidance.

## 3. Results

### 3.1. Overview of Enrolled Studies

**Figure 1:**
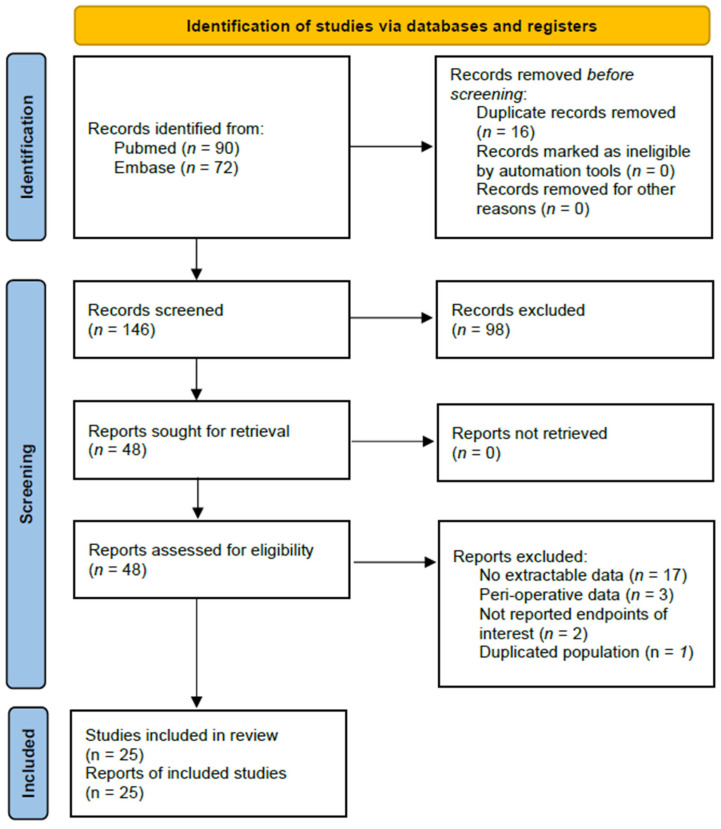
Flow chart for full-text screening.

A total of 162 articles were identified through searches in secondary databases, specifically PubMed and Embase (Figure 1). After the removal of 16 duplicate records, 146 studies underwent title and abstract screening. Of these, 98 were excluded for the following reasons: 64 were non-original studies (such as reviews, editorials, letters, or case reports), 30 did not report endpoints of interest, 3 were conference abstracts, and 1 involved a pediatric population. The remaining 48 articles were retrieved for full-text review. Following detailed assessment, 23 articles were excluded: 17 lacked extractable data, 3 focused on perioperative data that posed a high risk of confounding, 2 did not report relevant outcomes, and 1 involved a duplicated population.

Ultimately, 25 studies met the inclusion criteria and were included in the analysis, providing odds ratio and/or prevalence data on the association between sarcopenia and lower urinary tract symptoms (LUTSs), comprising a total of 84,484 participants. The pooled sample included 16,419 individuals with sarcopenia, 882 with low muscle strength (LMS), 14,835 with low lean mass (LLM), 361 with low gait speed (LGS), 104 identified as being at risk for sarcopenia based on a SARC-F score ≥ 4, and 53,311 control participants.

Among studies that reported demographic data, the pooled mean age of individuals with sarcopenia was 68.1 years (pooled SD: 10.5), and the pooled mean BMI was 22.0 kg/m^2^ (pooled SD: 5.1), indicating a primarily older, lean population.

A total of 12 studies on definitive sarcopenia were included in the primary meta-analysis. Secondary analyses were conducted for LMS (5 studies), LLM (13 studies), LGS (3 studies), and sarcopenia risk identified by SARC-F (≥4) (3 studies). The characteristics of the included studies are presented in Table 1 and Supplementary Tables S3–S6 [14,19,20,24,28,30,31,32,33,34,35,36,37,38,39,40,41,42,43,44,45,46,47,48,49,50].

### 3.2. Risk-of-Bias Assessment

Summaries of the risk of bias for the included studies are presented in Supplementary Figures S1 and S2. Among the 25 studies included in the analysis of lower urinary tract symptoms (LUTSs), 21 studies (84.0%) were assessed as having a low risk of bias, while 4 studies (16.0%) were judged to have some concerns or moderate risk.

### 3.3. Main Meta-Analysis

**Figure 2:**
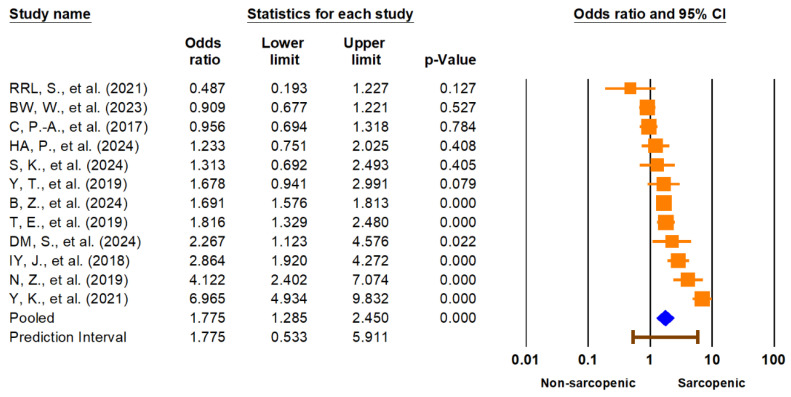
Forest plot showing the pooled odds ratio (OR) for the association between sarcopenia and lower urinary tract symptoms (LUTSs) across all included studies. Significant heterogeneity was observed (I^2^ = 90.9%, *p* < 0.001, Cochran’s Q test) [14,19,20,24,28,30,31,32,33,34,35,36,37,38,39,40,41,42,43,44,45,46,47,48,49,50]. Note: Orange squares represent individual study estimates, and blue diamonds represent the pooled summary effect.

**Figure 3:**
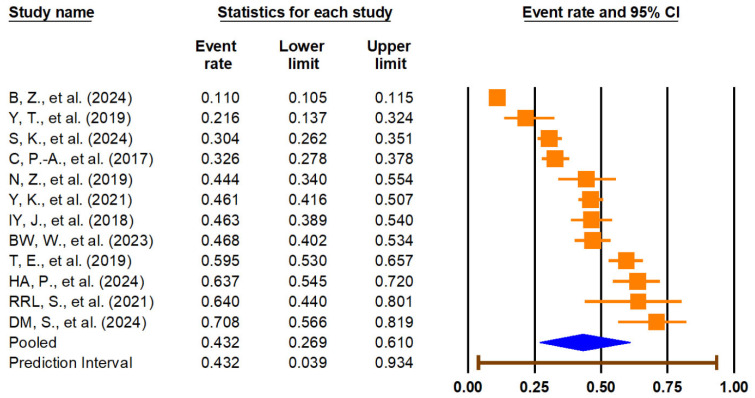
Forest plot showing the pooled prevalence of lower urinary tract symptoms (LUTSs) among individuals with sarcopenia across all included studies. Significant heterogeneity was observed (I^2^ = 99.2%, *p* < 0.001, Cochran’s Q test) [14,19,20,24,28,30,31,32,33,34,35,36,37,38,39,40,41,42,43,44,45,46,47,48,49,50]. Note: Orange squares represent individual study estimates, and blue diamonds represent the pooled summary effect.

The primary meta-analysis demonstrated a significant association between sarcopenia and lower urinary tract symptoms (LUTSs). The pooled odds ratio (OR) for LUTSs in individuals with sarcopenia compared to those without was 1.78 (95% CI: 1.29–2.45, *p* < 0.001) (Figure 2). The 95% prediction interval ranged from 0.53 to 5.91, suggesting substantial heterogeneity in true effects across different populations and settings. Heterogeneity was high (I^2^ = 90.9%). The pooled prevalence of LUTSs among individuals with sarcopenia was 43.2% (95% CI: 26.9–61.0%), with a wide prediction interval (3.9% to 93.4%) and similar high heterogeneity (I^2^ = 99.2%), reflecting significant variation across studies. These findings are illustrated in Figure 3. Given the extremely high I^2^ values and wide prediction intervals, these pooled estimates should be interpreted with caution. The observed heterogeneity likely reflects differences in study populations, diagnostic definitions, and methodological quality, which may limit the generalizability and certainty of the summary effect.

**Figure 4:**
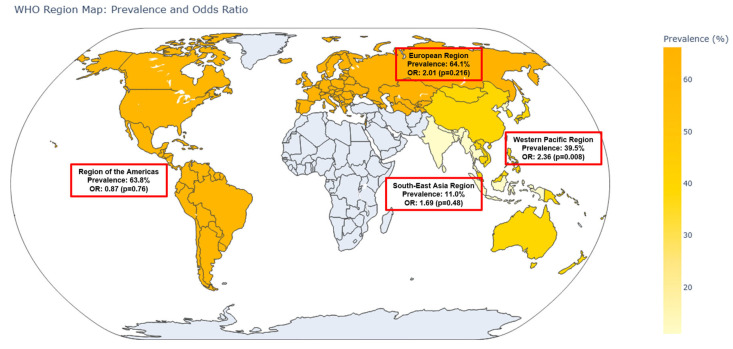
World map stratified by World Health Organization (WHO) regions, showing the pooled odds ratios (ORs) and pooled prevalence of lower urinary tract symptoms (LUTSs) among individuals with sarcopenia in each region. A yellow color scale was used to represent prevalence, with deeper shades indicating higher pooled prevalence. The map was generated using Plotly in Python (version 3.13.3).

When stratified by World Health Organization (WHO) regions, notable variations were observed. In the Western Pacific Region (n = 6 studies), the pooled OR was 2.36 (95% CI: 1.25–4.43, *p* = 0.008), with a corresponding prevalence of 39.5% (95% CI: 32.3–47.2%). The European Region (n = 2 studies) yielded an OR of 2.01 (95% CI: 0.67–6.08, *p* = 0.216) and the highest prevalence of 64.1% (95% CI: 50.0–76.1%). In the Region of the Americas (n = 3 studies), the pooled OR was 0.87 (95% CI: 0.35–2.17, *p* = 0.758) with a prevalence of 63.8% (95% CI: 48.3–76.9%). The Southeast Asia Region (n = 1 study) had an OR of 1.69 (95% CI: 0.39–7.39, *p* = 0.485) and a notably lower prevalence of 11.0% (95% CI: 5.8–19.8%). These regional differences are visualized in the world map (Figure 4).

**Figure 5:**
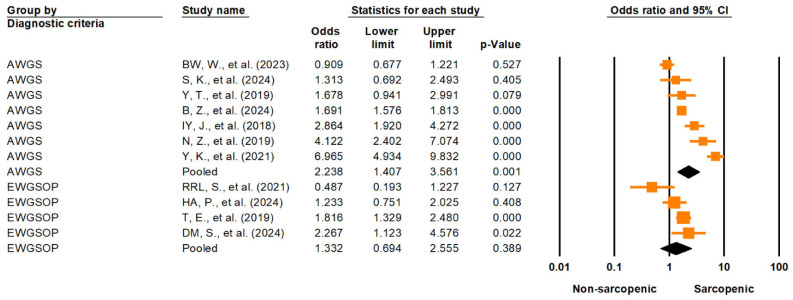
Forest plot showing the subgroup analysis comparing individuals with sarcopenia diagnosed using the AWGS versus EWGSOP criteria in relation to LUTSs. High heterogeneity was observed (I^2^ = 90.7%, df = 10, *p* < 0.001). The between-group difference was not statistically significant (Q = 1.618, df = 1, *p* = 0.203) [14,19,20,24,30,31,32,33,34,35,36,37,38,39,40,41,42,43,44,45,46,47,48,49,50]. Note: Orange squares represent individual study estimates, and black diamonds represent the pooled summary effect.

Subgroup analyses were performed to evaluate the impact of different diagnostic criteria and study-level factors on the association between sarcopenia and lower urinary tract symptoms (LUTSs). Studies using the Asian Working Group for Sarcopenia (AWGS) criteria showed a significantly higher pooled OR (2.24; 95% CI: 1.41–3.56; *p* = 0.001) than those using the European Working Group on Sarcopenia in Older People (EWGSOP) criteria (1.33; 95% CI: 0.69–2.56; *p* = 0.389) (Figure 5). However, the between-group difference was not statistically significant (Q = 1.62; df = 1; *p* = 0.203). A further comparison between the AWGS 2014 and AWGS 2019 revealed a pooled OR of 2.72 (95% CI: 1.22–6.05; *p* = 0.014) for the AWGS 2014 and 1.95 (95% CI: 0.998–3.82; *p* = 0.051) for the AWGS 2019, with no significant difference between the subgroups (Q = 0.387; df = 1; *p* = 0.534) (Supplementary Figure S3). Sensitivity analysis excluding the single study using the EWGSOP1 resulted in a pooled OR of 1.17 (95% CI: 0.56–2.44; *p* = 0.671), indicating no material change in effect size.

**Figure 6:**
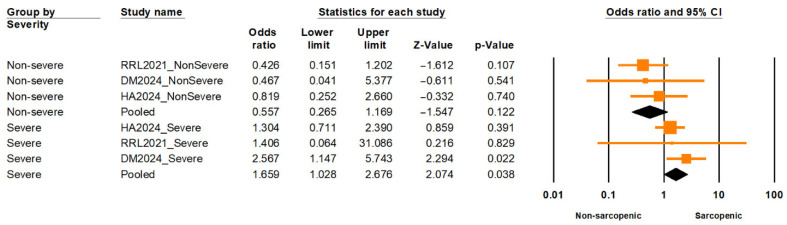
Forest plot showing the nested subgroup analysis comparing severe versus non-severe sarcopenia based on the EWGSOP criteria. Moderate heterogeneity was observed within subgroups (I^2^ = 39.8%, df = 5, *p* = 0.14), with a significant difference between subgroups (Q = 5.875, df = 1, *p* = 0.015) [14,19,20,24,30,31,32,33,34,35,36,37,38,39,40,41,42,43,44,45,46,47,48,49,50]. Note: Orange squares represent individual study estimates, and black diamonds represent the pooled summary effect.

A nested subgroup analysis comparing individuals with severe versus non-severe sarcopenia found a significantly elevated risk of LUTSs among those with severe sarcopenia (OR = 1.66; 95% CI: 1.03–2.68; *p* = 0.038), while non-severe sarcopenia was not significantly associated (OR = 0.56; 95% CI: 0.27–1.17; *p* = 0.122) (Figure 6). The difference between these subgroups was statistically significant (Q = 5.875; df = 1; *p* = 0.015), supporting a severity-dependent association. Moderate heterogeneity was observed within subgroups (I^2^ = 39.8%, df = 5; *p* = 0.14), based on a fixed-effect estimation.

**Figure 7:**
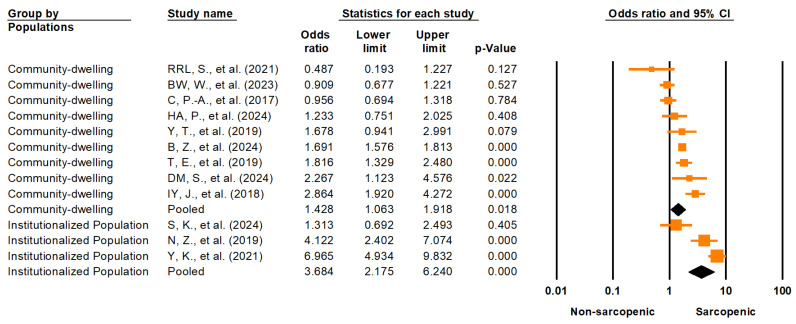
Forest plot showing the subgroup analysis comparing community-dwelling versus institutionalized populations. High heterogeneity was observed within subgroups (I^2^ = 90.21%, df = 11, *p* < 0.001), with a significant difference between subgroups (Q = 9.464, df = 1, *p* = 0.002) [14,19,20,24,28,30,31,32,33,34,35,36,37,38,39,40,41,42,43,44,45,46,47,48,49,50]. Note: Orange squares represent individual study estimates, and black diamonds represent the pooled summary effect.

A subgroup analysis based on population setting revealed a significantly higher pooled OR in institutionalized populations (OR = 3.68; 95% CI: 2.18–6.24; *p* < 0.001) compared to community-dwelling populations (OR = 1.43; 95% CI: 1.06–1.92; *p* = 0.018). The between-group difference was statistically significant (Q = 9.464; df = 1; *p* = 0.002), suggesting that institutionalization may modify the association between sarcopenia and LUTSs (Figure 7). We also performed a corresponding stratification for prevalence: the pooled prevalence of LUTSs among individuals with sarcopenia was 40.1% (95% CI: 12.6–75.6%) in institutionalized settings and 45.8% (95% CI: 24.7–68.6%) in community-dwelling populations (Supplementary Figure S22). However, the between-group difference was not statistically significant (Q = 0.065; df = 1; *p* = 0.799), indicating that population setting alone does not explain the wide prediction interval (3.3–94.8%) observed in pooled prevalence estimates.

Subgroup analysis based on LUTS subtype revealed a pooled OR of 1.69 (95% CI: 1.12–2.53; *p* = 0.012) for urinary incontinence (UI) and 1.97 (95% CI: 1.08–3.62; *p* = 0.028) for non-incontinence LUTSs (e.g., frequency, urgency, nocturia) (Supplementary Figure S4). The difference between these subgroups was not statistically significant (Q = 0.175; df = 1; *p* = 0.676).

Additionally, a subgroup analysis based on the type of LUTS assessment tool showed a significantly higher pooled OR in studies that used validated standardized questionnaires (2.48; 95% CI: 1.40–4.39; *p* = 0.002) compared to those using clinician-diagnosed or medical record-based classifications (1.39; 95% CI: 0.68–2.85; *p* = 0.370) and structured non-validated questionnaires or logs (1.37; 95% CI: 0.71–2.67; *p* = 0.351) (Supplementary Figure S5). However, the between-group difference did not reach statistical significance (Q = 2.326; df = 2; *p* = 0.313).

### 3.4. Meta-Regression Analysis

To explore the potential sources of heterogeneity in the association between sarcopenia and lower urinary tract symptoms (LUTSs), univariate random-effects meta-regression analyses were performed using log-transformed odds ratios (ORs) as the dependent variable. The moderator variables included mean age, mean body mass index (BMI), gender distribution (male-only, female-only, or both sexes), diagnostic criteria for sarcopenia (e.g., AWGS 2014/2019, EWGSOP1/2, or pooled standard cutoffs), type of LUTS assessment tool, World Health Organization (WHO) region, and risk of bias. The Knapp–Hartung method was applied to yield the robust estimates of standard errors and 95% confidence intervals.

As shown in Supplementary Table S7 and Supplementary Figure S6, none of the covariates were significantly associated with the effect size, and all models yielded R^2^ = 0, indicating no reduction in between-study heterogeneity. Specifically, neither mean age (β = 0.01; 95% CI: −0.03 to 0.05; *p* = 0.64) nor mean BMI (β = −0.08; 95% CI: −0.22 to 0.07; *p* = 0.26) significantly moderated the association. Similarly, gender distribution showed no significant effect, whether studies included female-only participants (β = −0.85; *p* = 0.15) or both sexes (β = −0.06; *p* = 0.91), compared to male-only studies.

Regarding sarcopenia diagnostic criteria, no significant differences were observed between the AWGS 2014 (reference) and AWGS 2019, EWGSOP1, or EWGSOP2. Interestingly, the use of three standard diagnostic cutoffs (ASMI, ASM/BMI, or calf circumference) approached statistical significance (β = −1.04; 95% CI: −2.02 to −0.05; *p* = 0.05), though it did not account for heterogeneity (R^2^ = 0).

The type of LUTS assessment tool was also not associated with the effect size. Compared to validated standardized questionnaires, studies using clinician-diagnosed or medical record-based classifications (β = 0.59; *p* = 0.24) and structured non-validated questionnaires or logs (β = 0.01; *p* = 0.98) showed no significant differences.

Analysis by WHO regions revealed no significant geographic effect. Compared to the European Region, studies conducted in the Americas (β = −0.84; *p* = 0.28), Southeast Asia (β = −0.17; *p* = 0.86), and Western Pacific (β = 0.16; *p* = 0.81) showed no significant differences. Additionally, risk of bias did not significantly influence the pooled ORs; studies with moderate risk showed no significant difference compared to those with low risk (β = 0.28; *p* = 0.64).

In summary, none of the assessed study-level characteristics significantly explained the heterogeneity in the association between sarcopenia and LUTSs. These findings suggest that the observed variability across studies may be driven by unmeasured or more complex interacting factors not captured in the current analysis.

### 3.5. Sensitivity Analysis and Publication Bias

Several sensitivity analyses were conducted to assess the robustness of the association between sarcopenia and lower urinary tract symptoms (LUTSs). The leave-one-out analysis, in which each study was sequentially excluded from the meta-analysis, yielded a pooled odds ratio (OR) of 1.78 (95% CI: 1.29–2.45, *p* < 0.001) (Supplementary Figure S7). This result was consistent with the main analysis, indicating that no single study unduly influenced the overall effect size.

When comparing the random-effects model with a fixed-effect model, the fixed-effect analysis produced a pooled OR of 1.71 (95% CI: 1.61–1.82, *p* < 0.001). The similar magnitude and significance of this estimate further support the stability of the findings.

A subgroup analysis based on study design was also performed. Among cohort studies (n = 2), the pooled OR was 2.57 (95% CI: 1.16–5.69, *p* = 0.021), indicating a stronger association compared to cross-sectional studies (n = 10), which showed a pooled OR of 1.63 (95% CI: 1.12–2.38, *p* = 0.011) (Supplementary Figure S8). However, the Q test revealed no statistically significant difference between the two groups (Q = 1.016, df = 1, *p* = 0.313).

Additionally, we conducted a sensitivity analysis excluding one study that diagnosed low lean mass (LLM) using calf circumference, a surrogate marker based on the Asian Working Group for Sarcopenia (AWGS) guidelines. The resulting pooled OR was 1.82 (95% CI: 1.29–2.55, *p* = 0.001), nearly identical to the main estimate, confirming that the observed association was robust to variations in sarcopenia definitions.

**Figure 8:**
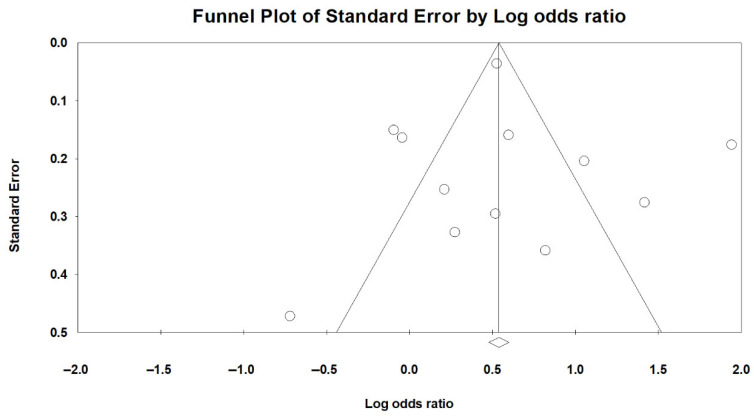
Funnel plot of the odds ratio for the association between sarcopenia and LUTSs. Circles represent individual studies; the vertical line indicates the overall pooled logit effect size. Diagonal lines represent the 95% pseudo-confidence limits. The diamond represents the adjusted effect size and confidence interval estimated using the trim-and-fill method.

Publication bias was evaluated using both visual inspection of the funnel plot (Figure 8) and Egger’s regression test. Egger’s test yielded an intercept of 0.29 (SE = 1.39; 95% CI: −2.80 to 3.38), with a t-value of 0.21 (df = 10) and a two-tailed *p*-value of 0.838, indicating no statistically significant funnel plot asymmetry. These findings suggest no evidence of small-study effects or substantial publication bias in the analysis of sarcopenia and LUTSs.

To further evaluate potential bias due to unpublished studies, a trim-and-fill analysis was conducted. The method did not impute any missing studies, and the adjusted pooled effect size remained unchanged from the original estimate. This finding reinforces the conclusion that publication bias is unlikely to have influenced the observed association between sarcopenia and LUTSs.

### 3.6. Secondary Meta-Analyses and Post Hoc Evaluation of LLM Definitions

In addition to the main analysis, secondary meta-analyses were conducted to evaluate associations between individual diagnostic components or screening tools of sarcopenia and lower urinary tract symptoms (LUTSs).

For low lean mass (LLM), the pooled odds ratio (OR) was 1.52 (95% CI: 1.19–1.95, *p* = 0.001), indicating a significant positive association (Supplementary Figure S9). The pooled prevalence of LUTSs in individuals with LLM was 36.5% (95% CI: 23.5–52.0%) (Supplementary Figure S10).

In contrast, low muscle strength (LMS) was not significantly associated with LUTSs (pooled OR = 0.94; 95% CI: 0.47–1.89; *p* = 0.871), though the pooled LUTS prevalence remained relatively high at 44.4% (95% CI: 29.2–60.7%) (Supplementary Figures S11 and S12).

For low gait speed (LGS), a significant association with LUTSs was found (OR = 1.37; 95% CI: 1.06–1.76; *p* = 0.015), and the pooled prevalence was 36.3% (95% CI: 26.7–47.1%) (Supplementary Figures S13 and S14).

Individuals identified as at-risk for sarcopenia based on a SARC-F score ≥4 exhibited the strongest association, with a pooled OR of 3.20 (95% CI: 1.92–5.33; *p* < 0.001), and a LUTS prevalence of 36.3% (95% CI: 26.7–47.1%) (Supplementary Figures S15 and S16).

Subgroup comparisons using mutually exclusive populations revealed no statistically significant differences between sarcopenia and LLM (Q = 0.052, df = 1, *p* = 0.82), or between sarcopenia and SARC-F–based risk (Q = 2.41, df = 1, *p* = 0.12) (Supplementary Figures S17 and S18), suggesting that although SARC-F yielded the highest point estimate, these differences were not statistically meaningful.

To address variability in LLM definitions, post hoc subgroup and meta-regression analyses were conducted.

Studies using the standardized LLM criteria (e.g., ASMI or ASM/BMI) demonstrated a significant association with LUTSs (pooled OR = 1.51; 95% CI: 1.07–2.13; *p* = 0.019), while imaging-based methods (e.g., skeletal muscle index [SMI] or psoas muscle area [PMA]) showed a non-significant OR of 1.44 (95% CI: 0.64–3.25; *p* = 0.376). However, the between-group difference was not statistically significant (Q = 0.01, df = 1, *p* = 0.92) (Supplementary Figure S19).

A second subgroup analysis compared standardized criteria (n = 9) with surrogate anthropometric measures (calf circumference; n = 2). Pooled ORs were 1.52 (95% CI: 1.05–2.20; *p* = 0.028) and 1.50 (95% CI: 0.74–3.04; *p* = 0.256), respectively, again without a significant between-group difference (Q = 0.00, df = 1, *p* = 0.984) (Supplementary Figure S20).

Meta-regression further showed that the LLM definition did not significantly moderate the effect size. Regression coefficients were non-significant for imaging-based (–0.0609; *p* = 0.917), non-standard (0.1163; *p* = 0.861), and surrogate measures (–0.0077; *p* = 0.988), with an intercept of 0.4164 (*p* = 0.098), indicating no moderating effect of LLM definition (Supplementary Figure S21).

Taken together, these findings indicate that, while numerically stronger associations were seen with the standardized definitions of LLM, differences in diagnostic methods did not significantly influence the magnitude or direction of observed associations with LUTSs.

### 3.7. Certainty of Evidence

The overall certainty of evidence for the association between sarcopenia and lower urinary tract symptoms (LUTSs) was considered moderate, based on the GRADE approach [51]. Although the meta-analysis demonstrated a statistically significant association (pooled OR: 1.78, 95% CI: 1.29–2.45, *p* < 0.001), several factors warrant cautious interpretation. A summary of the GRADE certainty ratings for the primary and secondary outcomes is presented in Supplementary Table S8.

Risk of bias was generally low, with 84.0% (21 of 25) of included studies judged to be at low risk, and the remaining 16.0% at moderate risk—raising only minor concerns. However, heterogeneity was substantial (I^2^ = 90.9% for odds ratios; I^2^ = 99.2% for prevalence estimates), indicating large variability in effect sizes that could not be explained by subgroup analyses or meta-regression. This inconsistency across studies was the main reason for downgrading the certainty rating.

Indirectness was judged to be minimal, as most studies directly evaluated the research question using recognized definitions for sarcopenia and clinically relevant LUTS outcomes. Imprecision was not a major concern, with confidence intervals sufficiently narrow to suggest meaningful associations. Publication bias was not detected based on Egger’s test (*p* = 0.838) and trim-and-fill analysis, which identified no missing studies.

In summary, although the evidence consistently supports a positive association between sarcopenia and LUTSs, the certainty of the findings was downgraded from high to moderate due to substantial heterogeneity and unexplained variability across studies.

## 4. Discussion

### 4.1. Summary of Main Findings

This systematic review and meta-analysis demonstrates a consistent and clinically meaningful association between sarcopenia and lower urinary tract symptoms (LUTSs). The pooled odds ratio (OR) for LUTSs in individuals with sarcopenia compared to controls was 1.78 (95% CI: 1.29–2.45, *p* < 0.001), indicating a significantly increased risk. However, the wide 95% prediction interval (0.53–5.91) and substantial heterogeneity (I^2^ = 90.9%) suggest that this association may vary considerably across populations and settings.

The pooled prevalence of LUTSs among individuals with sarcopenia was 43.2% (95% CI: 26.9–61.0%), accompanied by an extremely wide prediction interval (3.9% to 93.4%) and high heterogeneity (I^2^ = 99.2%). These results highlight the substantial burden of LUTSs in sarcopenic populations and reveal variability across studies, likely influenced by methodological, demographic, or clinical differences.

Collectively, the findings support the hypothesis that sarcopenia—characterized by deficits in muscle mass, strength, and physical performance—is associated with the elevated risk and burden of LUTSs. However, the degree of variability observed emphasizes the importance of context-specific screening and intervention strategies, as well as the potential influence of broader physiological and environmental factors.

### 4.2. Regional Variations in Association

Subgroup analysis by World Health Organization (WHO) regions revealed notable geographic variability in both the strength of association and the prevalence of lower urinary tract symptoms (LUTSs) among individuals with sarcopenia. For example, in the Western Pacific Region (including Taiwan, Japan, and China), the association between sarcopenia and LUTSs was statistically significant (OR: 2.36; 95% CI: 1.25–4.43), and the prevalence of LUTSs in sarcopenic individuals was moderate (39.5%). In contrast, the European Region exhibited a high LUTS prevalence (64.1%) but failed to reach statistical significance in the odds ratio (OR: 2.01; 95% CI: 0.67–6.08), likely due to limited study numbers and reduced precision. The Region of the Americas showed a null association (OR: 0.87; 95% CI: 0.35–2.17), despite a comparably high LUTS prevalence (63.8%). Such disparities warrant careful interpretation and contextualization.

Several factors may explain these regional differences. First, variations in demographic characteristics—such as age distribution, body composition, life expectancy, and baseline health status [24]—can influence the strength of the observed association between sarcopenia and LUTSs. For instance, populations with higher rates of obesity, diabetes, or sedentary lifestyle may exhibit more pronounced musculoskeletal and urologic decline, intensifying the interrelationship between these conditions.

Second, differences in diagnostic standards and operational definitions may introduce substantial heterogeneity [52]. The Western Pacific studies primarily used the AWGS criteria, which are tailored to Asian populations, while European studies favored the EWGSOP criteria, which have stricter thresholds and a stepwise staging model. Such definitional inconsistencies may alter case classification, leading to differential misclassification bias across regions. Similarly, LUTS definitions varied: while some studies used validated instruments (e.g., IPSS, OABSS), others relied on self-report or sleep diaries, which can under- or overestimate true symptom burden depending on the cultural context and participant literacy [53].

Third, healthcare system characteristics and sociocultural norms likely play a critical role [12]. In countries with strong primary care infrastructure and proactive geriatric screening (e.g., Japan or South Korea), sarcopenia and LUTSs may be more consistently recognized and documented. Conversely, in under-resourced settings or those with high stigma around urinary symptoms—especially among women or institutionalized individuals—underreporting and underdiagnosis may attenuate observed associations. Cross-national differences in physical activity, nutritional intake, and attitudes toward aging and continence further complicate interpretation.

Finally, methodological limitations, including small sample sizes in certain regions, cross-sectional designs, and inconsistent covariate adjustment, may reduce statistical power and obscure true associations. The non-significant findings in Europe and the Americas may therefore reflect imprecision rather than the absence of effect.

However, formal meta-regression using WHO regions as a moderator did not identify statistically significant differences in effect size across regions, suggesting that the observed geographic patterns may stem from sampling variability and study-level factors rather than true regional disparities.

In sum, the observed regional heterogeneity calls for the cautious interpretation of pooled estimates. The association between sarcopenia and LUTSs is not uniform but appears to be contextually influenced by demographic, clinical, methodological, and sociocultural factors. These findings emphasize the need for region-specific studies using harmonized diagnostic criteria, larger sample sizes, and standardized outcome assessments to enhance comparability and guide locally relevant clinical interventions [54].

### 4.3. Impact of Diagnostic Criteria and Sarcopenia Severity

The observed associations between sarcopenia and lower urinary tract symptoms (LUTSs) differed according to the diagnostic criteria used. Studies that employed the Asian Working Group for Sarcopenia (AWGS) definitions demonstrated a significantly elevated pooled odds ratio (OR = 2.24, 95% CI: 1.41–3.56), whereas those using the European Working Group on Sarcopenia in Older People (EWGSOP) criteria yielded a lower and statistically non-significant pooled OR (1.33, 95% CI: 0.69–2.56). Although the between-group heterogeneity was not statistically significant (Q = 1.62, *p* = 0.203), the marked difference in effect estimates warrants clinical and methodological considerations.

Several factors may contribute to this discrepancy. First, the AWGS was specifically developed for Asian populations, incorporating regionally calibrated cutoff values for muscle mass, strength, and physical performance. These cutoffs are typically lower than those in the EWGSOP and are more sensitive to detecting early or subtle sarcopenia phenotypes in Asian cohorts. As a result, the AWGS-defined sarcopenia may capture a broader or more clinically relevant spectrum of muscle decline linked to LUTSs in these populations.

Second, the greater OR in the AWGS-defined studies may reflect population-related confounders. Most included studies using the AWGS were conducted in East Asian countries, where lifestyle factors, body composition patterns, and sarcopenia phenotypes may differ from Western populations studied under the EWGSOP. These contextual differences could amplify the observed association between sarcopenia and LUTSs, even if the formal subgroup comparison was not statistically significant due to limited power or sample imbalance.

Furthermore, the sensitivity analysis excluding the single study based on the EWGSOP1 did not materially alter the effect size, suggesting that the lack of significance in the EWGSOP group is unlikely to be attributable to legacy diagnostic versions alone, but rather a combination of diagnostic stringency and population heterogeneity.

When comparing the AWGS 2014 and AWGS 2019, the AWGS 2014 showed a statistically significant association with LUTSs (OR = 2.72, 95% CI: 1.22–6.05), whereas the AWGS 2019 did not reach statistical significance (OR = 1.95, 95% CI: 0.998–3.82, *p* = 0.051). This difference may reflect key updates in the AWGS 2019 criteria, which introduced more permissive thresholds (e.g., gait speed < 1.0 m/s vs. <0.8 m/s in AWGS 2014) and adopted a two-step diagnostic algorithm that begins with the SARC-F screening followed by confirmatory assessments. Although SARC-F showed a strong association with LUTSs in our study, the structured diagnostic flow may have reduced overall sensitivity—particularly in population-based settings—by excluding individuals who failed to meet subjective screening thresholds despite having objectively low muscle strength or mass. Moreover, variability in how studies applied the two-step process (e.g., whether only confirmed sarcopenia was analyzed) could have introduced diagnostic heterogeneity and weakened the observed associations. Nevertheless, the lack of a statistically significant difference between the subgroups (Q = 0.387, *p* = 0.534) suggests that the observed divergence in pooled odds ratios may be attributable to random variation rather than a true difference in diagnostic validity.

In addition, the borderline *p*-value for the AWGS 2019 (*p* = 0.051) suggests that the lack of statistical significance is more likely due to a limited sample size or wider confidence intervals, rather than a true absence of association. While the AWGS 2019 aimed to improve the clinical utility and consistency of sarcopenia diagnosis, its more structured diagnostic framework may have inadvertently reduced sensitivity in broader population settings—particularly when individuals with relevant impairments were not captured by the initial SARC-F screen. As a result, the true strength of the association between sarcopenia and LUTSs may have been underestimated.

Taken together, these findings emphasize how diagnostic methodology strongly influences observed associations in sarcopenia research. Although statistical comparisons between subgroups did not reach significance, the consistently stronger associations observed with the AWGS—particularly the AWGS 2014—highlight the importance of regional calibration, diagnostic thresholds, and guideline implementation. These methodological factors are essential for accurately estimating sarcopenia-related risks.

Conversely, individuals classified as having severe sarcopenia exhibited a significantly higher risk of LUTSs compared to those with non-severe forms. This subgroup difference reached statistical significance, supporting a dose–response relationship between sarcopenia severity and LUTS risk. Importantly, heterogeneity within subgroups was moderate, suggesting a reasonably consistent effect across studies. A similar trend has been observed in other systemic outcomes. For example, a population-based study by Kim, B., et al. (2024) demonstrated that arterial stiffness—as measured by estimated pulse wave velocity—and both systolic and diastolic blood pressure showed a stepwise increase across sarcopenia severity, from normal to moderate-to-severe stages [55]. These findings reinforce the biological plausibility of the association, as more advanced muscle loss may lead to impaired pelvic floor and postural control, which are crucial for urinary continence [56]. They also highlight the importance of stratifying sarcopenia severity in clinical assessments of older adults with urinary symptoms to inform prognosis and individualized interventions [54].

### 4.4. Contextual Differences: Community-Dwelling vs. Institutionalized Populations

Our findings suggest that the association between sarcopenia and lower urinary tract symptoms (LUTSs) may be modified by the care setting. Specifically, studies conducted in institutionalized populations yielded a significantly stronger pooled odds ratio (OR = 3.68; 95% CI: 2.18–6.24; *p* < 0.001) than those in community-dwelling populations (OR = 1.43; 95% CI: 1.06–1.92; *p* = 0.018), with a statistically significant between-group difference (Q = 9.464; df = 1; *p* = 0.002). This suggests that institutionalized individuals may represent a higher-risk subgroup in whom sarcopenia has a more pronounced impact on LUTSs.

The exaggerated effect observed in institutionalized settings may reflect several interrelated factors. Residents of long-term care facilities or post-acute wards are more likely to exhibit advanced frailty, dependency for activities of daily living, polypharmacy, and comorbidities such as dementia or neurological conditions—all of which are independently associated with both sarcopenia and LUTSs [57,58]. Furthermore, limited mobility and reduced access to exercise or rehabilitation in such environments may exacerbate muscular atrophy and pelvic floor dysfunction [59]. Bladder dysfunction may also be underrecognized or underreported in these populations due to cognitive impairment or care prioritization, potentially compounding the severity of LUTSs [60].

However, when stratifying by care setting, the pooled prevalence of LUTSs among individuals with sarcopenia did not significantly differ between institutionalized (40.1%; 95% CI: 12.6–75.6%) and community-dwelling populations (45.8%; 95% CI: 24.7–68.6%), with no statistically significant between-group difference (Q = 0.065; df = 1; *p* = 0.799). This finding illustrates the extreme heterogeneity in prevalence estimates, as reflected by the wide 95% prediction interval observed across studies (3.3% to 94.8%). These results indicate that, while a setting may influence the strength of the association between sarcopenia and LUTSs, it does not fully account for the variability in symptom burden. Other unmeasured moderators, such as hydration status, diuretic use, psychosocial stressors, and institutional care quality, may contribute to this heterogeneity [61].

Given these findings, future studies should consider stratification by living environment and include the standardized assessments of frailty, functional status, and care quality when evaluating the sarcopenia–LUTS relationship. This could improve the external validity and clinical applicability of pooled estimates across diverse geriatric populations.

### 4.5. Impact of LUTS Assessment Tools

The method used to assess lower urinary tract symptoms (LUTSs) appeared to influence the strength and statistical significance of the association with sarcopenia. In subgroup analysis, studies that used clinician-based diagnosis or medical record abstraction demonstrated a significant association (pooled OR: 2.48; 95% CI: 1.40–4.39; *p* = 0.002), while those using validated standardized questionnaires (pooled OR: 1.39; *p* = 0.370) or structured non-validated questionnaires or logs (pooled OR: 1.37; *p* = 0.351) did not reach statistical significance. However, the overall difference between these subgroups was not statistically significant (Q = 2.326; *p* = 0.313), suggesting that observed differences may reflect variability in study design, population characteristics, or clinical context rather than a true differential effect.

The stronger association observed in studies relying on clinician diagnosis or medical record abstraction may reflect several factors. These studies are often conducted in hospital-based or high-risk populations, where both sarcopenia and LUTSs may present more severely and are more likely to be documented due to clinical relevance [12,62]. Additionally, clinician-based classifications may prioritize patients with overt or bothersome symptoms, leading to the enrichment of cases with clinically significant LUTSs. This reduces the risk of outcome misclassification and can inflate the observed strength of association [63].

In contrast, studies using validated questionnaires—while methodologically rigorous—may include broader, population-based samples and capture a wider spectrum of symptom severity, including mild or subclinical cases. This could attenuate the observed association if milder LUTSs are less strongly linked to sarcopenia. Supporting this notion, Surmeli, D. M., et al. (2024) observed a higher prevalence of sarcopenia among individuals with moderate-to-severe LUTS; however, the association did not reach statistical significance after adjusting for confounders (OR: 2.04; 95% CI: 0.94–4.45; *p* = 0.070), suggesting that the relationship may be more detectable in settings enriched for more severe LUTS presentations [14]. Moreover, variability in scoring thresholds, symptom domains, and recall periods across validated tools such as the IPSS, ICIQ, or OAB-q may lead to inconsistent case classification, particularly when used across different populations or cultural contexts [63,64,65,66].

Similarly, structured non-validated tools, although practical, may lack formal reliability testing and are prone to recall or interpretation bias, especially when based on self-report without standardization or clinician confirmation [67]. These limitations are particularly relevant in older populations, where cognitive impairment or underreporting of urinary symptoms is common. For example, recent studies have shown that overactive bladder is associated with reduced cognitive performance in older adults, with depressive symptoms mediating this relationship and potentially affecting symptom reporting [68]. Additionally, urinary dysfunction often precedes cognitive symptoms in patients with vascular cognitive impairment, yet may remain underreported due to communication or motor deficits [60].

Together, these findings suggest that the clinical context and case selection—in addition to instrument validity—play critical roles in shaping the observed associations. While validated tools remain essential for standardization and reproducibility in epidemiologic research, clinician-based assessments may more accurately capture severe and clinically actionable LUTSs, thereby yielding stronger associations with sarcopenia.

### 4.6. Meta-Regression Interpretation

Despite substantial between-study heterogeneity, none of the examined study-level characteristics significantly explained the variability in effect sizes. Meta-regression analyses incorporating covariates such as mean age, BMI, gender distribution, sarcopenia diagnostic criteria, LUTS assessment tools, WHO region, and risk of bias yielded non-significant results, with all models producing R^2^ = 0.

Interestingly, the use of the three standardized diagnostic cutoffs for sarcopenia approached statistical significance (β = −1.04; 95% CI: −2.02 to −0.05; *p* = 0.05), suggesting a potential trend toward lower effect sizes in these studies, although it did not account for the observed heterogeneity. Likewise, the type of LUTS assessment tool showed no significant effect in meta-regression despite its apparent influence in subgroup analysis. This discrepancy may reflect limited power within subgroups or residual confounding.

The inability of meta-regression to identify significant moderators underscores the complexity of the sarcopenia–LUTS relationship and suggests that heterogeneity may be driven by unmeasured variables such as comorbidities, physical activity levels, hormonal status, or contextual factors like care setting or diagnostic intensity. These findings highlight the limitations of study-level meta-regression in uncovering nuanced interactions and the potential value of individual participant data (IPD) meta-analyses in future work, which can account for within-study variability and allow for a more precise modeling of effect modifiers [69].

### 4.7. Component-Level Associations

In component-specific analyses, both low lean mass (LLM) and low gait speed (LGS) were significantly associated with lower urinary tract symptoms (LUTSs), whereas low muscle strength (LMS) was not. Notably, all included LMS studies assessed muscle strength using handgrip dynamometry, a measure primarily reflecting upper-body strength. This finding challenges the assumption that handgrip strength alone adequately captures the muscle function relevant to urinary control.

The discrepancy may stem from the distinct physiological roles of various muscle groups. LLM and LGS are indicative of lower-body and overall functional performance, which are crucial for pelvic floor stability, postural alignment, and neuromuscular coordination—all integral to urinary continence mechanisms. In contrast, handgrip strength does not directly assess the strength of muscles involved in pelvic floor function.

Emerging evidence suggests that assessments targeting lower-body and pelvic musculature may provide more accurate insights into the relationship between muscle strength and LUTSs. For instance, a study from the Baltimore Longitudinal Study of Aging demonstrated that thigh muscle strength, measured via knee extensor torque, was inversely associated with LUTS severity in older men [21]. Additionally, tools such as perineometers, electromyography (EMG), and ultrasound imaging have been utilized to assess pelvic floor muscle (PFM) strength, offering the direct evaluation of the muscles responsible for urinary control [70].

These findings advocate for a multi-dimensional approach to sarcopenia assessment in LUTS research and clinical practice. Emphasizing evaluations of lower-body strength, pelvic floor function, and physical performance may yield more accurate associations with LUTSs than relying solely on handgrip strength. Incorporating diverse muscle strength assessments can enhance our understanding of the pathophysiological links between sarcopenia and urinary dysfunction.

### 4.8. Interpretation of Mutually Exclusive Subgroup Analysis

To isolate the influence of diagnostic approach on the strength of association between sarcopenia and LUTSs, subgroup analyses were conducted using mutually exclusive populations. These analyses revealed no statistically significant differences in effect sizes when comparing sarcopenia with either low lean mass (LLM)- or SARC-F-defined risk of sarcopenia. Although the SARC-F subgroup yielded the highest pooled odds ratio, this difference did not reach statistical significance relative to definitive sarcopenia (Q = 2.41, *p* = 0.12). Similarly, no significant difference was observed between the LLM and sarcopenia subgroups (Q = 0.052, *p* = 0.82). These results suggest that, despite variations in the diagnostic constructs and sensitivity of each measure, the overall strength of association with LUTSs remains broadly comparable across definitions. It is possible that underlying pathophysiological mechanisms linking muscle health and urinary symptoms are captured—albeit differently—by these diagnostic approaches. Moreover, the lack of statistical difference may reflect limitations in power due to small sample sizes within subgroups or overlapping constructs across diagnostic definitions. Future studies with harmonized case definitions and larger mutually exclusive cohorts are needed to clarify the comparative predictive value of each approach.

### 4.9. SARC-F and Functional Risk: Exploratory Insights

Notably, sarcopenia risk identified via the SARC-F questionnaire (score ≥4) demonstrated the strongest association with lower urinary tract symptoms (LUTSs) (OR = 3.20; 95% CI: 1.92–5.33; *p* < 0.001), exceeding the strength of associations observed for other sarcopenia components. The SARC-F tool, which captures self-reported limitations in strength, walking ability, rising from a chair, climbing stairs, and falls, may reflect broader neuromuscular impairment or frailty rather than sarcopenia alone [71].

Interestingly, two out of the three studies using SARC-F focused on overactive bladder (OAB), while the majority of definitive sarcopenia studies assessed urinary incontinence (UI), and none specifically evaluated OAB. Although no pooled estimate could be generated for OAB among definitive sarcopenia studies, this pattern raises the hypothesis that sarcopenia—particularly in functionally impaired or frail individuals—may be more strongly associated with OAB than with other LUTS subtypes.

This hypothesis is biologically plausible. OAB is associated with detrusor overactivity, impaired central inhibition, and altered bladder afferent signaling [72]. Sarcopenia may exacerbate these mechanisms via autonomic dysfunction, pelvic floor instability, and reduced mobility, all of which are also captured in functional screening tools like SARC-F. Furthermore, frailty—a condition strongly linked to sarcopenia—has been independently associated with diminished bladder function, including urgency and voiding difficulty [73].

From a clinical standpoint, SARC-F offers practical advantages: it is quick, non-invasive, equipment-free, and feasible for screening in both outpatient and community settings [74]. However, its emphasis on sensitivity over specificity means it may overidentify at-risk individuals, some of whom may not meet formal diagnostic thresholds for sarcopenia [75]. A two-step screening approach—initial SARC-F followed by objective confirmation (e.g., grip strength, gait speed, or DXA)—may improve diagnostic precision and reduce false positives [76].

Together, these exploratory findings support the utility of functional screening tools like SARC-F for identifying individuals at high risk for LUTSs, particularly OAB, and highlight the need for future studies to evaluate this link using standardized definitions and symptom-specific endpoints.

### 4.10. Role of Diagnostic Methodology in LLM

Post hoc subgroup and meta-regression analyses were conducted to explore whether variability in the diagnostic methods used to define low lean mass (LLM) influenced the strength of association with lower urinary tract symptoms (LUTSs). Studies employing standardized definitions—such as ASMI or ASM/BMI based on the AWGS or EWGSOP criteria—showed statistically significant associations with LUTSs. In contrast, studies using imaging-based indices (e.g., skeletal muscle index [SMI], psoas muscle area [PMA]) or surrogate anthropometric measures (e.g., calf circumference) reported weaker and generally non-significant associations.

These trends likely reflect underlying differences in what each measurement method captures. DXA-derived metrics such as ASMI or ASM/BMI are validated indicators of limb-specific muscle mass and correlate well with physical function and clinical outcomes [77]. Imaging-based methods, by contrast, often quantify isolated muscle groups that may not directly reflect whole-body muscle status or functional capacity, and may have limited relevance to pelvic support or urological outcomes [78,79]. Similarly, calf circumference—while practical and low-cost—can be influenced by subcutaneous fat, edema, or body habitus, particularly in elderly or obese populations, potentially reducing its specificity and reliability as a proxy for lean mass [80].

However, despite these observed trends, neither subgroup comparisons nor meta-regression analyses yielded statistically significant between-group differences. This may be attributable to small sample sizes within subgroups, methodological heterogeneity, and the inconsistent application of diagnostic thresholds across studies. These limitations point to the urgent need for harmonized, clinically meaningful criteria to define LLM in both research and practice, thereby improving comparability and interpretability of findings.

### 4.11. Strengths and Limitations

This systematic review and meta-analysis synthesized data from 25 observational studies involving over 84,000 participants across multiple WHO regions, providing a geographically diverse evidence base. The study adhered to rigorous methodological standards, including PROSPERO pre-registration and full compliance with PRISMA and MOOSE guidelines. In addition to evaluating the overall association between sarcopenia and lower urinary tract symptoms (LUTSs), we examined associations by sarcopenia components, LUTS subtypes, severity classifications, diagnostic modalities, and study setting (community-dwelling vs. institutionalized populations), thereby enabling an exploration of contextual influences on the observed relationships. The robustness of findings was supported by multiple sensitivity analyses—including leave-one-out procedures and comparison of fixed- and random-effects models—with no evidence of publication bias detected.

Nonetheless, several limitations must be acknowledged. First, substantial heterogeneity was present across most analyses (I^2^ > 90%), likely arising from variations in study populations, sarcopenia definitions, LUTS measurement tools, and study designs. To explore sources of heterogeneity, we conducted univariate meta-regression using study-level covariates such as mean age, BMI, gender distribution, sarcopenia definition, LUTS subtype, WHO region, and risk-of-bias classification. None of these factors significantly explained the observed heterogeneity (R^2^ = 0). Although the use of standardized diagnostic criteria approached significance (*p* = 0.05), it did not account for the variability. This lack of explanatory power may reflect limitations in the granularity and consistency of the primary studies rather than flaws in model specification. Key variables such as frailty status, hormonal profiles, physical function, and comorbidity burden were often unreported or inconsistently defined, limiting our ability to build more refined models. As such, our findings regarding heterogeneity should be interpreted with caution as a data-driven constraint.

The high I^2^ values and wide 95% prediction intervals suggest meaningful variation in true effect sizes across different populations and settings. Therefore, although the pooled estimates offer useful insights into overall trends, they should not be assumed to represent universally applicable magnitudes of effect.

Second, all included studies were observational, and the majority were cross-sectional in design. This limits the ability to draw causal inferences or establish temporal sequences between sarcopenia and LUTSs. Residual confounding remains a major concern, especially for covariates such as physical activity, medication use (e.g., diuretics, anticholinergics, alpha-blockers), cognitive status, and comorbid conditions (e.g., cardiovascular disease, diabetes, neurological disorders), which were not uniformly reported or adjusted for. The absence of such adjustments limits the ability to isolate the independent effect of sarcopenia on LUTSs and raises the possibility of spurious or attenuated associations. Future studies using individual-level data with rigorous confounder control are warranted.

Third, most odds ratios were calculated from raw event counts reported in the included studies. In a few instances where adjusted estimates were available but raw data were not, we reconstructed event tables to estimate crude ORs. Thus, our pooled results reflect a combination of crude and reconstructed ORs, which may introduce further variability and should be interpreted accordingly.

Fourth, while we applied no language restrictions, several non-English articles were translated using automated tools such as Google Translate and ChatGPT. Although we exercised caution in interpretation, minor inaccuracies may have occurred.

Finally, although our search strategy followed a systematic approach, it was limited to two databases (PubMed and Embase) due to institutional access restrictions. These databases are widely recognized for their comprehensive coverage of biomedical literature. To mitigate the risk of missing relevant studies, we also manually reviewed reference lists of included articles and related reviews. Nonetheless, the omission of additional databases (e.g., Web of Science, Scopus, Cochrane Library) represents a potential source of selection bias.

Based on the GRADE framework, the overall certainty of evidence was rated as moderate. This rating reflects the consistency in the direction of associations, biological plausibility of the underlying mechanisms, and the absence of publication bias. However, confidence in the pooled effect sizes is tempered by the observational nature of the included studies, high heterogeneity, and the use of aggregate data that limited adjustment for key confounders. To enhance causal inference and guide clinical decision making, future research should prioritize prospective longitudinal studies employing standardized diagnostic criteria, individual-level data, and comprehensive covariate control.

### 4.12. Potential Bidirectional Mechanisms Linking Sarcopenia and LUTSs

In addition to sarcopenia predisposing individuals to lower urinary tract symptoms (LUTSs), a plausible bidirectional relationship should be considered. For instance, nocturia—a common and burdensome LUTSs—can significantly disrupt sleep architecture, leading to fragmented sleep and daytime fatigue [81]. Chronic sleep disruption has been associated with reduced physical activity, hormonal dysregulation (e.g., decreased testosterone and growth hormone), and impaired muscle protein synthesis, which are key contributors to the development or progression of sarcopenia [82,83,84]. Moreover, LUTS-related embarrassment or fear of leakage may cause older adults to avoid physical activity and social engagement, leading to deconditioning and muscle atrophy [85]. Conversely, sarcopenia can impair pelvic floor function, gait, and mobility, contributing to incomplete bladder emptying or reduced toileting independence, further exacerbating LUTSs [47]. These interconnected pathways suggest a potential vicious cycle between sarcopenia and LUTS, reinforcing the need for integrative assessment and management strategies that address both conditions concurrently.

### 4.13. Implications for Clinical Practice and Future Research

This meta-analysis highlights a significant and clinically relevant association between sarcopenia and lower urinary tract symptoms (LUTSs), particularly in high-risk populations such as institutionalized older adults. Although these findings do not establish causality, they support the clinical relevance of assessing sarcopenia in older patients with LUTSs, particularly those with frailty, mobility issues, or poor response to standard treatments.

Two clinical hypotheses emerge from our findings. First, the significant associations observed for low lean mass (LLM) and low gait speed (LGS) suggest that interventions aimed at increasing muscle mass and enhancing physical performance—such as resistance training, nutritional support, and physical therapy—may help reduce LUTS burden. While promising, this hypothesis requires prospective validation in interventional studies focused on functional restoration and musculoskeletal health.

Second, routine screening with brief tools like the SARC-F questionnaire may provide a pragmatic approach for identifying individuals at-risk of sarcopenia in primary care or geriatric urology settings. Beyond screening, the SARC-F scores could potentially serve as a clinical indicator for monitoring treatment response. Although speculative, improvement in the SARC-F scores following muscle-targeted interventions may correlate with LUTS improvement. Future longitudinal studies should examine whether temporal changes in the SARC-F scores predict meaningful clinical changes in LUTS severity.

To advance this field, future research should prioritize longitudinal and interventional designs that clarify the directionality and underlying mechanisms of the sarcopenia–LUTS relationship, including pelvic floor function, neuromuscular control, and systemic inflammatory or hormonal pathways. Additionally, harmonizing diagnostic criteria for both sarcopenia and LUTSs will enhance data comparability and support the development of integrated care models tailored to aging populations.

## 5. Conclusions

This study provides evidence that sarcopenia is significantly associated with lower urinary tract symptoms (LUTSs), with a pooled odds ratio of 1.78 and an overall LUTS prevalence of 43.2% among individuals with sarcopenia. Notably, stronger associations were observed in individuals with more severe forms of sarcopenia and in institutionalized populations. Additionally, individuals who screened positive for sarcopenia using the SARC-F tool also demonstrated an elevated risk of LUTSs, underscoring the potential clinical utility of simple screening instruments. While these findings highlight the importance of addressing muscle health in older adults with LUTSs, causal relationships remain unconfirmed. Therefore, routine screening and intervention should be guided by further longitudinal and interventional research. These results support the need for integrated geriatric–urologic care and further exploration of muscle-targeted strategies to improve urinary and functional outcomes in aging populations.

## Figures and Tables

**Table 1:** Characteristics of studies included in the main meta-analysis of the association between sarcopenia and LUTSs.

Author (year)	Study Design	Study Design	Gender	Mean Age (years) ± SD	Mean BMI (kg/m^2^) ± SD	Country	WHO Regions	Sarcopenic Sample Size (*n*)	Control Sample size (*n*)	Reported LUTSs	LUTS Cases in Sarcopenic Group (*n*)	LUTS Cases in Control Group (*n*)	Diagnostic Criteria for Sarcopenia	LUTS Assessment Tools
B, Z., et al. (2024) [36]	Cross-sectional	Community-dwelling	M and F	67.5 ± 9.6	21.7 ± 4.9	India	Southeast Asia Region	14,282	28,068	SUI	1567	1907	AWGS 2019	Structured non-validated questionnaires or logs: self-reported questionnaire
BW, W., et al. (2023) [41]	Cross-sectional	Community-dwelling	F	57.9 ± 6.3	NA	Singapore	Western Pacific Region	216	985	UI	101	484	AWGS 2019	Validated standardized questionnaire: PFDI
C, P.-A., et al. (2017) [28]	Prospective cohort	Community-dwelling	F	74.6 ± 2.9	28.3 ± 5.7	USA	Region of the Americas	334	339	UI	109	114	3 standard cutoffs *	Structured non-validated questionnaires or logs: self-reported questionnaire
DM, S., et al. (2024) [14]	Cross-sectional	Community-dwelling	M	71.3 ± 8.2	27.7 ± 3.9	Turkey	European Region	48	145	IPSS-defined LUTSs	34	75	EWGSOP2	Validated standardized questionnaire: IPSS ≥ 8
HA, P., et al. (2024) [34]	Cross-sectional	Community-dwelling	M and F	87.3 ± 5.2	25.7 ± 5.3	Brazil	Region of the Americas	113	160	UI	72	94	EWGSOP2	Clinician-diagnosed or medical record-based classification
IY, J., et al. (2018) [49]	Cross-sectional	Community-dwelling	M	74.2 ± 5.6	NA	Korea	Western Pacific Region	164	328	IPSS-defined LUTSs	76	76	AWGS 2014	Validated standardized questionnaire: IPSS ≥ 8
N, Z., et al. (2019) [50]	Cross-sectional	Institutionalized Population	M and F	78.6 ± 6.0	23.5 ± 4.2	China	Western Pacific Region	81	283	UI	36	46	AWGS 2014	Clinician-diagnosed or medical record-based classification
RRL, S., et al. (2021) [43]	Cross-sectional	Community-dwelling	F	69.5 ± 6.7	28.3 ± 4.7	Brazil	Region of the Americas	25	121	UI	16	95	EWGSOP2	Validated standardized questionnaire: ICIQ-SF
										SUI	6	13		
										UUI	4	24		
										MUI	6	58		
S, K., et al. (2024) [33]	Cross-sectional	Institutionalized Population	M and F	83.0 ± 8.2	19.6 ± 3.5	Japan	Western Pacific Region	404	56	UI	50	4	AWGS 2019	Clinician-diagnosed or medical record-based classification
										VD	123	14		
T, E., et al. (2019) [47]	Cross-sectional	Community-dwelling	F	47.0 ± 11.9	30.6 ± 5.5	Turkey	European Region	227	575	UI	135	257	EWGSOP1	Clinician-diagnosed or medical record-based classification
Y, K., et al. (2021) [42]	Retrospective cohort	Institutionalized Population	M and F	74.7 ± 13.5	21.5 ± 3.8	Japan	Western Pacific Region	451	466	UI	208	51	AWGS 2019	Clinician-diagnosed or medical record-based classification
Y, T., et al. (2019) [46]	Cross-sectional	Community-dwelling	F	67.4 ± 5.2	22.1 ± 3.1	Japan	Western Pacific Region	74	1133	Nocturia	16	160	AWGS 2014	Structured non-validated questionnaires or logs: sleep diary

Abbreviations: LUTSs, lower urinary tract symptoms; SD, standard deviation; WHO, World Health Organization; BMI, body mass index; CI, confidence interval; NA, not applicable; USA, United State of America; M, male; F, female; M and F, male and female; UI, urinary incontinence; SUI, stress urinary incontinence; UUI, urgency urinary incontinence; MUI, mixed urinary incontinence; VD, voiding dysfunction; AWGS, Asian Working Group for Sarcopenia; EWGSOP, European Working Group on Sarcopenia in Older People; PFDI, Pelvic Floor Disability Index; IPSS, International Prostate Symptom Score; ICIQ-SF, International Consultation on Incontinence Questionnaire—Short Form. * “3 standard cutoffs” refers to a comprehensive definition of sarcopenia, which includes low muscle mass (ASMI < 5.5 kg/m^2^), low physical performance (gait speed < 1.0 m/s), and low muscle strength (grip strength < 20.5 kg).

## Supplementary Materials

The following supporting information can be downloaded at: https://www.mdpi.com/article/10.3390/medicina61071214/s1, Supplementary Table S1. Search strategy used in PubMed/MEDLINE. Supplementary Table S2. Search strategy used in Embase. Supplementary Table S3. Characteristics of studies included in the secondary meta-analysis on LMS and LUTSs. Supplementary Table S4. Characteristics of studies included in the secondary meta-analysis on LLM and LUTSs. Supplementary Table S5. Characteristics of studies included in the secondary meta-analysis on LGS and LUTSs. Supplementary Table S6. Characteristics of studies included in the secondary meta-analysis on sarcopenia risk and LUTSs. Supplementary Table S7. Univariate random-effects meta-regression of the association between sarcopenia and LUTSs. Supplementary Table S8. Summary of certainty of evidence using the GRADE framework. Supplementary Table S9. Summary of diagnostic criteria for sarcopenia according to AWGS and EWGSOP guidelines Supplementary Figure S1. Traffic light plot showing the risk-of-bias assessment across eight domains based on the JBI critical appraisal checklist for analytical cross-sectional studies. Supplementary Figure S2. Summary plot of the risk-of-bias assessment based on the JBI critical appraisal checklist for analytical cross-sectional studies. The plot displays the proportion of studies rated as “Yes” (low risk of bias) or “Unclear” (moderate risk of bias) across each of the eight domains. Supplementary Figure S3. Forest plot showing the subgroup analysis comparing individuals with sarcopenia diagnosed using AWGS 2014 versus AWGS 2019 criteria in relation to LUTSs. High heterogeneity was observed (I^2^ = 93.6%, df = 6, *p* < 0.001), and the between-group difference was not statistically significant (Q = 0.387, df = 1, *p* = 0.534). Supplementary Figure S4. Forest plot showing the subgroup analysis comparing individuals with sarcopenia who had urinary incontinence (UI) versus those without UI. High heterogeneity was observed (I^2^ = 90.9%, df = 11, *p* < 0.001), and the between-group difference was not statistically significant (Q = 0.175, df = 1, *p* = 0.676). Supplementary Figure S5. Forest plot showing the subgroup analysis comparing individuals with sarcopenia based on the method used to diagnose LUTSs: clinician-diagnosed or medical record–based classification, structured non-validated questionnaires or logs, and validated standardized questionnaires. High heterogeneity was observed (I^2^ = 90.9%, df = 11, *p* < 0.001), and the between-group difference was not statistically significant (Q = 2.326, df = 2, *p* = 0.313). Supplementary Figure S6. Univariate random-effects meta-regression analyses of the odds ratio (OR) for the association between sarcopenia and lower urinary tract symptoms (LUTSs) according to (a) mean age, (b) mean BMI, (c) gender distribution, (d) diagnostic criteria for sarcopenia, (e) LUTS assessment tools, (f) WHO region, and (g) risk of bias. Note: Circles represent individual studies, with size proportional to study weight. The central line indicates the fitted meta-regression line, and the outer lines represent the 95% confidence interval. Supplementary Figure S7. Forest plot showing the results of the leave-one-out sensitivity analysis assessing the robustness of the pooled odds ratio for the association between sarcopenia and LUTSs. Supplementary Figure S8. Forest plot showing the subgroup analysis comparing study design (cohort vs. cross-sectional) in studies evaluating the association between sarcopenia and LUTSs. High heterogeneity was observed (I^2^ = 90.9%, df = 11, *p* < 0.001), and the between-group difference was not statistically significant (Q = 1.016, df = 1, *p* = 0.313). Supplementary Figure S9. Forest plot showing the pooled odds ratio (OR) for the association between low lean mass (LLM) and lower urinary tract symptoms (LUTSs) across all included studies. Significant heterogeneity was observed (I^2^ = 88.1%, *p* < 0.001; Cochran’s Q test). Supplementary Figure S10. Forest plot showing the pooled prevalence of lower urinary tract symptoms (LUTSs) among individuals with LLM across all included studies. Significant heterogeneity was observed (I^2^ = 99.3%, *p* < 0.001, Cochran’s Q test). Supplementary Figure S11. Forest plot showing the pooled odds ratio (OR) for the association between low muscle strength (LMS) and lower urinary tract symptoms (LUTSs) across all included studies. Significant heterogeneity was observed (I^2^ = 93.4%, *p* < 0.001; Cochran’s Q test). Supplementary Figure S12. Forest plot showing the pooled prevalence of lower urinary tract symptoms (LUTSs) among individuals with low muscle strength (LMS) across all included studies. Significant heterogeneity was observed (I^2^ = 94.9%, *p* < 0.001; Cochran’s Q test). Supplementary Figure S13. Forest plot showing the pooled odds ratio (OR) for the association between low gait speed (LGS) and lower urinary tract symptoms (LUTSs) across all included studies. No significant heterogeneity was observed (I^2^ = 0.0%, *p* = 0.46; Cochran’s Q test). Supplementary Figure S14. Forest plot showing the pooled prevalence of lower urinary tract symptoms (LUTSs) among individuals with low gait speed (LGS) across all included studies. Significant heterogeneity was observed (I^2^ = 66.8%, *p* = 0.049; Cochran’s Q test). Supplementary Figure S15. Forest plot showing the pooled odds ratio (OR) for the association between risk of sarcopenia and lower urinary tract symptoms (LUTSs) across all included studies. No significant heterogeneity was observed (I^2^ = 0.0%, *p* = 0.612; Cochran’s Q test). Supplementary Figure S16. Forest plot showing the pooled prevalence of lower urinary tract symptoms (LUTSs) among individuals at-risk of sarcopenia across all included studies. Significant heterogeneity was observed (I^2^ = 88.0%, *p* < 0.001; Cochran’s Q test). Supplementary Figure S17. Forest plot showing the subgroup analysis comparing individuals with sarcopenia versus those with low lean mass (LLM) in relation to lower urinary tract symptoms (LUTSs), using mutually exclusive populations. High heterogeneity was observed (I^2^ = 89.6%, df = 19, *p* < 0.001), and the between-group difference was not statistically significant (Q = 0.052, df = 1, *p* = 0.82). Supplementary Figure S18. Forest plot showing the subgroup analysis comparing individuals with sarcopenia versus those with risk of sarcopenia in relation to lower urinary tract symptoms (LUTSs), using mutually exclusive populations. High heterogeneity was observed (I^2^ = 89.0%, df = 14, *p* < 0.001), and the between-group difference was not statistically significant (Q = 2.412, df = 1, *p* = 0.12). Supplementary Figure S19. Forest plot showing the subgroup analysis comparing individuals with low lean mass (LLM) defined by standardized diagnostic criteria versus imaging-based methods in relation to lower urinary tract symptoms (LUTSs). High heterogeneity was observed (I^2^ = 88.1%, df = 9, *p* < 0.001), and the between-group difference was not statistically significant (Q = 0.01, df = 1, *p* = 0.92). Supplementary Figure S20. Forest plot showing the subgroup analysis comparing individuals with low lean mass (LLM) defined by standardized diagnostic criteria versus a surrogate measure (calf circumference) in relation to lower urinary tract symptoms (LUTSs). High heterogeneity was observed (I^2^ = 90.8%, df = 9, *p* < 0.001), and the between-group difference was not statistically significant (Q = 0.00, df = 1, *p* = 0.984). Supplementary Figure S21. Univariate random-effects meta-regression analyses of the odds ratios (ORs) for the association between low lean mass (LLM) and lower urinary tract symptoms (LUTSs) across different definitions: standardized diagnostic criteria (SDCs), imaging-based methods (IBMs), non-standardized diagnostic criteria (Non-SDCs), and surrogate measures (SMs). Note: Circles represent individual studies, with size proportional to study weight. The central line indicates the fitted meta-regression line, and the outer lines represent the 95% confidence interval. Supplementary Figure S22. Forest plot showing the subgroup analysis of pooled prevalence of lower urinary tract symptoms in community-dwelling versus institutionalized populations. Substantial heterogeneity was observed within subgroups (I^2^ = 99.2%, *p* < 0.001, Cochran’s Q test), with no significant difference between subgroups (Q = 0.065, df = 1, *p* = 0.799).
