# Supplementary material for: Association and Prevalence of Lower Urinary Tract Symptoms in Individuals with Sarcopenia: A Systematic Review and Meta-Analysis
Source: Medicina (Kaunas). 2025 Jul 3;61(7):1214. doi: 10.3390/medicina61071214 (PMC12298974; doi:10.3390/medicina61071214)
Supplement: Supplementary file 1 [file medicina-61-01214-s001.zip › Supplementary Material.pdf]

Supplementary Materials

List of Supplementary Figures and Tables

1. **Supplementary Table S1.** Search strategy used in PubMed/MEDLINE.
2. **Supplementary Table S2.** Search strategy used in Embase.
3. **Supplementary Table S3.** Characteristics of Studies Included in the Secondary Meta-Analysis on LMS and LUTS.
4. **Supplementary Table S4.** Characteristics of Studies Included in the Secondary Meta-Analysis on LLM and LUTS.
5. **Supplementary Table S5.** Characteristics of Studies Included in the Secondary Meta-Analysis on LGS and LUTS.
6. **Supplementary Table S6.** Characteristics of Studies Included in the Secondary Meta-Analysis on sarcopenia risk and LUTS.
7. **Supplementary Table S7.** Univariate Random-Effects Meta-Regression of the Association Between Sarcopenia and LUTS.
8. **Supplementary Table S8.** Summary of Certainty of Evidence Using the GRADE Framework.
9. **Supplementary Table S9.** Summary of Diagnostic Criteria for Sarcopenia According to AWGS and EWGSOP Guidelines
10. **Supplementary Figure S1.** Traffic light plot showing the risk of bias assessment across eight domains based on the JBI critical appraisal checklist for analytical cross-sectional studies.
11. **Supplementary Figure S2.** Summary plot of the risk of bias assessment based on the JBI critical appraisal checklist for analytical cross-sectional studies. The plot displays the proportion of studies rated as "Yes" (low risk of bias) or "Unclear" (moderate risk of bias) across each of the eight domains.
12. **Supplementary Figure S3.** Forest plot showing the subgroup analysis comparing individuals with sarcopenia diagnosed using AWGS 2014 versus AWGS 2019 criteria in relation to LUTS. High heterogeneity was observed ($I^2 = 93.6\%$, df = 6, $p < 0.001$), and the between-group difference was not statistically significant (Q = 0.387, df = 1, p = 0.534).
13. **Supplementary Figure S4.** Forest plot showing the subgroup analysis comparing individuals with sarcopenia who had urinary incontinence (UI) versus those without UI. High heterogeneity was observed ($I^2 = 90.9\%$, df = 11, $p < 0.001$), and the between-group difference was not statistically significant (Q = 0.175, df = 1, p = 0.676).
14. **Supplementary Figure S5.** Forest plot showing the subgroup analysis comparing individuals with sarcopenia based on the method used to diagnose LUTS: clinician-diagnosed or medical record–based classification, structured non-validated questionnaires or logs, and validated standardized

questionnaires. High heterogeneity was observed ($I^2 = 90.9\%$, df = 11, $p < 0.001$), and the between-group difference was not statistically significant (Q = 2.326, df = 2, p = 0.313).

15. **Supplementary Figure S6.** Univariate random-effects meta-regression analyses of the odds ratio (OR) for the association between sarcopenia and lower urinary tract symptoms (LUTS) according to: (a) mean age, (b) mean BMI, (c) gender distribution, (d) diagnostic criteria for sarcopenia, (e) LUTS assessment tools, (f) WHO region, and (g) risk of bias. Note: Circles represent individual studies, with size proportional to study weight. The central line indicates the fitted meta-regression line, and the outer lines represent the 95% confidence interval.
16. **Supplementary Figure S7.** Forest plot showing the results of the leave-one-out sensitivity analysis assessing the robustness of the pooled odds ratio for the association between sarcopenia and LUTS.
17. **Supplementary Figure S8.** Forest plot showing the subgroup analysis comparing study design (cohort vs. cross-sectional) in studies evaluating the association between sarcopenia and LUTS. High heterogeneity was observed ($I^2 = 90.9\%$, df = 11, $p < 0.001$), and the between-group difference was not statistically significant (Q = 1.016, df = 1, p = 0.313).
18. **Supplementary Figure S9.** Forest plot showing the pooled odds ratio (OR) for the association between low lean mass (LLM) and lower urinary tract symptoms (LUTS) across all included studies. Significant heterogeneity was observed ($I^2 = 88.1\%$, $p < 0.001$; Cochran's Q test).
19. **Supplementary Figure S10.** Forest plot showing the pooled prevalence of lower urinary tract symptoms (LUTS) among individuals with LLM across all included studies. Significant heterogeneity was observed ($I^2 = 99.3\%$, $p < 0.001$, Cochran's Q test).
20. **Supplementary Figure S11.** Forest plot showing the pooled odds ratio (OR) for the association between low muscle strength (LMS) and lower urinary tract symptoms (LUTS) across all included studies. Significant heterogeneity was observed ($I^2 = 93.4\%$, $p < 0.001$; Cochran's Q test).
21. **Supplementary Figure S12.** Forest plot showing the pooled prevalence of lower urinary tract symptoms (LUTS) among individuals with low muscle strength (LMS) across all included studies. Significant heterogeneity was observed ($I^2 = 94.9\%$, $p < 0.001$; Cochran's Q test).
22. **Supplementary Figure S13.** Forest plot showing the pooled odds ratio (OR) for the association between low gait speed (LGS) and lower urinary tract symptoms (LUTS) across all included studies. No significant heterogeneity was observed ($I^2 = 0.0\%$, p = 0.46; Cochran's Q test).
23. **Supplementary Figure S14.** Forest plot showing the pooled prevalence of lower urinary tract symptoms (LUTS) among individuals with low gait speed (LGS) across all included studies. Significant heterogeneity was observed ($I^2 = 66.8\%$, p = 0.049; Cochran's Q test).
24. **Supplementary Figure S15.** Forest plot showing the pooled odds ratio (OR) for the association between risk of sarcopenia and lower urinary tract symptoms (LUTS) across all included studies. No significant heterogeneity was observed ($I^2 = 0.0\%$, p = 0.612; Cochran's Q test).

25. **Supplementary Figure S16.** Forest plot showing the pooled prevalence of lower urinary tract symptoms (LUTS) among individuals at risk of sarcopenia across all included studies. Significant heterogeneity was observed ($I^2 = 88.0\%$, $p < 0.001$; Cochran's Q test).
26. **Supplementary Figure S17.** Forest plot showing the subgroup analysis comparing individuals with sarcopenia versus those with low lean mass (LLM) in relation to lower urinary tract symptoms (LUTS), using mutually exclusive populations. High heterogeneity was observed ($I^2 = 89.6\%$, df = 19, $p < 0.001$), and the between-group difference was not statistically significant (Q = 0.052, df = 1, p = 0.82).
27. **Supplementary Figure S18.** Forest plot showing the subgroup analysis comparing individuals with sarcopenia versus those with risk of sarcopenia in relation to lower urinary tract symptoms (LUTS), using mutually exclusive populations. High heterogeneity was observed ($I^2 = 89.0\%$, df = 14, $p < 0.001$), and the between-group difference was not statistically significant (Q = 2.412, df = 1, p = 0.12).
28. **Supplementary Figure S19.** Forest plot showing the subgroup analysis comparing individuals with low lean mass (LLM) defined by standardized diagnostic criteria versus imaging-based methods in relation to lower urinary tract symptoms (LUTS). High heterogeneity was observed ($I^2 = 88.1\%$, df = 9, $p < 0.001$), and the between-group difference was not statistically significant (Q = 0.01, df = 1, p = 0.92).
29. **Supplementary Figure S20.** Forest plot showing the subgroup analysis comparing individuals with low lean mass (LLM) defined by standardized diagnostic criteria versus a surrogate measure (calf circumference) in relation to lower urinary tract symptoms (LUTS). High heterogeneity was observed ($I^2 = 90.8\%$, df = 9, $p < 0.001$), and the between-group difference was not statistically significant (Q = 0.00, df = 1, p = 0.984).
30. **Supplementary Figure S21.** Univariate random-effects meta-regression analyses of the odds ratios (ORs) for the association between low lean mass (LLM) and lower urinary tract symptoms (LUTS) across different definitions: standardized diagnostic criteria (SDC), imaging-based methods (IBM), non-standardized diagnostic criteria (Non-SDC), and surrogate measures (SM). Note: Circles represent individual studies, with size proportional to study weight. The central line indicates the fitted meta-regression line, and the outer lines represent the 95% confidence interval.
31. **Supplementary Figure S22.** Forest plot showing the subgroup analysis of pooled prevalence of lower urinary tract symptoms in community-dwelling versus institutionalized populations. Substantial heterogeneity was observed within subgroups ($I^2 = 99.2\%$, $p < 0.001$, Cochran's Q test), with no significant difference between subgroups (Q = 0.065, df = 1, p = 0.799).

Supplementary Table S1. Search strategy used in PubMed/MEDLINE.

**Concept #1. Sarcopenia 50194**
"Sarcopenia"[MeSH Terms] OR "Muscular Atrophy"[MeSH Terms] OR "sarcopenia*"[Title/Abstract] OR "muscular atroph*"[Title/Abstract] OR "neurogenic muscular atroph*"[Title/Abstract] OR "low lean mass"[Title/Abstract] OR "low muscle mass"[Title/Abstract] OR "muscle wasting"[Title/Abstract] OR "age-related muscle loss"[Title/Abstract]
**Concept #2. Lower Urinary Tract Symptoms 86518**
"Lower Urinary Tract Symptoms"[MeSH Terms] OR "Dysuria"[MeSH Terms] OR "Urinary Retention"[MeSH Terms] OR "Nocturia"[MeSH Terms] OR "Urinary Incontinence"[MeSH Terms] OR "urinary bladder, underactive"[MeSH Terms] OR "lower urinary tract symptom*"[Title/Abstract] OR "LUTS"[Title/Abstract] OR "Dysuria"[Title/Abstract] OR "urination disorder*"[Title/Abstract] OR "Urinary Retention"[Title/Abstract] OR "Nocturia"[Title/Abstract] OR "nycturia"[Title/Abstract] OR "overactive bladder*"[Title/Abstract] OR "underactive bladder*"[Title/Abstract] OR "overactive detrusor*"[Title/Abstract] OR "underactive detrusor*"[Title/Abstract] OR "hypotonic bladder*"[Title/Abstract] OR "Urinary Incontinence"[Title/Abstract] OR "stress incontinence"[Title/Abstract] OR "urge incontinence"[Title/Abstract]
**Concept #3: Restrict to human study 10096768**
("animals"[MeSH Terms] NOT ("humans"[MeSH Terms] AND "animals"[MeSH Terms])) OR ("plant s"[All Fields] OR "planted"[All Fields] OR "planting"[All Fields] OR "plantings"[All Fields] OR "plants"[MeSH Terms] OR "plants"[All Fields] OR "plant"[All Fields]) OR ("spacecraft"[MeSH Terms] OR "spacecraft"[All Fields] OR "spacecrafts"[All Fields] OR "spacecraft s"[All Fields]) OR "worm*"[All Fields] OR ("phonon s"[All Fields] OR "phononic"[All Fields] OR "phononics"[All Fields] OR "phonons"[MeSH Terms] OR "phonons"[All Fields] OR "phonon"[All Fields]) OR "worm"[All Fields] OR "rat"[All Fields] OR ("mice"[MeSH Terms] OR "mice"[All Fields]) OR ("rodent s"[All Fields] OR "rodentia"[MeSH Terms] OR "rodentia"[All Fields] OR "rodent"[All Fields] OR "rodents"[All Fields]) OR ("mice"[MeSH Terms] OR "mice"[All Fields] OR "mouse"[All Fields] OR "mouse s"[All Fields] OR "mouses"[All Fields]) OR ("dogs"[MeSH Terms] OR "dogs"[All Fields] OR "dog"[All Fields]) OR "cat"[All Fields] OR ("bovin"[All Fields] OR "cattle"[MeSH Terms] OR "cattle"[All Fields] OR

"bovine"[All Fields] OR "bovines"[All Fields]) OR ("plant s"[All Fields] OR "planted"[All Fields] OR "planting"[All Fields] OR "plantings"[All Fields] OR "plants"[MeSH Terms] OR "plants"[All Fields] OR "plant"[All Fields]) OR ("microorganism"[All Fields] OR "microorganism s"[All Fields] OR "microorganisms"[All Fields]) OR ("helminths"[MeSH Terms] OR "helminths"[All Fields] OR "worm"[All Fields]) OR ("military personnel"[MeSH Terms] OR ("military"[All Fields] AND "personnel"[All Fields]) OR "military personnel"[All Fields] OR "marine"[All Fields] OR "marines"[All Fields]) OR "mammal*"[All Fields] OR ("ocean"[All Fields] OR "ocean s"[All Fields] OR "oceanic"[All Fields] OR "oceans and seas"[MeSH Terms] OR ("oceans"[All Fields] AND "seas"[All Fields]) OR "oceans and seas"[All Fields] OR "oceans"[All Fields]) OR ("wavelength"[All Fields] OR "wavelengths"[All Fields]) OR ("spacecraft"[MeSH Terms] OR "spacecraft"[All Fields] OR "spacecrafts"[All Fields] OR "spacecraft s"[All Fields]) OR "voltammeter*"[All Fields] OR ("phonon s"[All Fields] OR "phononic"[All Fields] OR "phononics"[All Fields] OR "phonons"[MeSH Terms] OR "phonons"[All Fields] OR "phonon"[All Fields]) OR "worm*"[All Fields] OR "worm"[All Fields] OR ("ferrosoferric oxide"[Supplementary Concept] OR "ferrosoferric oxide"[All Fields] OR "magnetite"[All Fields] OR "ferrosoferric oxide"[MeSH Terms] OR ("ferrosoferric"[All Fields] AND "oxide"[All Fields]) OR "magnetites"[All Fields]) OR ("base sequence"[MeSH Terms] OR ("base"[All Fields] AND "sequence"[All Fields]) OR "base sequence"[All Fields] OR "sequence"[All Fields] OR "sequences"[All Fields] OR "sequence analysis"[MeSH Terms] OR ("sequence"[All Fields] AND "analysis"[All Fields]) OR "sequence analysis"[All Fields] OR "sequencing"[All Fields] OR "sequence s"[All Fields] OR "sequenceable"[All Fields] OR "sequenced"[All Fields] OR "sequenceing"[All Fields] OR "sequencer"[All Fields] OR "sequencers"[All Fields] OR "sequencies"[All Fields] OR "sequencings"[All Fields])

**Concept #4: Time 25135549**
("2000/01/01"[PDat]: "2025/04/26"[PDat])

**Strategy 90**
(#1 AND #2 AND #4) NOT #3

Supplementary Table S2. Search strategy used in Embase.

**Concept #1. Sarcopenia 35534**
('sarcopenia'/exp OR 'muscular atrophy'/exp OR sarcopenia*:ti,ab OR muscular atroph*:ti,ab OR neurogenic muscular atroph*:ti,ab OR "low lean mass":ti,ab OR "low muscle mass":ti,ab OR "muscle wasting":ti,ab OR "age-related muscle loss":ti,ab)
**Concept #2. Lower Urinary Tract Symptoms 194434**
('lower urinary tract symptoms'/exp OR 'dysuria'/exp OR 'urinary retention'/exp OR 'nocturia'/exp OR 'urinary incontinence'/exp OR 'overactive bladder'/exp OR 'underactive bladder'/exp OR "lower urinary tract symptom*":ti,ab OR LUTS:ti,ab OR dysuria:ti,ab OR "urination disorder*":ti,ab OR "urinary retention":ti,ab OR nocturia:ti,ab OR nycturia:ti,ab OR "overactive bladder*":ti,ab OR "underactive bladder*":ti,ab OR "overactive detrusor*":ti,ab OR "underactive detrusor*":ti,ab OR "hypotonic bladder*":ti,ab OR "urinary incontinence":ti,ab OR "stress incontinence":ti,ab OR "urge incontinence":ti,ab)
**Concept #3: Restrict to human study, Clinical Study and EMBASE results 1292657**
[humans]/lim AND [clinical study]/lim AND [embase]/lim
**Concept #4: Time 32017498**
[1-1-2000]/sd NOT [27-4-2025]/sd AND [2000-2025]/py
**Strategy 72**
#1 AND #2 AND #3 AND #4

Supplementary Table S3. Characteristics of Studies Included in the Secondary Meta-Analysis on LMS and LUTS.

Author (year)	Study Design	Gender	Mean Age (years) ± SD	Mean BMI (kg/m^2) ± SD	Country	WHO Regions	LMS Sample Size (n)	Control Sample Size (n)	LUTS reported	LUTS Cases in LMS Group (n)	LUTS Cases in Control Group (n)	LMS Criteria	LUTS Assessment Tools
T, E., et al. (2019)	Cross-sectional	F	73.8 ± 7.1	30.6 ± 5.5	Turkey	European Region	322	482	UI	178	214	Handgrip strength <22 kg	Clinician-diagnosed or medical record-based classification
Y, T., et al. (2019)	Cross-sectional	F	67.4 ± 5.2	22.1 ± 3.1	Japan	Western Pacific Region	145	1062	Nocturia	32	144	Handgrip strength <18 kg	Structured non-validated questionnaires or logs: *Sleep diary*
C, P.-A., et al. (2017)	Prospective cohort	F	74.6 ± 2.9	28.3 ± 5.7	USA	Region of the Americas	123	536	UI	41	359	Handgrip strength <20.5 kg	Structured non-validated questionnaires or logs: *Self-reported questionnaire*
RRL, S., et al. (2021)	Cross-sectional	F	69.5 ± 6.7	28.3 ± 4.7	Brazil	Region of the Americas	71	121	UI<br>SUI<br>UUI<br>MUI	55<br>5<br>17<br>33	95<br>13<br>24<br>58	Handgrip strength <16 kg	Validated standardized questionnaire: *ICIQ-SF*
SR, B., et al. (2024)	Prospective cohort	M&F	75.8 ± 4.5	27.6 ± 4.4	USA	Region of the Americas	221	415	LURN SI-10-defined LUTS	82	142	Handgrip strength <20 kg	Validated standardized questionnaire: *LURN SI-10*

Abbreviations: LMS, Low muscle strength; LUTS, Lower urinary tract symptoms; SD, Standard deviation; WHO, World Health Organization; BMI, Body Mass Index; CI, Confidence interval; USA, United State of America; F, Female; M&F, Male and female; UI, Urinary incontinence; SUI, Stress urinary incontinence; UUI, Urgency urinary incontinence; MUI, Mixed urinary incontinence; LURN SI-10, Lower Urinary Tract Dysfunction Research Network Symptom Index-10; ICIQ-SF, International Consultation on Incontinence Questionnaire – Short Form.

Supplementary Table S4. Characteristics of Studies Included in the Secondary Meta-Analysis on LLM and LUTS.

**Author (year)**	**Study Design**	**Gender**	**Mean Age (years) ± SD**	**Mean BMI (kg/m²) ± SD**	**Country**	**WHO Regions**	**LLM Sample Size (n)**	**Control Sample Size (n)**	**LUTS reported**	**LUTS Cases in LLM Group (n)**	**LUTS Cases in Control Group (n)**	**LLM Criteria**	**LUTS Assessment Tools**
C, P.-A., et al. (2017)	Prospective cohort	F	74.6 ± 2.9	28.3 ± 5.7	USA	Region of the Americas	101	561	UI	27	191	ASMI <5.5 kg/m²	Structured non-validated questionnaires or logs: *Self-reported questionnaire*
F, Z. and L. W (2025)	Cross-sectional	M&F	45.3 ± 13.3	34.9 ± 7.2	USA	Region of the Americas	376	4177	SUI<br>UUI<br>MUI	181<br>104<br>77	1537<br>882<br>530	ASM/BMI <0.789 for males and <0.512 for females.	Structured non-validated questionnaires or logs: *Kidney Conditions-Urology Questionnaire*
FX, S., et al. (2024)	Cross-sectional	F	46.0 ± 13.3	NA	USA	Region of the Americas	355	3202	SUI<br>UUI<br>MUI	158<br>92<br>73	1250<br>733<br>464	ASM/BMI <0.512	Structured non-validated questionnaires or logs: *Kidney Conditions-Urology Questionnaire*
G, P., et al. (2020)	Prospective cohort	M&F	57.0 ± 18.0	24.6 ± 4.8	Italy	European Region	12	22	OAB	10	5	ASMI <7.26 kg/m2 for males and <5.45 kg/m2 for females	Validated standardized questionnaire: *ICIQ-FLUTS, ICIQ-MLUTS, ICIQ-OAB*
HA, P., et al. (2024)	Cross-sectional	M&F	87.3 ± 5.2	25.7 ± 5.3	Brazil	Region of the Americas	126	160	UI	67	94	Calf circumference <34 cm for	Clinician-diagnosed or medical record-based classification

												males and <33 cm for females	
L, L., et al. (2023)	Prospective cohort	M&F	85.4 ± 11.7	NA	China	Western Pacific Region	10013	4976	UI	866	179	Calf circumference <34 cm for males and <33 cm for females	Structured non-validated questionnaires or logs: *Self-reported questionnaire*
M, H., et al. (2021)	Cross-sectional	F	74.0 ± 8.3	22.0 ± 6.3	Japan	Western Pacific Region	70	69	IPSS-defined LUTS	5	8	PMA (cut-off not specified)	Validated standardized questionnaire: *IPSS*
N, O., et al. (2023)	Retrospective cohort	M	68.0 ± 23.7	23.5 ± 11.6	Japan	Western Pacific Region	92	154	IPSS-defined LUTS	50	59	SMI <43 cm^2/m^2 (BMI <25) or <53 cm^2/m^2 (BMI ≥25)	Validated standardized questionnaire: *IPSS*
T, E., et al. (2019)	Cross-sectional	F	73.8 ± 7.1	30.6 ± 5.5	Turkey	European Region	595	207	UI	311	81	SMM/height^2 <7.4 kg/m^2, SMM/weight <33.6%, or SMM/BMI <0.823	Clinician-diagnosed or medical record-based classification
W, C., et al. (2025)	Cross-sectional	F	63.4 ± 13.1	24.4 ± 4.0	Taiwan	Western Pacific Region	100	156	SUI	64	99	ASMI < 5.7 kg/m2	Structured non-validated questionnaires or logs: *Activity-based SUI severity grading (Grade 0-IV)*
W, S., et al. (2023)	Cross-sectional	M&F	39.0 ± 14.8	NA	USA	Region of the Americas	2600	6146	OAB	592	621	ASM/BMI <0.793	Validated standardized questionnaire: *OABSS*

Y, T., et al. (2019)	Cross-sectional	F	67.4 ± 5.2	22.1 ± 3.1	Japan	Western Pacific Region	252	955	Nocturia	45	131	ASMI <5.7 kg/m²	Structured non-validated questionnaires or logs: *Sleep diary*
Z, Q., et al. (2021)	Cross-sectional	M	54.5 ± 8.9	30.1 ± 3.0	USA	Region of the Americas	143	816	UF<br>Nocturia<br>Hesitancy<br>Incomplete emptying<br>Custom symptom-defined LUTS	26<br>40<br>24<br>21<br>51	105<br>203<br>48<br>79<br>197	ASM/BMI <0.789	Structured non-validated questionnaires or logs: *Self-reported questionnaire*

Abbreviations: LLM, Low lean mass; LUTS, Lower urinary tract symptoms; SD, Standard deviation; WHO, World Health Organization; BMI, Body Mass Index; CI, Confidence interval; NA, Not applicable; USA, United State of America; M, Male; F, Female; M&F, Male and female; UI, Urinary incontinence; SUI, Stress urinary incontinence; UUI, Urgency urinary incontinence; MUI, Mixed urinary incontinence; OAB, Overactive bladder; UF, Urinary frequency; ASMI, Appendicular Skeletal Muscle Mass Index; ASM, Appendicular Skeletal Muscle mass; PMA, Psoas Muscle Area; SMM, Skeletal Muscle Mass; IPSS, International Prostate Symptom Score; ICIQ-FLUTS, International Consultation on Incontinence Questionnaire – Female Lower Urinary Tract Symptoms; ICIQ-MLUTS, International Consultation on Incontinence Questionnaire – Male Lower Urinary Tract Symptoms; ICIQ-OAB, International Consultation on Incontinence Questionnaire – Overactive Bladder; OABSS, Overactive Bladder Symptom Score.

Supplementary Table S5. Characteristics of Studies Included in the Secondary Meta-Analysis on LGS and LUTS.

Author (year)	Study Design	Gender	Mean Age (years) ± SD	Mean BMI (kg/m²) ± SD	Country	WHO Regions	LGS sample size (n)	Control Sample Size (n)	LUTS Reported	LUTS Cases in LGS Group (n)	LUTS Cases in Control Group (n)	LGS Criteria	LUTS Assessment Tools
Y, T., et al. (2019)	Cross-sectional	F	67.4 ± 5.2	22.1 ± 3.1	Japan	Western Pacific Region	19	1188	Nocturia	3	17	Gait speed <1.0 m/s	Structured non-validated questionnaires or logs: *Sleep diary*
C, P.-A., et al. (2017)	Prospective cohort	F	74.6 ± 2.9	28.3 ± 5.7	USA	Region of the Americas	169	492	UI	60	157	Gait speed <1.1 m/s	Structured non-validated questionnaires or logs: *Self-reported questionnaire*
SR, B., et al. (2024)	Prospective cohort	M&F	75.8 ± 4.5	27.6 ± 4.4	USA	Region of the Americas	173	468	LURN SI-10-defined LUTS	75	151	Gait speed <0.95 m/s	Validated standardized questionnaire: *LURN SI-10*

Abbreviations: LGS, Low gait speed; LUTS, Lower urinary tract symptoms; SD, Standard deviation; WHO, World Health Organization; BMI, Body Mass Index; CI, Confidence interval; USA, United State of America; F, Female; M&F, Male and female; UI, Urinary incontinence; LURN SI-10, Lower Urinary Tract Dysfunction Research Network Symptom Index-10.

Supplementary Table S6. Characteristics of Studies Included in the Secondary Meta-Analysis on sarcopenia risk and LUTS.

**Author (year)**	**Study Design**	**Gender**	**Mean Age (years) ± SD**	**Mean BMI (kg/m^2) ± SD**	**Country**	**WHO Regions**	**SARC-F ≥4 Sample Size (n)**	**Control Sample Size (n)**	**LUTS Reported**	**LUTS Cases in SARC-F ≥4 Group (n)**	**LUTS Cases in Control Group (n)**	**Sarcopenia Risk Screening Criteria**	**LUTS Assessment Tools**
S, I., et al. (2019)	Cross-sectional	M&F	73.4 ± 6.5	24.9 ± 4.2	Japan	Western Pacific Region	74	255	OAB	24	38	SARC-F ≥4	Validated standardized questionnaire: *OABSS*
N, W., et al. (2025)	Cross-sectional	M	80.3 ± 5.6	23.9 ± 2.9	Japan	Western Pacific Region	16	43	OAB<br>IPSS-defined LUTS	5<br>13	11<br>21	SARC-F ≥4	Validated standardized questionnaire: *OABSS and IPSS*
AMD, d. S. V., et al. (2024)	Cross-sectional	F	62.8 ± 19.6	25.5 ± 5.1	Portugal	European Region	14	51	UI	11	21	SARC-F ≥4	Clinician-diagnosed or medical record-based classification

Abbreviations: SARC-F, Strength, Assistance with walking, Rise from a chair, Climb stairs, and Falls; LUTS, Lower urinary tract symptoms; SD, Standard deviation; WHO, World Health Organization; BMI, Body Mass Index; CI, Confidence interval; M, Male; F, Female; M&F, Male and female; OAB, Overactive bladder; UI, Urinary incontinence; OAB, Overactive bladder; OABSS, Overactive Bladder Symptom Score; IPSS, International Prostate Symptom Score.

Supplementary Table S7. Univariate Random-Effects Meta-Regression of the Association Between Sarcopenia and LUTS.

Moderator Variable (Covariate)	Category/Value	Coefficient (β)	Standard Error (SE)	95% CI for β	p-value	R^2	Interpretation
**Mean age**	Continuous	0.01	0.02	-0.03 to 0.05	0.64	0.00	NSE
**Mean BMI**	Continuous	-0.08	0.06	-0.22 to 0.07	0.26	0.00	NSE
**Gender distribution**	Male-only	Ref.	-	-	-	0.00	-
	Female-only	-0.85	0.54	-2.07 to 0.37	0.15		NSE
	Both	-0.06	0.54	-1.27 to 1.15	0.91		NSE
**Diagnostic criteria for sarcopenia**	AWGS 2014	Ref.	-	-	-	0.00	-
	AWGS 2019	-0.33	0.56	-1.67 to 1.00	0.58		NSE
	EWGSOP1	-0.40	0.84	-2.38 to 1.58	0.64		NSE
	EWGSOP2	-0.85	0.63	-2.35 to 0.64	0.22		NSE
	3 standard cut-offs	-1.04	0.43	-0.02 to 2.02	0.05		NSE
**LUTS Assessment Tools**	Validated standardized questionnaire	Ref.	-	-	-	0.00	-
	Clinician-diagnosed or medical record-based classification	0.59	0.47	-0.48 to 1.66	0.24		NSE
	Structured non-validated questionnaires or logs	0.01	0.53	-1.18 to 1.21	0.98		NSE
**WHO regions**	European	Ref.	-	-	-	0.00	-
	Americas	-0.84	0.73	-2.5 to 0.85	0.28		NSE
	South-East Asia	-0.17	0.94	-2.3 to 2.00	0.86		NSE
	Western Pacific	0.16	0.65	-1.34 to 1.66	0.81		NSE
	Eastern Mediterranean	-	-	-	-		-
	African	-	-	-	-		-
**Risk of bias**	Low	Ref.	-	-	-	0.00	-
	Moderate	0.28	0.56	-0.98 to 1.53	0.64		NSE
	High	-	-	-	-		-

Abbreviations: LUTS, Lower urinary tract symptoms; BMI, Body Mass Index; WHO, World Health Organization; CI, Confidence interval; Ref., Reference; NSE, No significant effect.

Supplementary Table S8. Summary of Certainty of Evidence Using the GRADE Framework.

Outcome	Effect Estimate	No. of Studies (n)	Risk of Bias	Inconsistency	Indirectness	Imprecision	Publication Bias	Overall Certainty
**Sarcopenia and LUTS (primary)**	OR = 1.77 (95% CI: 1.29–2.45)	12	Not serious	Serious	Not serious	Not serious	Not detected	Moderate
**Low lean mass (LLM)**	OR = 1.52 (95% CI: 1.19–1.95)	13	Not serious	Serious	Not serious	Not serious	Not detected	Moderate
**Low muscle strength (LMS)**	OR = 0.94 (95% CI: 0.47–1.89)	5	Not serious	Serious	Not serious	Serious	Not detected	Low
**Low gait speed (LGS)**	OR = 1.37 (95% CI: 1.06–1.76)	3	Not serious	Serious	Not serious	Not serious	Not detected	Moderate
**SARC-F ≥4**	OR = 3.20 (95% CI: 1.92–5.33)	3	Not serious	Serious	Not serious	Not serious	Not detected	Moderate

Abbreviations: LUTS, Lower urinary tract symptoms; SARC-F, Strength, Assistance with walking, Rise from a chair, Climb stairs, and Falls.

Supplementary Table S9. Summary of Diagnostic Criteria for Sarcopenia According to AWGS and EWGSOP Guidelines

Consensus Group	Low Muscle Mass (ASMI, kg/m^2)	Low Muscle Strength (Grip Strength, kg)	Low Physical Performance	Diagnostic Notes
AWGS 2014	Men <7.0; Women <5.4	Men <26; Women <18	Gait speed <0.8 m/s	Low muscle mass + (low strength or low performance)
AWGS 2019	Men <7.0; Women <5.7	Men <28; Women <18	Gait speed <1.0 m/s	Possible sarcopenia: low strength ± performance; Confirmed: includes low mass
EWGSOP1 (2010)	Men <7.26; Women <5.45	Men <30; Women <20	Gait speed <0.8 m/s	Low muscle mass + (low strength or low performance)
EWGSOP2 (2019)	Men <7.0; Women <5.5	Men <27; Women <16	Gait speed <0.8 m/s, SPPB ?8, TUG ?20 seconds, or 400-meter walk time >5 minutes.	Stepwise: probable (strength), confirmed (strength + mass), severe (+performance)

Abbreviations: LUTS, Lower urinary tract symptoms; AWGS, Asian Working Group for Sarcopenia; EWGSOP, European Working Group on Sarcopenia in Older People; SPPB, Short Physical Performance Battery; TUG, Timed Up and Go.

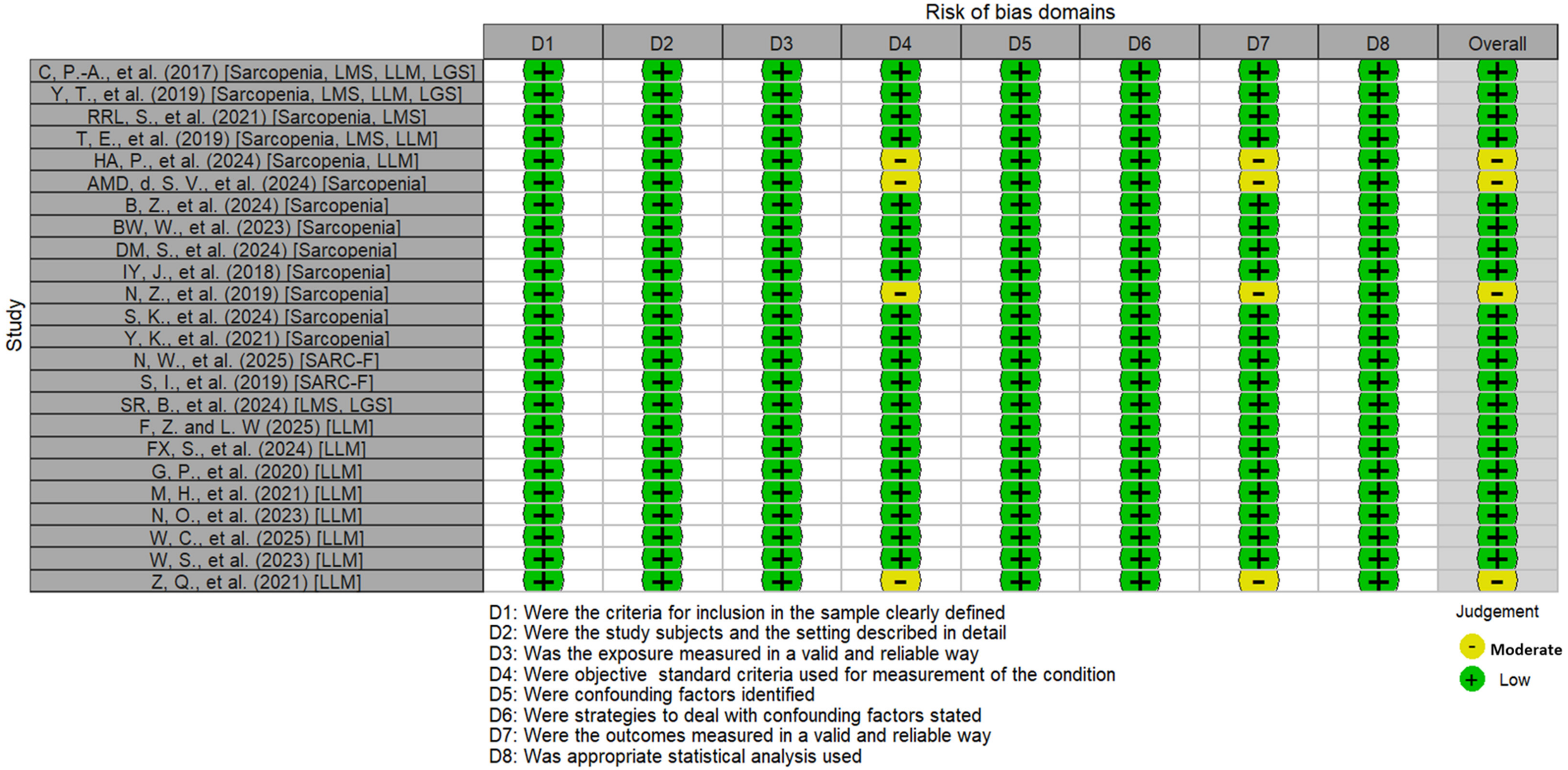

Supplementary Figure S1. Traffic light plot showing the risk of bias assessment across eight domains based on the JBI critical appraisal checklist for analytical cross-sectional studies.

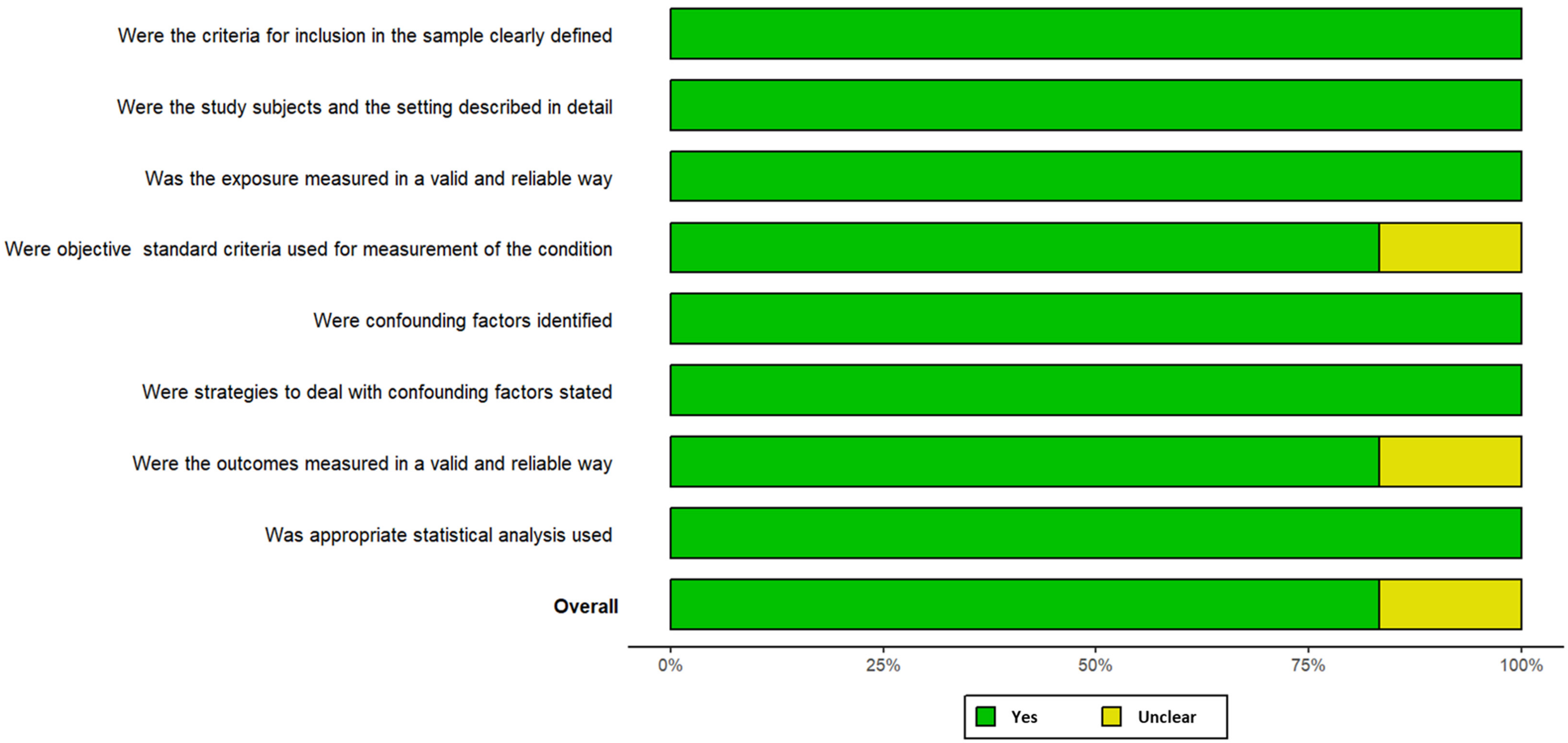

Supplementary Figure S2. Summary plot of the risk of bias assessment based on the JBI critical appraisal checklist for analytical cross-sectional studies. The plot displays the proportion of studies rated as “Yes” (low risk of bias) or “Unclear” (moderate risk of bias) across each of the eight domains.

Group by Diagnostic criteria	Study name	Statistics for each study			
		Odds ratio	Lower limit	Upper limit	p-Value
AWGS 2014	Y, T., et al. (2019)	1.678	0.941	2.991	0.079
AWGS 2014	IY, J., et al. (2018)	2.864	1.920	4.272	0.000
AWGS 2014	N, Z., et al. (2019)	4.122	2.402	7.074	0.000
AWGS 2014	Pooled	2.718	1.222	6.046	0.014
AWGS 2019	BW, W., et al. (2023)	0.909	0.677	1.221	0.527
AWGS 2019	S, K., et al. (2024)	1.313	0.692	2.493	0.405
AWGS 2019	B, Z., et al. (2024)	1.691	1.576	1.813	0.000
AWGS 2019	Y, K., et al. (2021)	6.965	4.934	9.832	0.000
AWGS 2019	Pooled	1.952	0.998	3.819	0.051

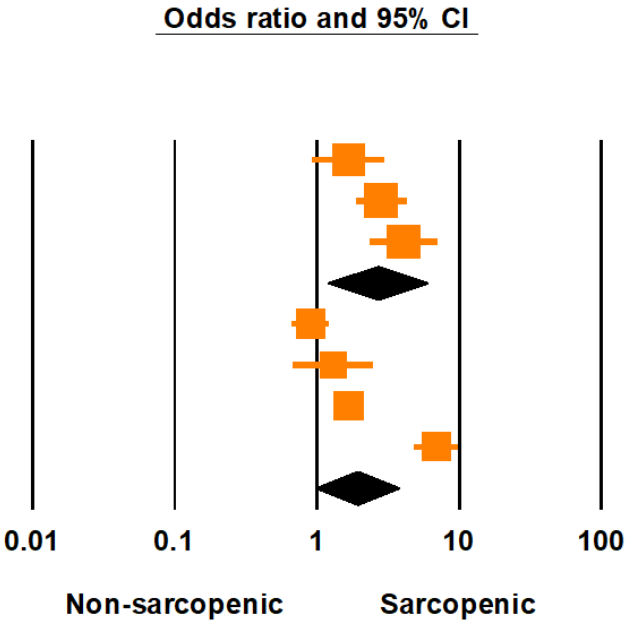

Supplementary Figure S3. Forest plot showing the subgroup analysis comparing individuals with sarcopenia diagnosed using AWGS 2014 versus AWGS 2019 criteria in relation to LUTS. High heterogeneity was observed ($I^2 = 93.6\%$, df = 6, $p < 0.001$), and the between-group difference was not statistically significant ($Q = 0.387$, df = 1, $p = 0.534$).

Group by LUTS types	Study name	Statistics for each study				Odds ratio and 95% CI
		Odds ratio	Lower limit	Upper limit	p-Value	
Other LUTS	S, K., et al. (2024)	1.313	0.692	2.493	0.405	
Other LUTS	Y, T., et al. (2019)	1.678	0.941	2.991	0.079	
Other LUTS	DM, S., et al. (2024)	2.267	1.123	4.576	0.022	
Other LUTS	IY, J., et al. (2018)	2.864	1.920	4.272	0.000	
Other LUTS	Pooled	1.972	1.076	3.617	0.028	
UI	RRL, S., et al. (2021)	0.487	0.193	1.227	0.127	
UI	BW, W., et al. (2023)	0.909	0.677	1.221	0.527	
UI	C, P.-A., et al. (2017)	0.956	0.694	1.318	0.784	
UI	HA, P., et al. (2024)	1.233	0.751	2.025	0.408	
UI	B, Z., et al. (2024)	1.691	1.576	1.813	0.000	
UI	T, E., et al. (2019)	1.816	1.329	2.480	0.000	
UI	N, Z., et al. (2019)	4.122	2.402	7.074	0.000	
UI	Y, K., et al. (2021)	6.965	4.934	9.832	0.000	
UI	Pooled	1.688	1.124	2.534	0.012	

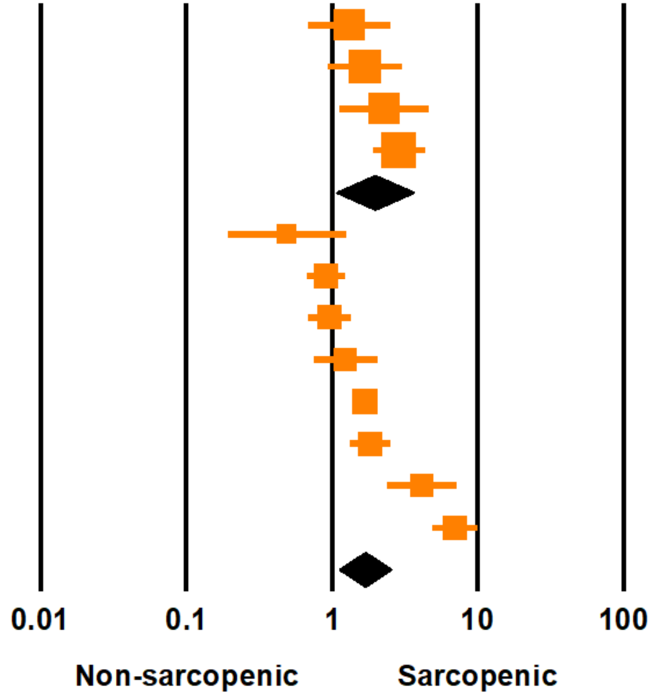

Supplementary Figure S4. Forest plot showing the subgroup analysis comparing individuals with sarcopenia who had urinary incontinence (UI) versus those without UI. High heterogeneity was observed (I^2 = 90.9%, df = 11, p < 0.001), and the between-group difference was not statistically significant (Q = 0.175, df = 1, p = 0.676).

Group by LUTS Assessment Tools	Study name	Odds ratio	Lower limit	Upper limit	p-Value
Clinician-diagnosed or medical record-based classification	HA, P., et al. (2024)	1.233	0.751	2.025	0.408
Clinician-diagnosed or medical record-based classification	S, K., et al. (2024)	1.313	0.692	2.493	0.405
Clinician-diagnosed or medical record-based classification	T, E., et al. (2019)	1.816	1.329	2.480	0.000
Clinician-diagnosed or medical record-based classification	N, Z., et al. (2019)	4.122	2.402	7.074	0.000
Clinician-diagnosed or medical record-based classification	Y, K., et al. (2021)	6.965	4.934	9.832	0.000
Clinician-diagnosed or medical record-based classification	Pooled	2.477	1.397	4.389	0.002
Structured non-validated questionnaires or logs	C, P.-A., et al. (2017)	0.956	0.694	1.318	0.784
Structured non-validated questionnaires or logs	Y, T., et al. (2019)	1.678	0.941	2.991	0.079
Structured non-validated questionnaires or logs	B, Z., et al. (2024)	1.691	1.576	1.813	0.000
Structured non-validated questionnaires or logs	Pooled	1.389	0.677	2.850	0.370
Validated standardized questionnaire	RRL, S., et al. (2021)	0.487	0.193	1.227	0.127
Validated standardized questionnaire	BW, W., et al. (2023)	0.909	0.677	1.221	0.527
Validated standardized questionnaire	DM, S., et al. (2024)	2.267	1.123	4.576	0.022
Validated standardized questionnaire	IY, J., et al. (2018)	2.864	1.920	4.272	0.000
Validated standardized questionnaire	Pooled	1.372	0.706	2.665	0.351

Statistics for each study

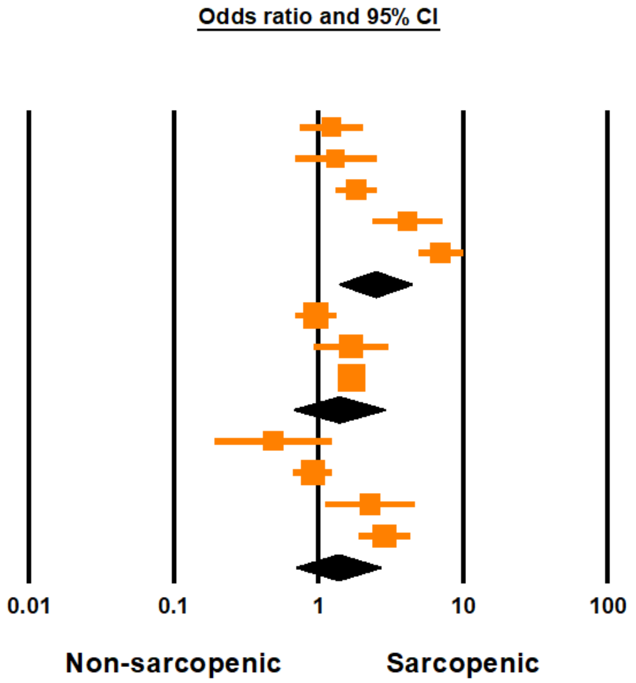

Supplementary Figure S5. Forest plot showing the subgroup analysis comparing individuals with sarcopenia based on the method used to diagnose LUTS: clinician-diagnosed or medical record–based classification, structured non-validated questionnaires or logs, and validated standardized questionnaires. High heterogeneity was observed ($I^2 = 90.9\%$, df = 11, $p < 0.001$), and the between-group difference was not statistically significant ($Q = 2.326$, df = 2, $p = 0.313$).

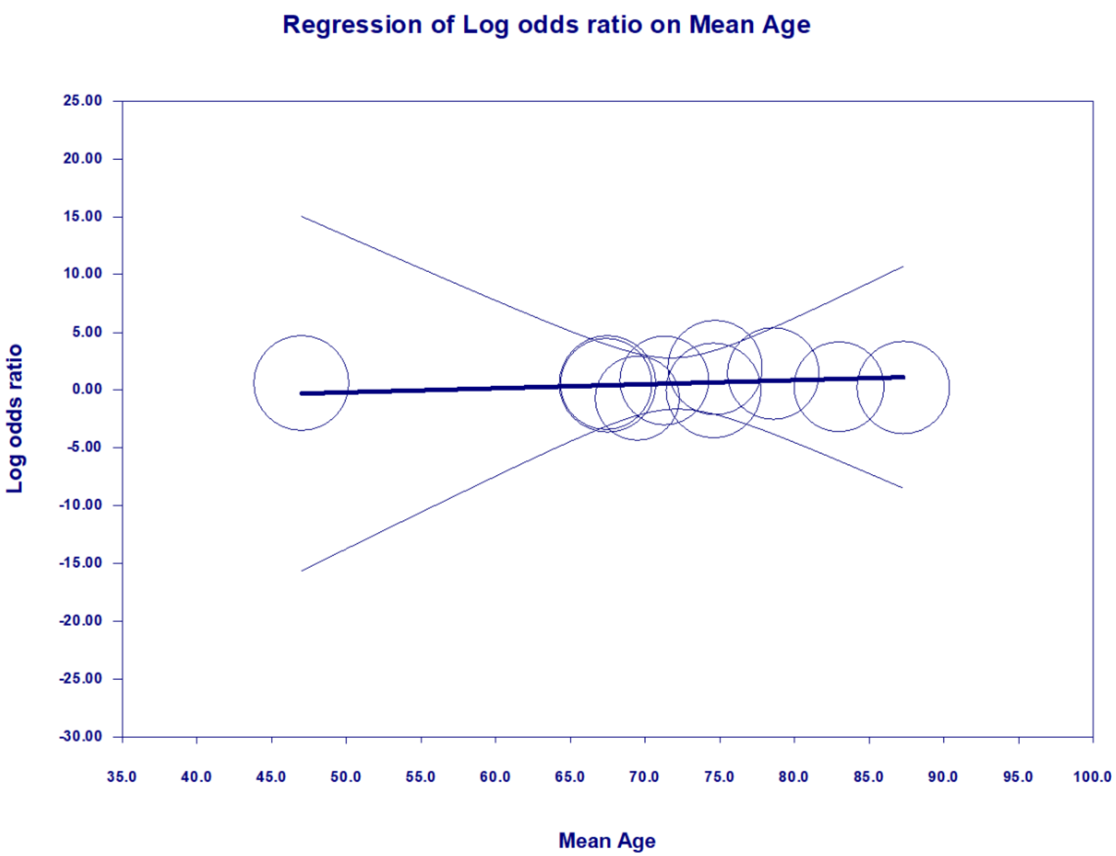
Regression of Log odds ratio on Mean Age
25.00
20.00
15.00
10.00
5.00
0.00
-5.00
-10.00
-15.00
-20.00
-25.00
-30.00
Log odds ratio
35.0
40.0
45.0
50.0
55.0
60.0
65.0
70.0
75.0
80.0
85.0
90.0
95.0
100.0
Mean Age

(a) Mean age

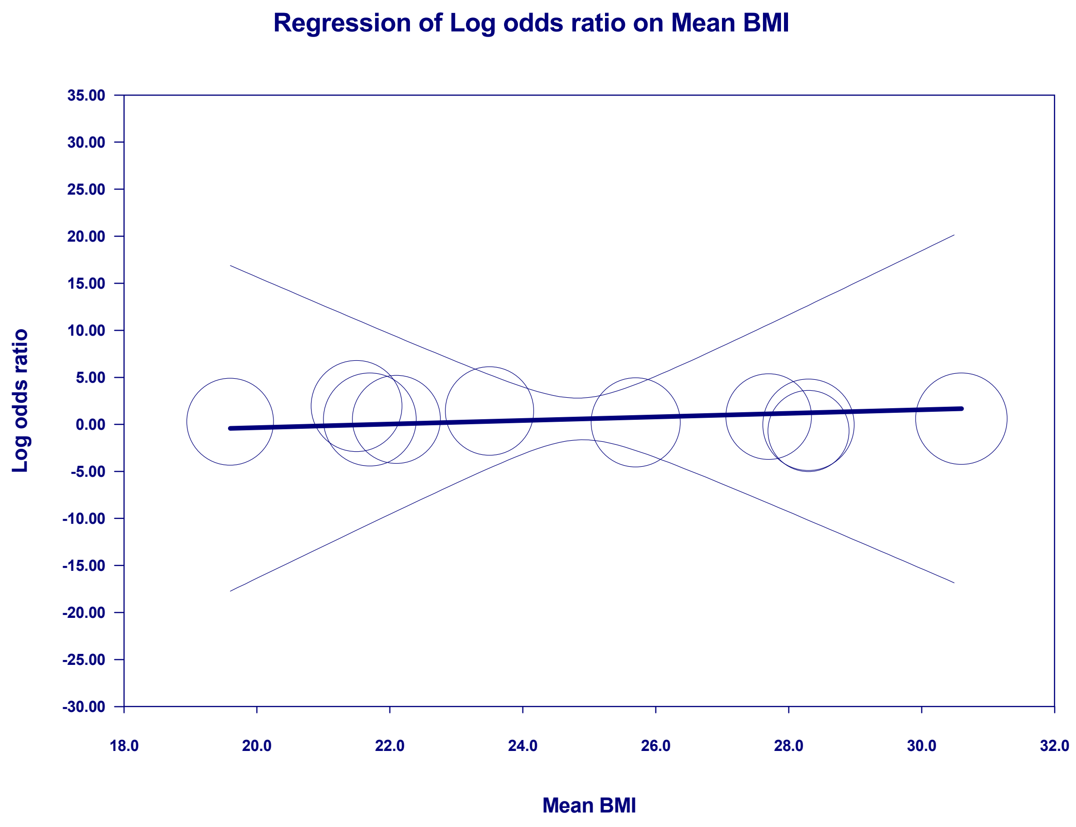
Regression of Log odds ratio on Mean BMI
35.00
30.00
25.00
20.00
15.00
10.00
5.00
0.00
-5.00
-10.00
-15.00
-20.00
-25.00
-30.00
Log odds ratio
18.0
20.0
22.0
24.0
26.0
28.0
30.0
32.0
Mean BMI

(b) Mean BMI

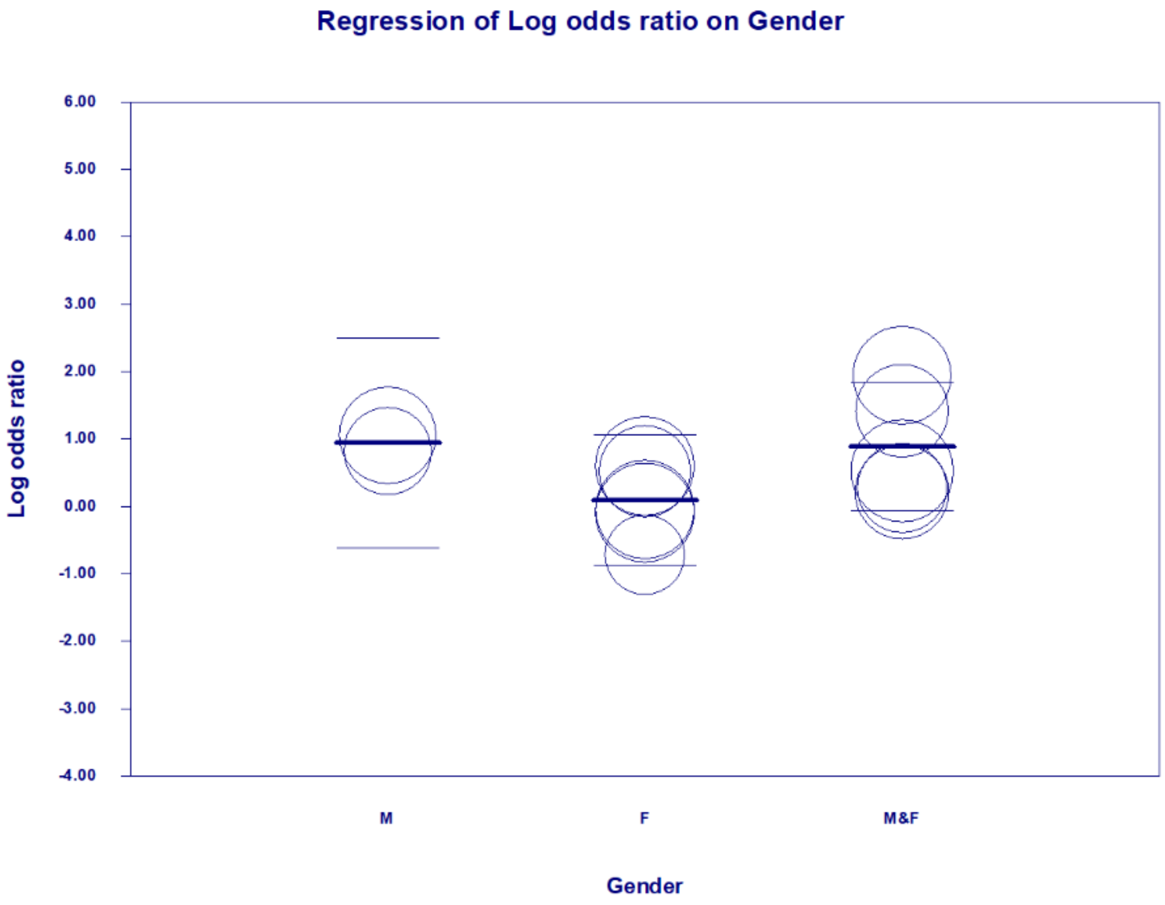

(c) Gender Distribution

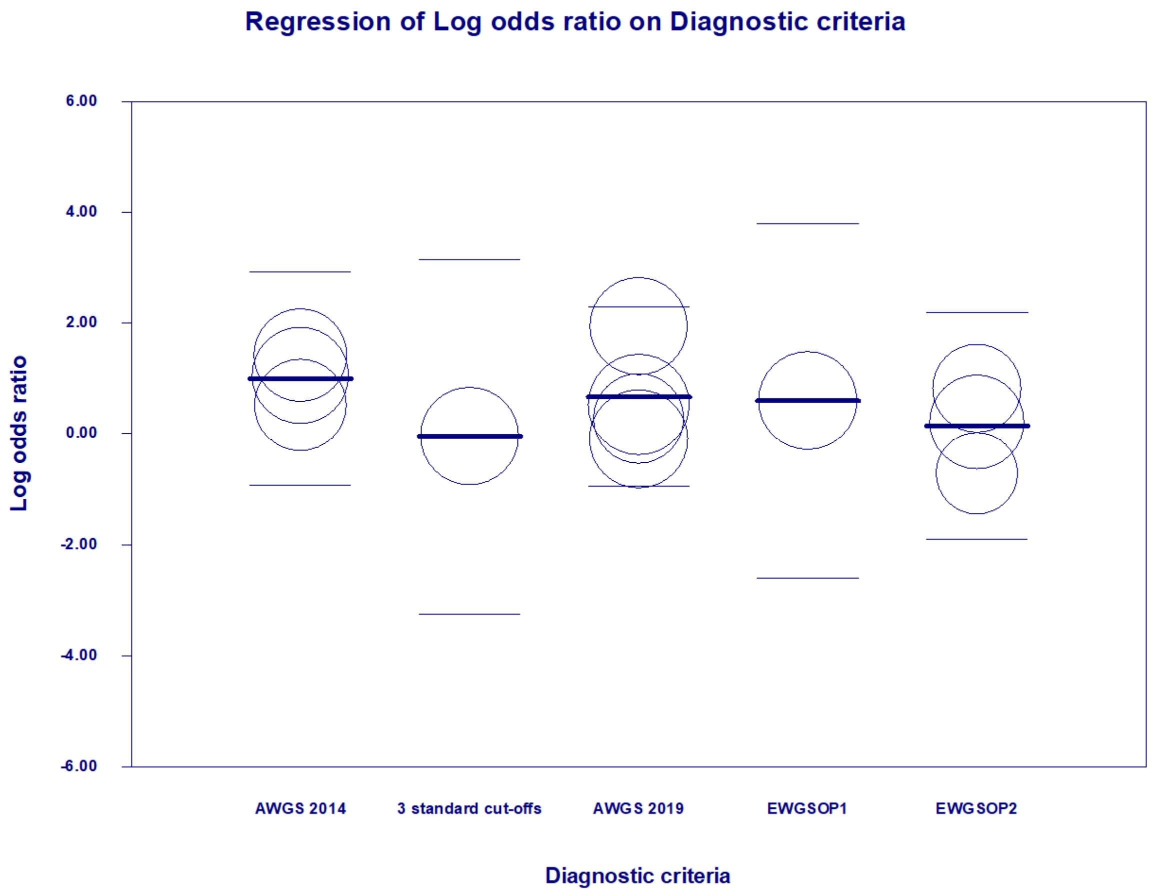

(d) Diagnostic Criteria for Sarcopenia

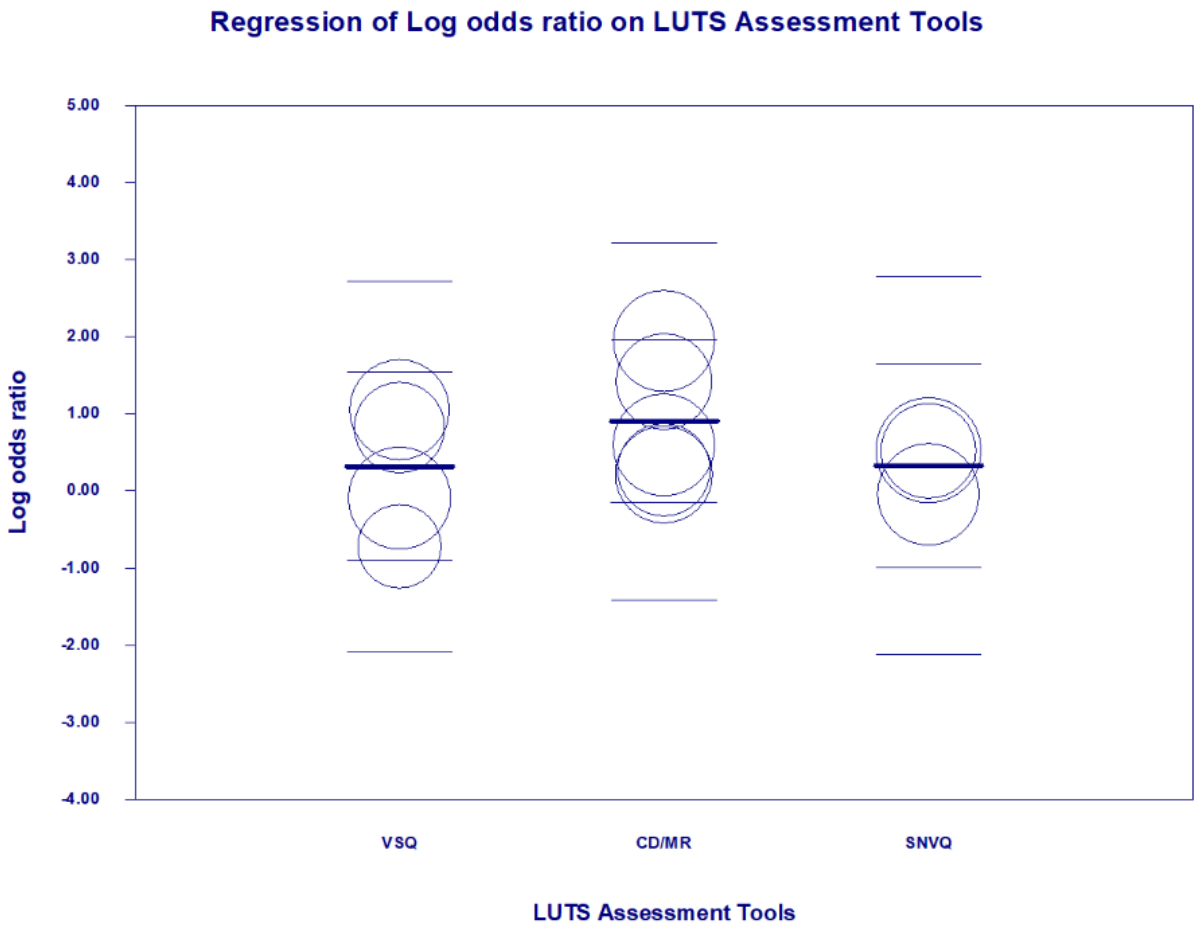

(e) LUTS Assessment Tools

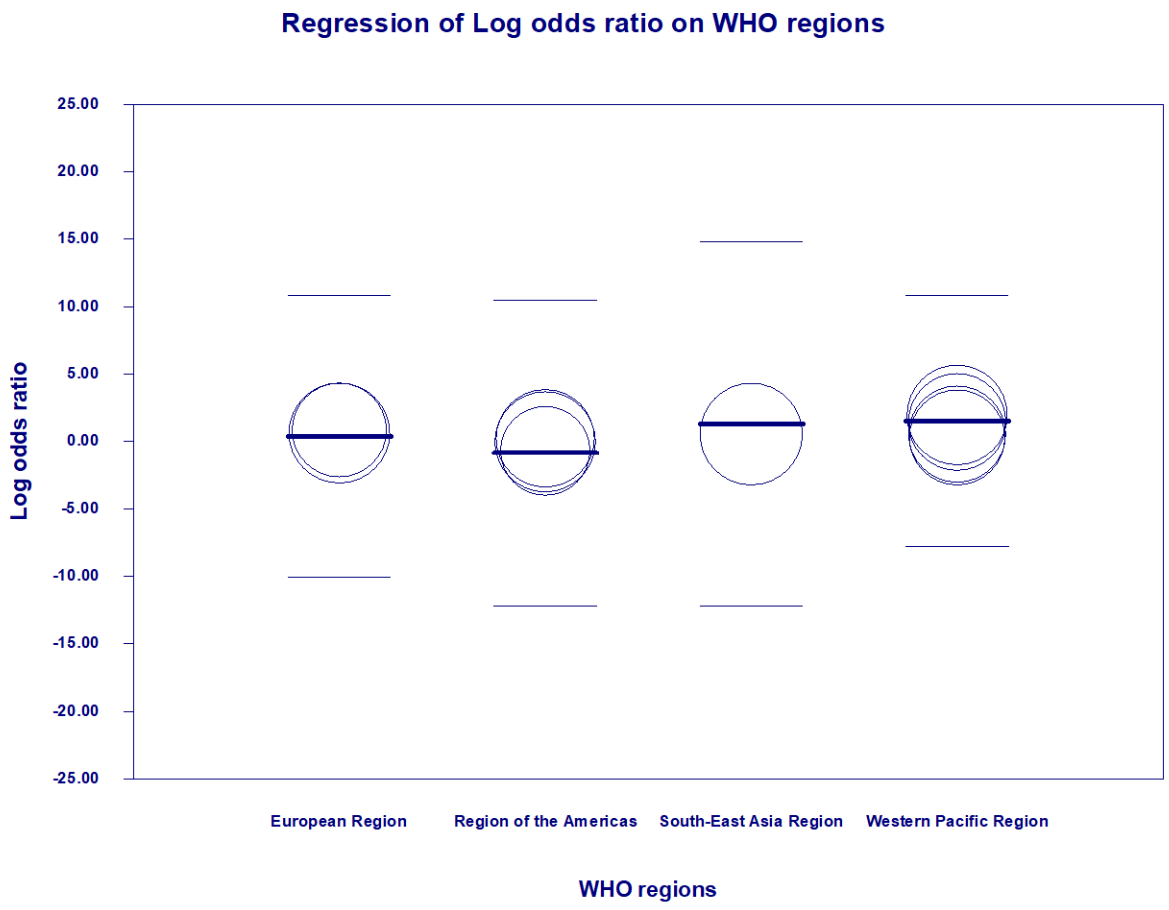

(f) WHO Regions

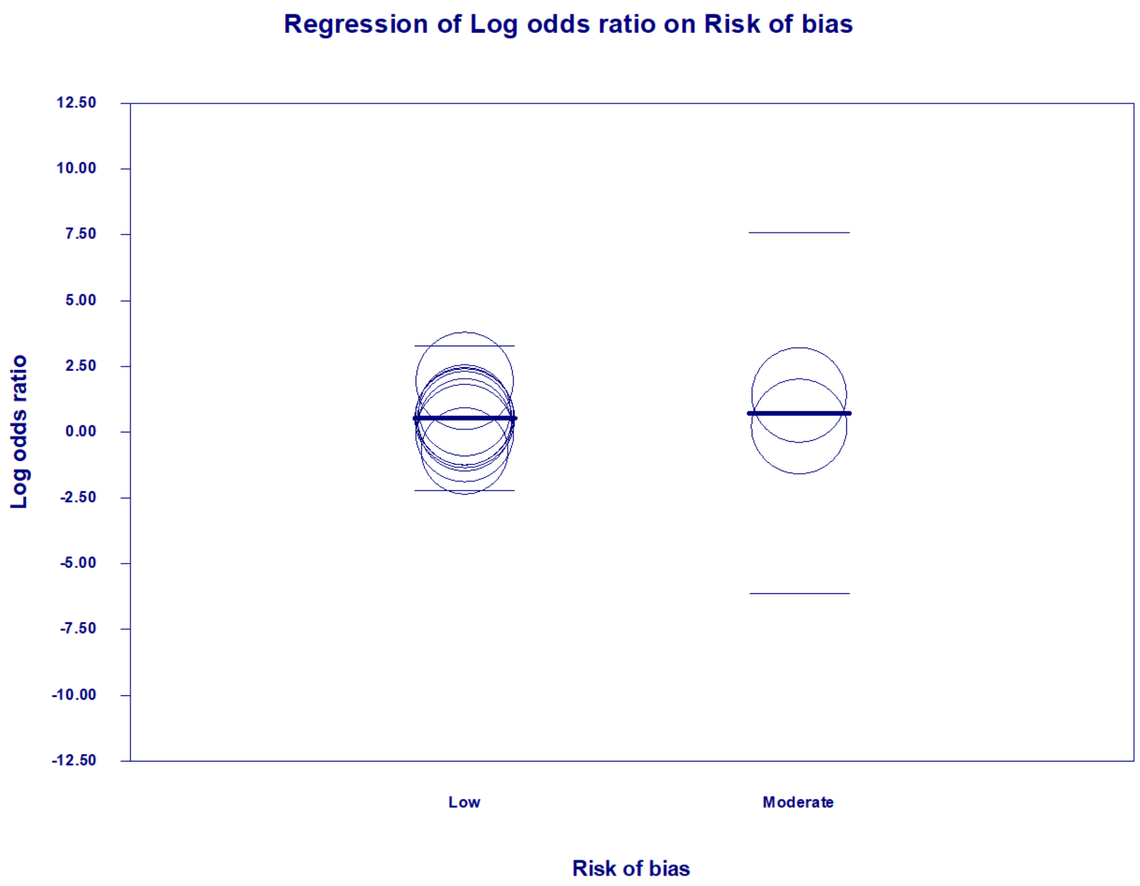

(g) Risk of Bias

Supplementary Figure S6. Univariate random-effects meta-regression analyses of the odds ratio (OR) for the association between sarcopenia and lower urinary tract symptoms (LUTS) according to: (a) mean age, (b) mean BMI, (c) gender distribution, (d) diagnostic criteria for sarcopenia, (e) LUTS assessment tools, (f) WHO region, and (g) risk of bias. Note: Circles represent individual studies, with size proportional to study weight. The central line indicates the fitted meta-regression line, and the outer lines represent the 95% confidence interval.

Abbreviation: VSQ, Validated standardized questionnaire; CD/MR, Clinician-diagnosed or medical record-based classification; SNVQ, Structured non-validated questionnaires or logs.

Study name	Point	Lower limit	Upper limit	p-Value
B, Z., et al. (2024)	1.761	1.115	2.782	0.015
BW, W., et al. (2023)	1.902	1.353	2.674	0.000
C, P.-A., et al. (2017)	1.889	1.340	2.664	0.000
DM, S., et al. (2024)	1.741	1.240	2.445	0.001
HA, P., et al. (2024)	1.832	1.298	2.586	0.001
IY, J., et al. (2018)	1.693	1.200	2.388	0.003
N, Z., et al. (2019)	1.651	1.186	2.300	0.003
RRL, S., et al. (2021)	1.915	1.381	2.654	0.000
S, K., et al. (2024)	1.816	1.292	2.553	0.001
T, E., et al. (2019)	1.765	1.227	2.539	0.002
Y, K., et al. (2021)	1.553	1.207	1.998	0.001
Y, T., et al. (2019)	1.781	1.263	2.511	0.001
Pooled	1.775	1.285	2.450	0.000

Statistics with study removed

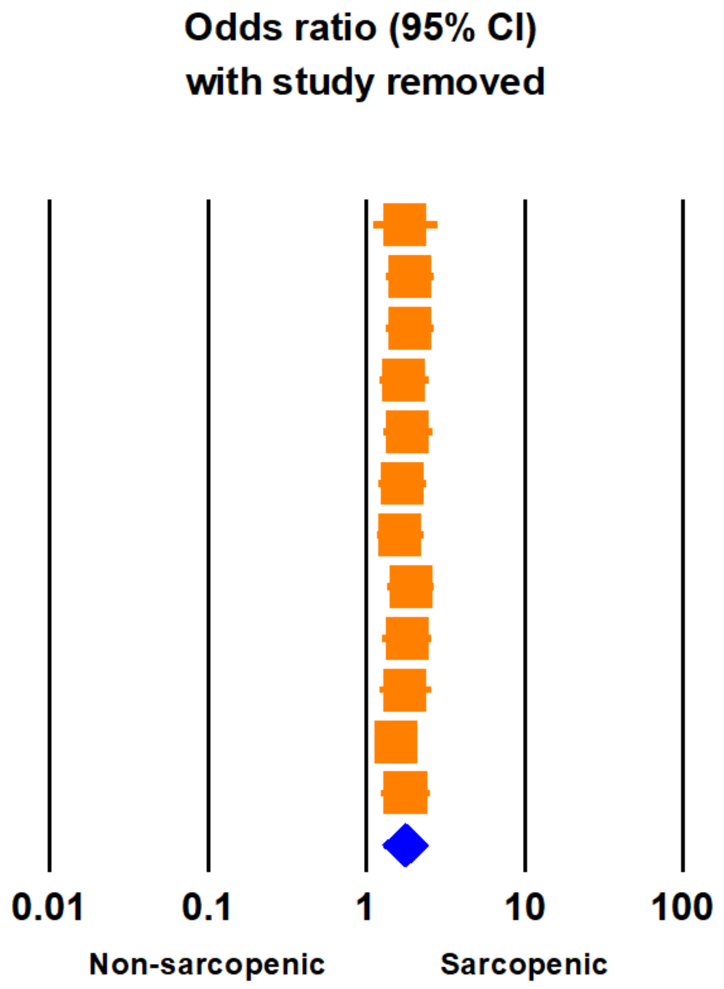

Supplementary Figure S7. Forest plot showing the results of the leave-one-out sensitivity analysis assessing the robustness of the pooled odds ratio for the association between sarcopenia and LUTS.

Group by Study Design	Study name	Odds ratio	Lower limit	Upper limit	p-Value
Cohort	C, P.-A., et al. (2017)	0.956	0.694	1.318	0.784
Cohort	Y, K., et al. (2021)	6.965	4.934	9.832	0.000
Cohort	Pooled	2.565	1.156	5.693	0.021
Cross-sectional	B, Z., et al. (2024)	1.691	1.576	1.813	0.000
Cross-sectional	BW, W., et al. (2023)	0.909	0.677	1.221	0.527
Cross-sectional	DM, S., et al. (2024)	2.267	1.123	4.576	0.022
Cross-sectional	HA, P., et al. (2024)	1.233	0.751	2.025	0.408
Cross-sectional	IY, J., et al. (2018)	2.864	1.920	4.272	0.000
Cross-sectional	N, Z., et al. (2019)	4.122	2.402	7.074	0.000
Cross-sectional	RRL, S., et al. (2021)	0.487	0.193	1.227	0.127
Cross-sectional	S, K., et al. (2024)	1.313	0.692	2.493	0.405
Cross-sectional	T, E., et al. (2019)	1.816	1.329	2.480	0.000
Cross-sectional	Y, T., et al. (2019)	1.678	0.941	2.991	0.079
Cross-sectional	Pooled	1.629	1.117	2.377	0.011

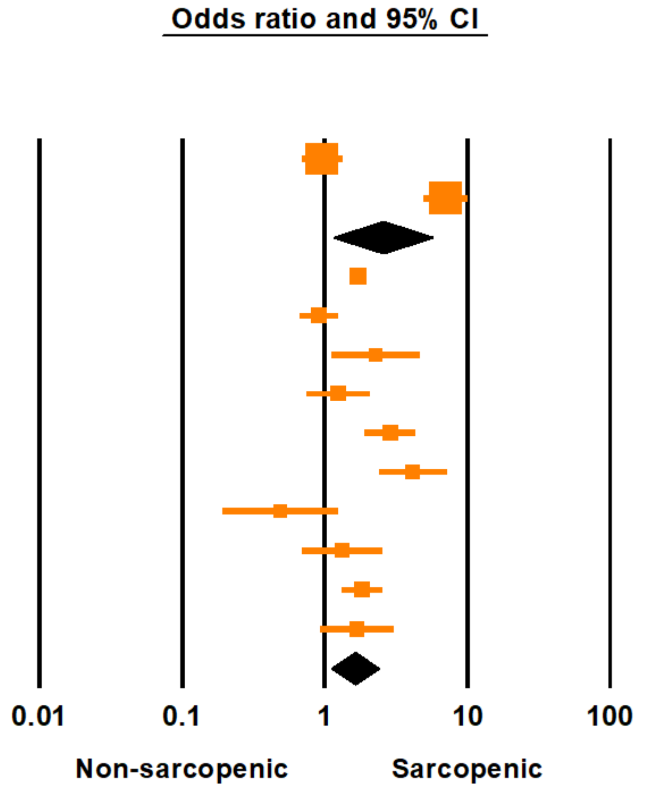

Supplementary Figure S8. Forest plot showing the subgroup analysis comparing study design (cohort vs. cross-sectional) in studies evaluating the association between sarcopenia and LUTS. High heterogeneity was observed ($I^2 = 90.9\%$, df = 11, $p < 0.001$), and the between-group difference was not statistically significant ($Q = 1.016$, df = 1, $p = 0.313$).

Study name	Statistics for each study				
	Odds ratio	Lower limit	Upper limit	Z-Value	p-Value
C, P.-A., et al. (2017)	0.707	0.440	1.135	-1.435	0.151
HA, P., et al. (2024)	0.797	0.498	1.277	-0.943	0.346
M, H., et al. (2021)	0.847	0.290	2.479	-0.303	0.762
W, C., et al. (2025)	1.024	0.607	1.726	0.087	0.930
FX, S., et al. (2024)	1.252	1.004	1.562	1.996	0.046
Y, T., et al. (2019)	1.367	0.943	1.982	1.652	0.099
F, Z. and L. W (2025)	1.594	1.290	1.971	4.316	0.000
Z, Q., et al. (2021)	1.674	1.148	2.441	2.677	0.007
T, E., et al. (2019)	1.703	1.234	2.351	3.240	0.001
N, O., et al. (2023)	1.917	1.136	3.235	2.437	0.015
L, L., et al. (2023)	2.537	2.152	2.991	11.082	0.000
W, S., et al. (2023)	2.623	2.318	2.968	15.288	0.000
G, P., et al. (2020)	17.000	2.765	104.540	3.057	0.002
Pooled	1.521	1.186	1.951	3.306	0.001
Prediction Interval	1.521	0.614	3.772		

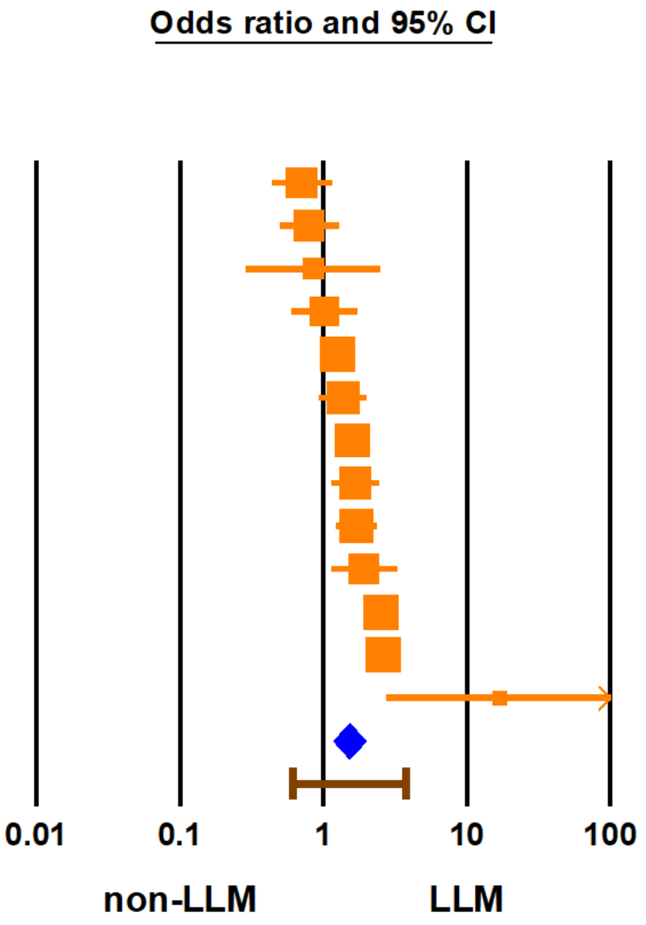

Supplementary Figure S9. Forest plot showing the pooled odds ratio (OR) for the association between low lean mass (LLM) and lower urinary tract symptoms (LUTS) across all included studies. Significant heterogeneity was observed ($I^2 = 88.1\%$, $p < 0.001$; Cochran's Q test).

Study name	Event rate	Lower limit	Upper limit
L, L., et al. (2023)	0.086	0.081	0.092
M, H., et al. (2021)	0.100	0.048	0.195
Y, T., et al. (2019)	0.179	0.136	0.231
W, S., et al. (2023)	0.228	0.212	0.244
C, P.-A., et al. (2017)	0.267	0.190	0.362
Z, Q., et al. (2021)	0.357	0.282	0.438
FX, S., et al. (2024)	0.445	0.394	0.497
F, Z. and L. W (2025)	0.481	0.431	0.532
T, E., et al. (2019)	0.523	0.482	0.563
HA, P., et al. (2024)	0.532	0.445	0.617
N, O., et al. (2023)	0.543	0.441	0.642
W, C., et al. (2025)	0.640	0.542	0.728
G, P., et al. (2020)	0.833	0.523	0.958
Pooled	0.365	0.235	0.520
Prediction Interval	0.365	0.041	0.885

(Column group heading: Statistics for each study)

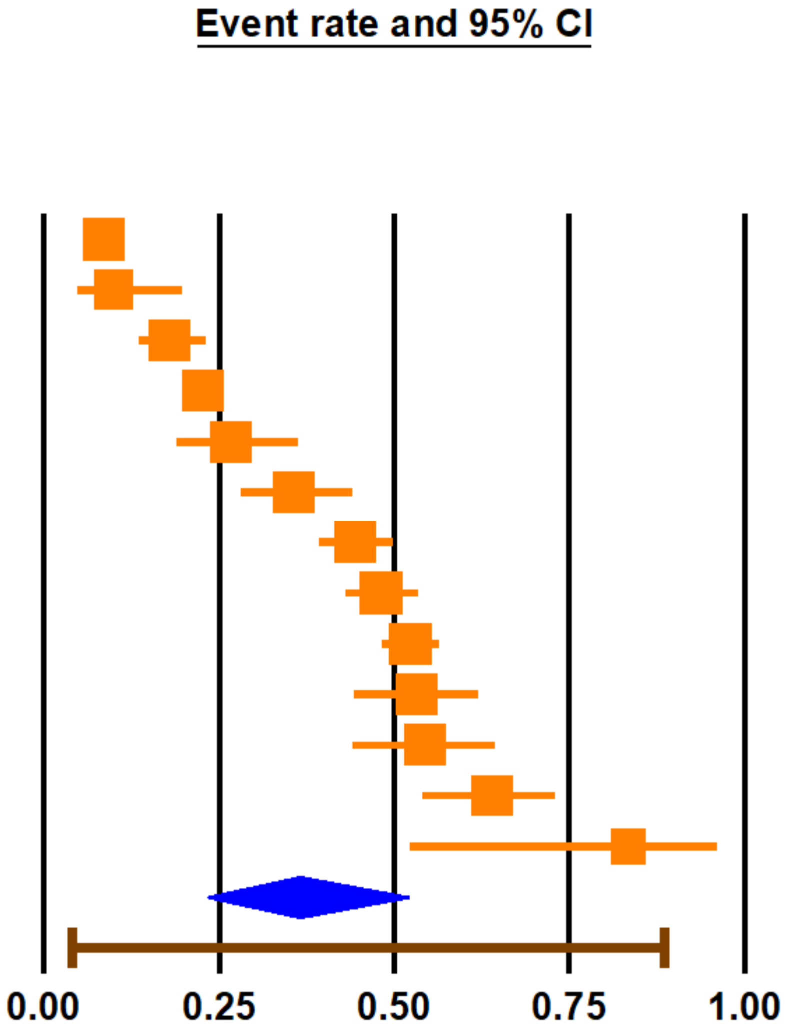

Supplementary Figure S10. Forest plot showing the pooled prevalence of lower urinary tract symptoms (LUTS) among individuals with LLM across all included studies. Significant heterogeneity was observed ($I^2 = 99.3\%$, $p < 0.001$, Cochran's Q test).

Study name	Statistics for each study				
	Odds ratio	Lower limit	Upper limit	Z-Value	p-Value
C, P.-A., et al. (2017)	0.247	0.163	0.374	-6.600	0.000
RRL, S., et al. (2021)	0.941	0.464	1.905	-0.169	0.865
SR, B., et al. (2024)	1.134	0.807	1.593	0.726	0.468
T, E., et al. (2019)	1.548	1.166	2.056	3.018	0.003
Y, T., et al. (2019)	1.805	1.174	2.775	2.693	0.007
Pooled	0.944	0.473	1.887	-0.162	0.871

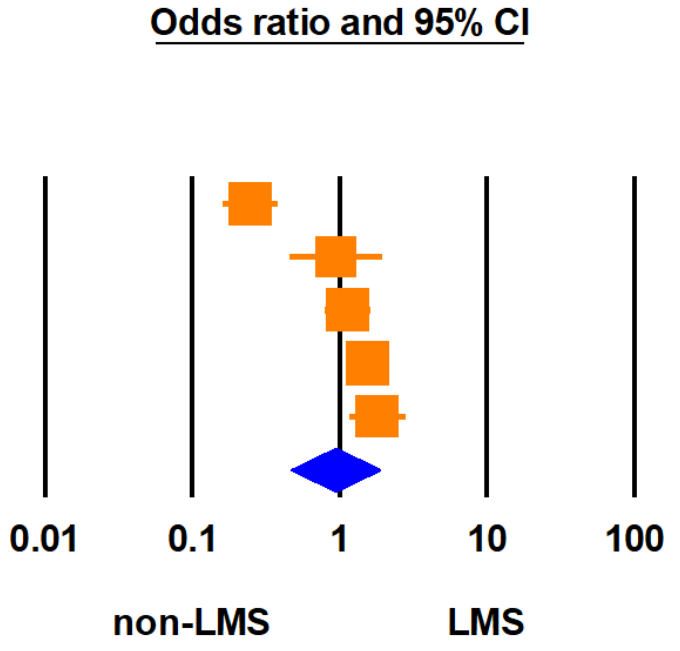

Supplementary Figure S11. Forest plot showing the pooled odds ratio (OR) for the association between low muscle strength (LMS) and lower urinary tract symptoms (LUTS) across all included studies. Significant heterogeneity was observed ($I^2 = 93.4\%$, $p < 0.001$; Cochran's Q test).

Study name	Event rate	Lower limit	Upper limit
Y, T., et al. (2019)	0.2207	0.1606	0.2954
C, P.-A., et al. (2017)	0.3333	0.2558	0.4211
SR, B., et al. (2024)	0.3710	0.3099	0.4366
T, E., et al. (2019)	0.5528	0.4981	0.6063
RRL, S., et al. (2021)	0.7746	0.6633	0.8571
Pooled	0.4440	0.2923	0.6068
Prediction Interval	0.4440	0.0587	0.9110

(The "Event rate", "Lower limit" and "Upper limit" columns are printed under the heading "Statistics for each study".)

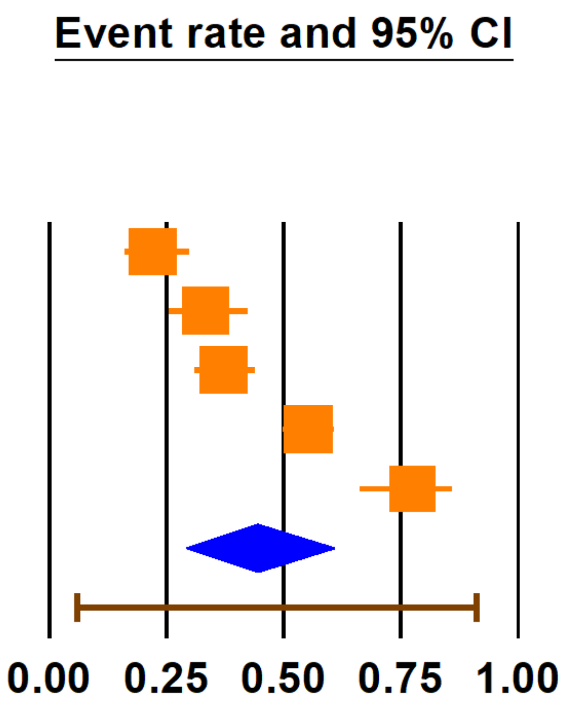

Supplementary Figure S12. Forest plot showing the pooled prevalence of lower urinary tract symptoms (LUTS) among individuals with low muscle strength (LMS) across all included studies. Significant heterogeneity was observed ($I^2 = 94.9\%$, $p < 0.001$; Cochran's Q test).

Study name	Statistics for each study				
	Odds ratio	Lower limit	Upper limit	Z-Value	p-Value
Y, T., et al. (2019)	1.100	0.317	3.815	0.150	0.881
C, P.-A., et al. (2017)	1.175	0.813	1.697	0.858	0.391
SR, B., et al. (2024)	1.607	1.123	2.298	2.598	0.009
Pooled	1.367	1.063	1.757	2.440	0.015

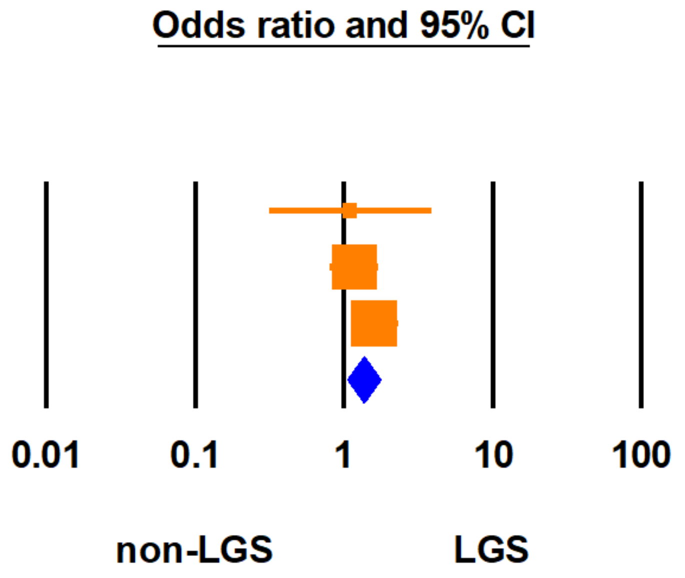

Supplementary Figure S13. Forest plot showing the pooled odds ratio (OR) for the association between low gait speed (LGS) and lower urinary tract symptoms (LUTS) across all included studies. No significant heterogeneity was observed ($I^2 = 0.0\%$, $p = 0.46$; Cochran's Q test).

Study name	Statistics for each study		
	Event rate	Lower limit	Upper limit
Y, T., et al. (2019)	0.1579	0.0518	0.3915
C, P.-A., et al. (2017)	0.3550	0.2866	0.4300
SR, B., et al. (2024)	0.4335	0.3617	0.5083
Pooled	0.3629	0.2673	0.4708
Prediction Interval	0.3629	0.0047	0.9860

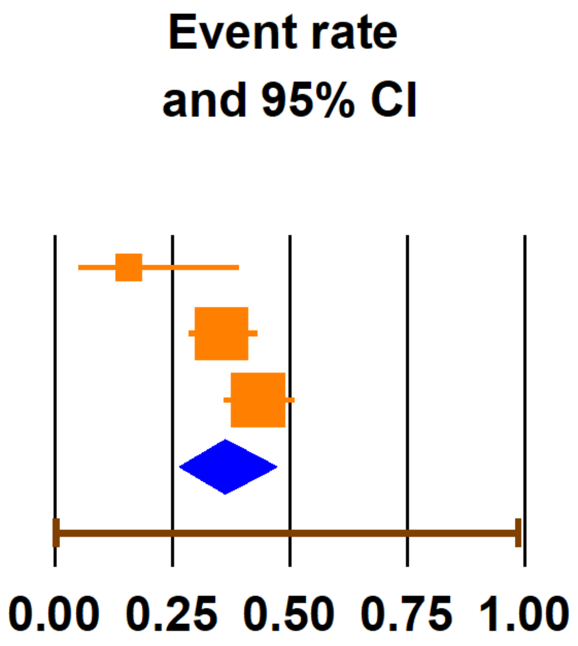

Supplementary Figure S14. Forest plot showing the pooled prevalence of lower urinary tract symptoms (LUTS) among individuals with low gait speed (LGS) across all included studies. Significant heterogeneity was observed ($I^2 = 66.8\%$, $p = 0.049$; Cochran's Q test).

Study name	Odds ratio	Lower limit	Upper limit	Z-Value	p-Value
S, I., et al. (2019)	2.741	1.510	4.977	3.314	0.001
N, W., et al. (2025)	4.540	1.130	18.235	2.132	0.033
AMD, d. S. V., et al. (2024)	5.238	1.301	21.095	2.330	0.020
Pooled	3.200	1.921	5.329	4.469	0.000

Statistics for each study

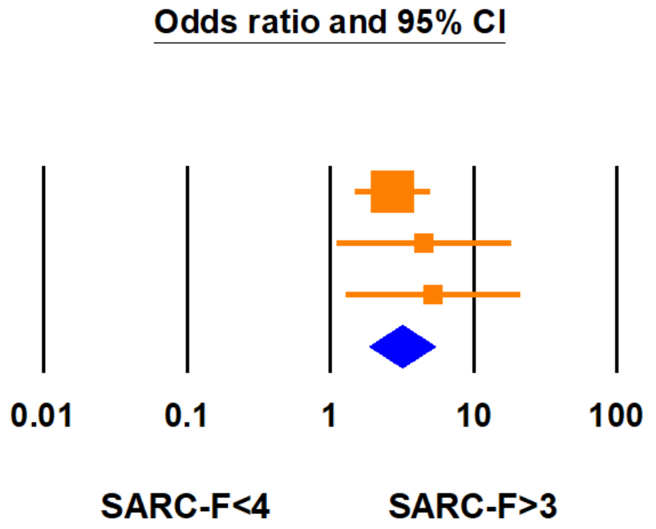

Supplementary Figure S15. Forest plot showing the pooled odds ratio (OR) for the association between risk of sarcopenia and lower urinary tract symptoms (LUTS) across all included studies. No significant heterogeneity was observed ($I^2 = 0.0\%$, $p = 0.612$; Cochran's Q test).

Study name	Statistics for each study		
	Event rate	Lower limit	Upper limit
S, I., et al. (2019)	0.324	0.228	0.438
AMD, d. S. V., et al. (2024)	0.786	0.506	0.929
N, W., et al. (2025)	0.813	0.553	0.938
Pooled	0.644	0.258	0.904

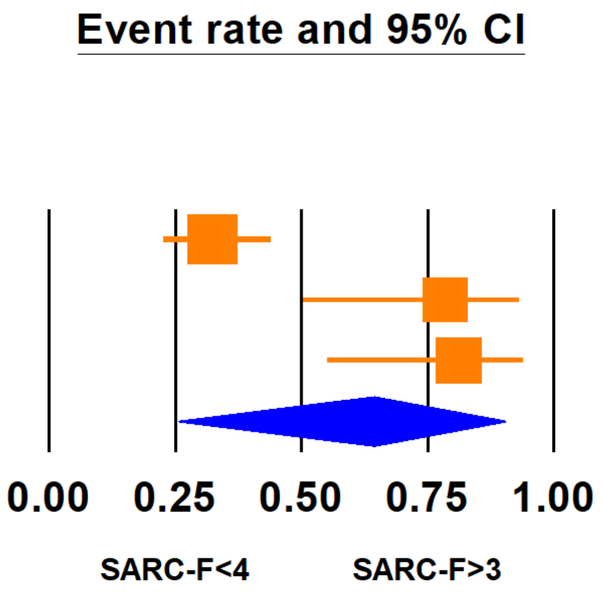

Supplementary Figure S16. Forest plot showing the pooled prevalence of lower urinary tract symptoms (LUTS) among individuals at risk of sarcopenia across all included studies. Significant heterogeneity was observed ($I^2 = 88.0\%$, $p < 0.001$; Cochran's Q test).

Group by Exposure	Study name	Statistics for each study				
		Odds ratio	Lower limit	Upper limit	Z-Value	p-Value
LLM	M, H., et al. (2021)	0.847	0.290	2.479	-0.303	0.762
LLM	W, C., et al. (2025)	1.024	0.607	1.726	0.087	0.930
LLM	FX, S., et al. (2024)	1.252	1.004	1.562	1.996	0.046
LLM	F, Z. and L. W (2025)	1.594	1.290	1.971	4.316	0.000
LLM	Z, Q., et al. (2021)	1.674	1.148	2.441	2.677	0.007
LLM	N, O., et al. (2023)	1.917	1.136	3.235	2.437	0.015
LLM	W, S., et al. (2023)	2.623	2.318	2.968	15.288	0.000
LLM	G, P., et al. (2020)	17.000	2.765	104.540	3.057	0.002
LLM	Pooled	1.684	1.154	2.456	2.705	0.007
Sarcopenia	RRL, S., et al. (2021)	0.487	0.193	1.227	-1.527	0.127
Sarcopenia	BW, W., et al. (2023)	0.909	0.677	1.221	-0.633	0.527
Sarcopenia	C, P.-A., et al. (2017)	0.956	0.694	1.318	-0.274	0.784
Sarcopenia	HA, P., et al. (2024)	1.233	0.751	2.025	0.827	0.408
Sarcopenia	S, K., et al. (2024)	1.313	0.692	2.493	0.833	0.405
Sarcopenia	Y, T., et al. (2019)	1.678	0.941	2.991	1.754	0.079
Sarcopenia	B, Z., et al. (2024)	1.691	1.576	1.813	14.681	0.000
Sarcopenia	T, E., et al. (2019)	1.816	1.329	2.480	3.749	0.000
Sarcopenia	DM, S., et al. (2024)	2.267	1.123	4.576	2.283	0.022
Sarcopenia	IY, J., et al. (2018)	2.864	1.920	4.272	5.155	0.000
Sarcopenia	N, Z., et al. (2019)	4.122	2.402	7.074	5.139	0.000
Sarcopenia	Y, K., et al. (2021)	6.965	4.934	9.832	11.034	0.000
Sarcopenia	Pooled	1.780	1.322	2.398	3.798	0.000

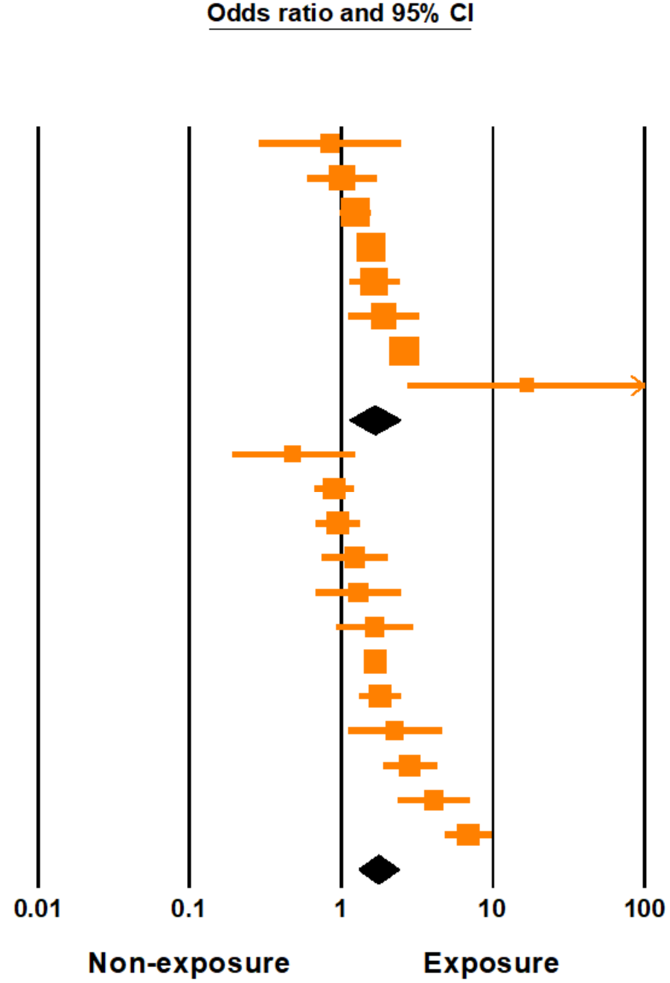

Supplementary Figure S17. Forest plot showing the subgroup analysis comparing individuals with sarcopenia versus those with low lean mass (LLM) in relation to lower urinary tract symptoms (LUTS), using mutually exclusive populations. High heterogeneity was observed ($I^2 = 89.6\%$, df = 19, $p < 0.001$), and the between-group difference was not statistically significant ($Q = 0.052$, df = 1, $p = 0.82$).

Group by Exposure	Study name	Statistics for each study				
		Odds ratio	Lower limit	Upper limit	Z-Value	p-Value
SARC-F>3	AMD, d. S. V., et al. (2024)	5.238	1.301	21.095	2.330	0.020
SARC-F>3	N, W., et al. (2025)	4.540	1.130	18.235	2.132	0.033
SARC-F>3	S, I., et al. (2019)	2.741	1.510	4.977	3.314	0.001
SARC-F>3	Pooled	3.612	1.563	8.348	3.005	0.003
Sarcopenia	B, Z., et al. (2024)	1.691	1.576	1.813	14.681	0.000
Sarcopenia	BW, W., et al. (2023)	0.909	0.677	1.221	-0.633	0.527
Sarcopenia	C, P.-A., et al. (2017)	0.956	0.694	1.318	-0.274	0.784
Sarcopenia	DM, S., et al. (2024)	2.267	1.123	4.576	2.283	0.022
Sarcopenia	HA, P., et al. (2024)	1.233	0.751	2.025	0.827	0.408
Sarcopenia	IY, J., et al. (2018)	2.864	1.920	4.272	5.155	0.000
Sarcopenia	N, Z., et al. (2019)	4.122	2.402	7.074	5.139	0.000
Sarcopenia	RRL, S., et al. (2021)	0.487	0.193	1.227	-1.527	0.127
Sarcopenia	S, K., et al. (2024)	1.313	0.692	2.493	0.833	0.405
Sarcopenia	T, E., et al. (2019)	1.816	1.329	2.480	3.749	0.000
Sarcopenia	Y, K., et al. (2021)	6.965	4.934	9.832	11.034	0.000
Sarcopenia	Y, T., et al. (2019)	1.678	0.941	2.991	1.754	0.079
Sarcopenia	Pooled	1.775	1.290	2.443	3.522	0.000

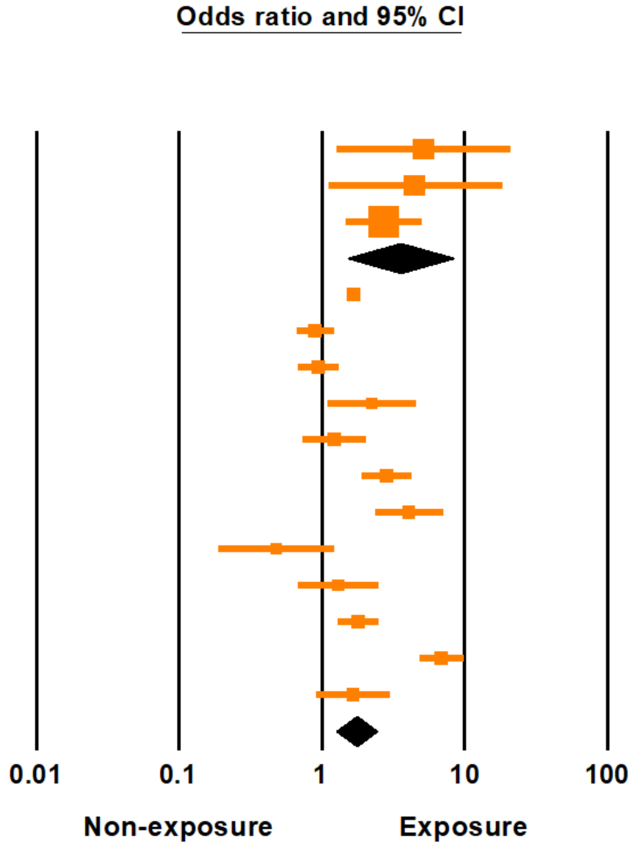

Supplementary Figure S18. Forest plot showing the subgroup analysis comparing individuals with sarcopenia versus those with risk of sarcopenia in relation to lower urinary tract symptoms (LUTS), using mutually exclusive populations. High heterogeneity was observed ($I^2 = 89.0\%$, df = 14, $p < 0.001$), and the between-group difference was not statistically significant (Q = 2.412, df = 1, p = 0.12).

Group by Diagnostic criteria	Study name	Odds ratio	Lower limit	Upper limit	Z-Value	p-Value
Imaging-Based Methods	M, H., et al. (2021)	0.847	0.290	2.479	-0.303	0.762
Imaging-Based Methods	N, O., et al. (2023)	1.917	1.136	3.235	2.437	0.015
Imaging-Based Methods	Pooled	1.442	0.641	3.246	0.885	0.376
Standardized Diagnostic Criteria	C, P.-A., et al. (2017)	0.707	0.440	1.135	-1.435	0.151
Standardized Diagnostic Criteria	W, C., et al. (2025)	1.024	0.607	1.726	0.087	0.930
Standardized Diagnostic Criteria	FX, S., et al. (2024)	1.252	1.004	1.562	1.996	0.046
Standardized Diagnostic Criteria	Y, T., et al. (2019)	1.367	0.943	1.982	1.652	0.099
Standardized Diagnostic Criteria	F, Z. and L. W (2025)	1.594	1.290	1.971	4.316	0.000
Standardized Diagnostic Criteria	Z, Q., et al. (2021)	1.674	1.148	2.441	2.677	0.007
Standardized Diagnostic Criteria	W, S., et al. (2023)	2.623	2.318	2.968	15.288	0.000
Standardized Diagnostic Criteria	G, P., et al. (2020)	17.000	2.765	104.540	3.057	0.002
Standardized Diagnostic Criteria	Pooled	1.509	1.070	2.129	2.344	0.019

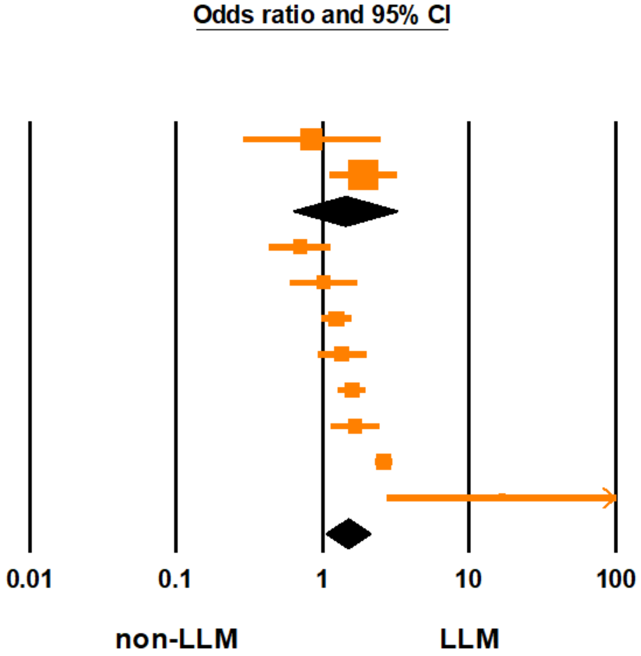

Supplementary Figure S19. Forest plot showing the subgroup analysis comparing individuals with low lean mass (LLM) defined by standardized diagnostic criteria versus imaging-based methods in relation to lower urinary tract symptoms (LUTS). High heterogeneity was observed ($I^2 = 88.1\%$, df = 9, $p < 0.001$), and the between-group difference was not statistically significant (Q = 0.01, df = 1, p = 0.92).

Group by Diagnostic criteria	Study name	Statistics for each study					Odds ratio and 95% CI
		Odds ratio	Lower limit	Upper limit	Z-Value	p-Value	
Standardized Diagnostic Criteria	C, P.-A., et al. (2017)	0.707	0.440	1.135	-1.435	0.151	
Standardized Diagnostic Criteria	W, C., et al. (2025)	1.024	0.607	1.726	0.087	0.930	
Standardized Diagnostic Criteria	FX, S., et al. (2024)	1.252	1.004	1.562	1.996	0.046	
Standardized Diagnostic Criteria	Y, T., et al. (2019)	1.367	0.943	1.982	1.652	0.099	
Standardized Diagnostic Criteria	F, Z. and L. W (2025)	1.594	1.290	1.971	4.316	0.000	
Standardized Diagnostic Criteria	Z, Q., et al. (2021)	1.674	1.148	2.441	2.677	0.007	
Standardized Diagnostic Criteria	W, S., et al. (2023)	2.623	2.318	2.968	15.288	0.000	
Standardized Diagnostic Criteria	G, P., et al. (2020)	17.000	2.765	104.540	3.057	0.002	
Standardized Diagnostic Criteria	Pooled	1.517	1.046	2.198	2.199	0.028	
Surrogate measure	HA, P., et al. (2024)	0.797	0.498	1.277	-0.943	0.346	
Surrogate measure	L, L., et al. (2023)	2.537	2.152	2.991	11.082	0.000	
Surrogate measure	Pooled	1.504	0.743	3.044	1.136	0.256	

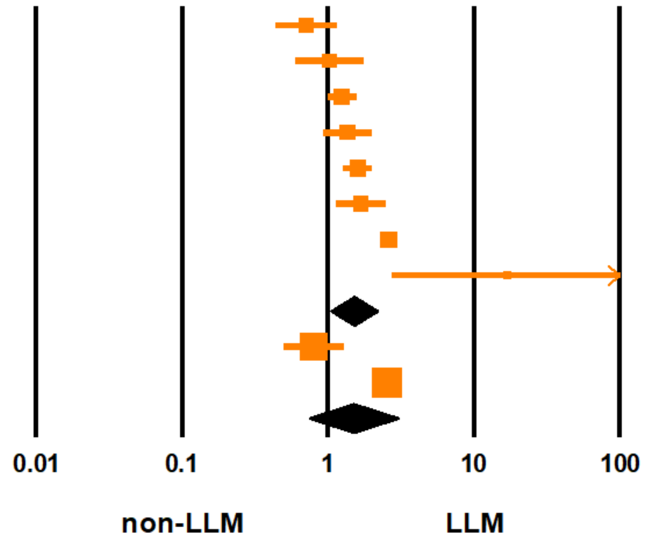

Supplementary Figure S20. Forest plot showing the subgroup analysis comparing individuals with low lean mass (LLM) defined by standardized diagnostic criteria versus a surrogate measure (calf circumference) in relation to lower urinary tract symptoms (LUTS). High heterogeneity was observed ($I^2 = 90.8\%$, df = 9, $p < 0.001$), and the between-group difference was not statistically significant ($Q = 0.00$, df = 1, $p = 0.984$).

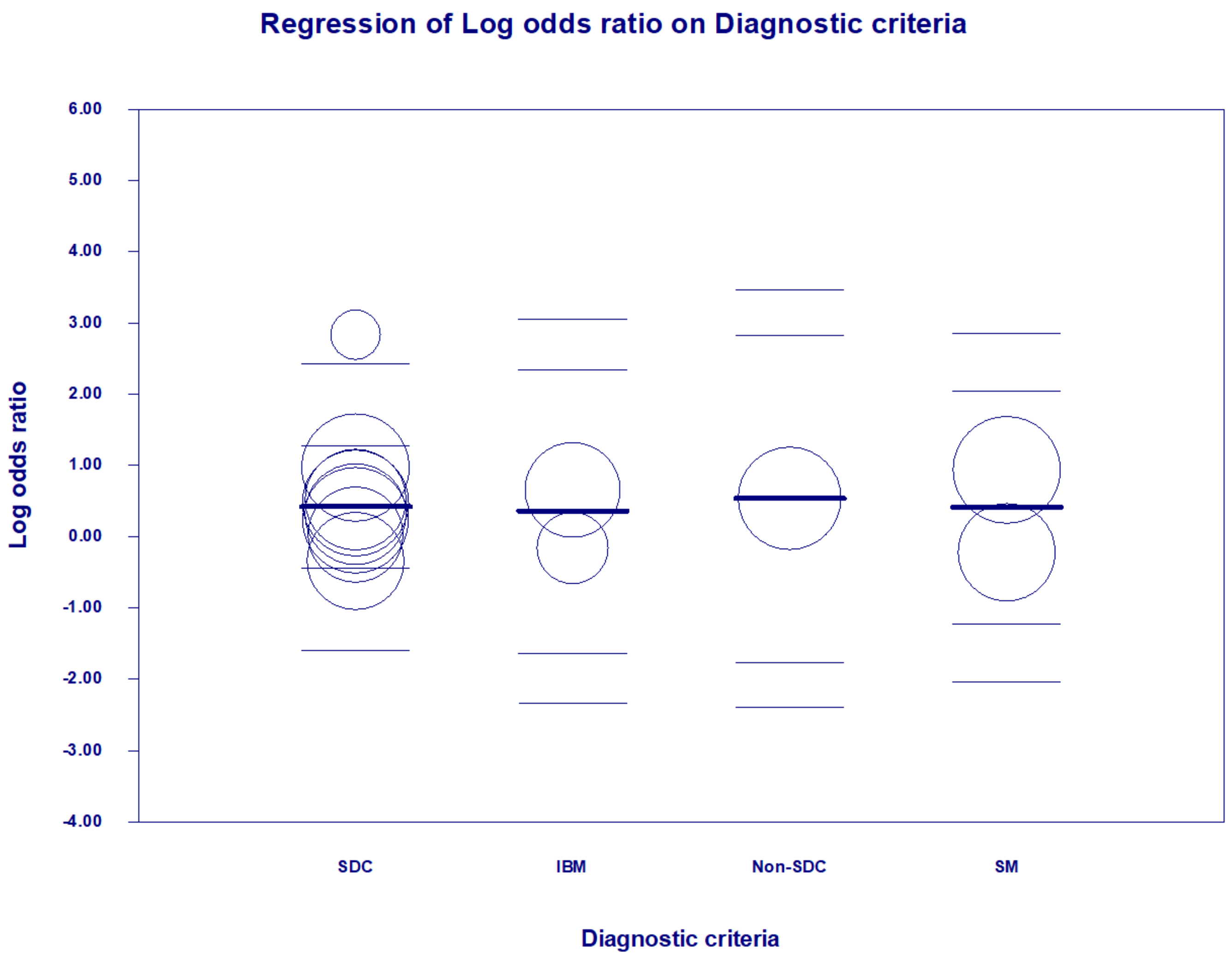

Supplementary Figure S21. Univariate random-effects meta-regression analyses of the odds ratios (ORs) for the association between low lean mass (LLM) and lower urinary tract symptoms (LUTS) across different definitions: standardized diagnostic criteria (SDC), imaging-based methods (IBM), non-standardized diagnostic criteria (Non-SDC), and surrogate measures (SM). Note: Circles represent individual studies, with size proportional to study weight. The central line indicates the fitted meta-regression line, and the outer lines represent the 95% confidence interval.

Group by Populations	Study name	Statistics for each study		
		Event rate	Lower limit	Upper limit
Community-dwelling	B, Z., et al. (2024)	0.110	0.105	0.115
Community-dwelling	Y, T., et al. (2019)	0.216	0.137	0.324
Community-dwelling	IY, J., et al. (2018)	0.463	0.389	0.540
Community-dwelling	BW, W., et al. (2023)	0.468	0.402	0.534
Community-dwelling	T, E., et al. (2019)	0.595	0.530	0.657
Community-dwelling	HA, P., et al. (2024)	0.637	0.545	0.720
Community-dwelling	RRL, S., et al. (2021)	0.640	0.440	0.801
Community-dwelling	DM, S., et al. (2024)	0.708	0.566	0.819
Community-dwelling	Pooled	0.458	0.247	0.686
Institutionalized Population	S, K., et al. (2024)	0.304	0.262	0.351
Institutionalized Population	N, Z., et al. (2019)	0.444	0.340	0.554
Institutionalized Population	Y, K., et al. (2021)	0.461	0.416	0.507
Institutionalized Population	Pooled	0.401	0.126	0.756

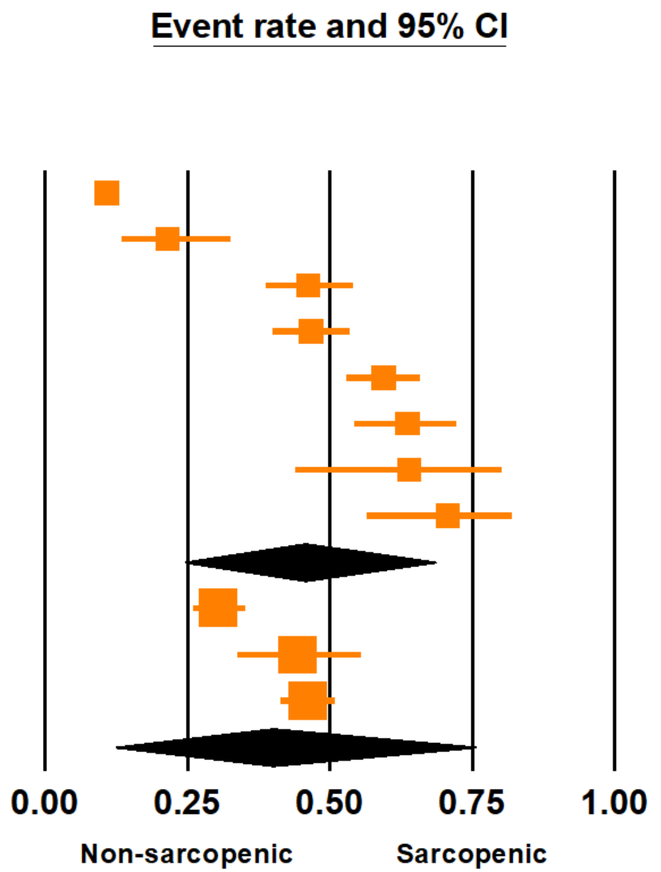

Supplementary Figure S22. Forest plot showing the subgroup analysis of pooled prevalence of lower urinary tract symptoms in community-dwelling versus institutionalized populations. Substantial heterogeneity was observed within subgroups ($I^2 = 99.2\%$, $p < 0.001$, Cochran's Q test), with no significant difference between subgroups ($Q = 0.065$, $df = 1$, $p = 0.799$).
